# Tetramethylpyrazine and renal ischemia-reperfusion injury: a systematic review and meta-analysis of preclinical studies

**DOI:** 10.3389/fphar.2025.1559314

**Published:** 2025-06-02

**Authors:** Zhongmei Fu, Lianyan Jiang, Xiaojuan Su, Hua Wang, Mingquan Li

**Affiliations:** ^1^ Clinical Medical College, Chengdu University of Traditional Chinese Medicine, Chengdu, China; ^2^ Ophthalmology, Hubei provincial hospital of Traditional Chinese Medicine, Wuhan, China; ^3^ Department of pharmacy, Kunming Maternal and Child Health Care Hospital, Kunming, China; ^4^ Hospital of Chengdu University of Traditional Chinese Medicine, Chengdu, China

**Keywords:** tetramethylpyrazine, renal ischemia-reperfusion injury, preclinical study, meta-analysis, systematic review

## Abstract

**Objectives:**

The aim of this systematic review and meta-analysis is to synthesize the effects and mechanisms of Tetramethylpyrazine (TMP) on renal outcomes in animal models of renal I/R injury.

**Methods:**

Animal studies from seven electronic databases were searched up to October 2024. The risk of bias of the selected studies was assessed using the SYRCLE risk of bias tool. Standardized mean difference (SMD) or mean difference (MD) were estimated for the effects of TMP on serum creatinine (Scr), blood urea nitrogen (BUN), oxidative stress, inflammation and apoptotic. Random-effects models were used to summarize results. Heterogeneity was expressed as I^2^. Subgroup analyses were used to clarify the sources of heterogeneity. Egger’s test was used to assess publication bias. Sensitivity analyses were used to assess the robustness of the results. Statistical analysis was performed using RevMan 5.3 software.

**Results:**

Thirty studies involving 559 animals were identified for analysis. TMP treatment significantly decreased Scr (SMD = 2.35, 95% CI: −2.97 to −1.72, P < 0.05), BUN (SMD = −2.4, 95% CI: −3.01 to −1.79, P < 0.05). TMP treatment significantly improved oxidative stress expression (i.e., SOD, MDA, GSHPX, CAT, TAC) and alleviated inflammation levels (i.e., TNF-α, ICAM-1, IL-6, IL-10, NLRP3). TMP treatment also regulate the expression of apoptosis-related proteins (i.e., bcl-2, Bax, caspase 3, Caspase-12 and GRP78).

**Conclusion:**

TMP could improve renal outcomes and alleviate injury through multiple signaling pathways. However, positive results should be treated with caution due to the significant heterogeneity and poor quality of the included studies.

**Systematic Review Registration:**

CRD420251017081.

## 1 Introduction

Acute kidney injury (AKI) is a clinical syndrome characterized by a rapid and significant decline in kidney function. Renal ischemia-reperfusion (I/R) injury is a major cause of AKI, affecting approximately 10%–15% of hospitalized patients and is characterized by complex pathophysiological processes including inflammation, oxidative stress, and apoptosis (Mustafa et al., 2024; Tiwari et al., 2024). Severe ischemic AKI-induced tubular maladaptive repair leading to long-term functional defects and progressive pathological transition to chronic kidney disease (CKD), which is an important risk factor for the development of CKD (Zheng et al., 2021; Basile et al., 2016; Prem et al., 2025).Currently, the main treatment strategy for renal IR injury remains renal replacement therapy. However, renal replacement therapy has been observed to have serious side effects, such as hypophosphatemia, hypokalemia, and hypotension (Eisenstein, 2023). Thus, novel treatment strategies for renal IR injury need to be urgently developed.

*Ligusticum chuanxiong* Hort (known as Chuanxiong in China, CX), is one of the most widely used herbs in traditional Chinese medicine and was first reported in Shennong’s Classic of Material Medical. In traditional Chinese medicine, Chuanxiong is used to invigorate blood circulation, remove blood stasis, and restore blood circulation (Chen et al., 2018; Lin et al., 2022). Tetramethylpyrazine (TMP), the key active components of the Chuanxiong, has been proven to possess several pharmacological properties and has been used to treat a variety of diseases with excellent therapeutic effects, such as cardiovascular and cerebrovascular diseases, liver and kidney injury, cancer, and particularly ischemic diseases, and has achieved good therapeutic results (Qi et al., 2024; Wang et al., 2024; Zhang et al., 2023; Zeng et al., 2013). This broad efficacy is attributed to its rapid absorption, extensive distribution, minimal cumulative toxicity, and diverse pharmacological properties such as antioxidant, anti-inflammatory, and cytoprotective effects, along with its capacity to improve microcirculation (Lin et al., 2022; Qi et al., 2024). TMP is a very promising drug, and its therapeutic mechanism involves multiple targets, multiple pathways and bidirectional regulation. Preclinical studies indicate that TMP mitigates renal I/R injury by attenuating oxidative stress-related injury (Feng et al., 2011; Sun et al., 2002). However, existing literature on the therapeutic effects of TMP on renal I/R injury is fragmented, with different studies emphasizing varying metrics. This fragmentation has led to ongoing uncertainty about its overall efficacy. Therefore, a comprehensive quantitative analysis of the mechanism of action and efficacy of TMP in the treatment of renal I/R injury is necessary. This study aims to systematically evaluate and quantify the interventional effects of TMP in animal models with renal I/R injury, focusing on multiple mechanisms and efficacy indicators based on available data.

## 2 Methods

Two authors independently conducted a comprehensive search for animal studies investigating TMP in the context of renal ischemia-reperfusion injury, covering the period from the inception of the respective databases to October 2024. The search encompassed seven databases: PubMed, Embase, Web of Science, China National Knowledge Infrastructure (CNKI), VIP Information Chinese Periodical Service Platform (VIP), Wanfang Data Knowledge Service Platform (Wanfang), and China Biology Medicine Disc (CBM), without imposing language limitations. Discrepancies or conflicts arising during the search process were resolved with a third researcher. The following are the key search strings: participants (“ischemia/reperfusion,” “ischemia-reperfusion,” “ischemia reperfusion,” “reperfusion,” “I/R,” “IRI,” “Kidney,” “Renal,” “Damage,” “Injury”), Intervention (“tetramethylpyrazine,” “chuanxiongzine,” “ligustrazine,” “TMPZ,” “tetramethylpyrazine hydrochloride,” “Liqustrazine,” “tetramethyl pyrazine”). The specific retrieval strategies are listed in Supplementary Table S1.

### 2.1 Study selection

#### 2.1.1 Inclusion criteria

(1) Participants: This review included animal models subjected to renal I/R injury, with no restrictions on species, sex, model type (unilateral/bilateral), or the duration of ischemia and reperfusion.
(2) Intervention: TMP was evaluated with flexibility in terms of dosage, route of administration, timing of application, and dosing frequency
(3) Comparison: Control groups comprised animals administered an equivalent volume of non-functional substances, such as water or normal saline, delivered in the same manner as the experimental group or animals that received no treatment.
(4) Outcomes: The primary outcome was the restoration of serum creatinine (Scr) and blood urea nitrogen (BUN) levels, while secondary outcomes were the underlying mechanisms by which TMP mitigates renal I/R injury. Outcome measurements were not restricted by methods, and all data collected were treated as continuous variables.

#### 2.1.2 Exclusion criteria

(1) The studies excluding animal experiments, clinical trials, *in vitro* models, case reports, reviews, comments, conferences, and abstracts.
(2) Animal models of non-renal I/R injury, studies involving animals with comorbidities, AKI models in transplanted kidneys, folic acid-induced models, and genetically modified models were excluded.
(3) Research focusing on other treatment drugs, such as TMP combined with other therapies, natural analogs of TMP, or TMP derivatives without standalone TMP administration was also excluded.
(4) Studies without distinct control groups.
(5) Studies without full text or relevant outcome data.
(6) Studies containing duplicated data or publications.

### 2.2 Data extraction

Two authors independently conducted an initial screening of titles and abstracts using predetermined inclusion and exclusion criteria to identify relevant studies. Full texts of the selected studies were systematically reviewed to confirm eligibility for inclusion in the meta-analysis. Any disagreements between the two authors were resolved through consultation with the corresponding author. For each included study, data were extracted and recorded in an Excel spreadsheet, covering the following information: (1) first author and year of publication; (2) animal characteristics, including species, sex, age, and body weight; (3) method used to establish the renal I/R injury model; (4) anesthetic methods (mode of administration and dosage); (5) duration of ischemia and reperfusion; (6) timing of TMP application (pre and/or during ischemia [ischemia], pre and/or during reperfusion [reperfusion], or both ischemia and reperfusion [continuous]), along with route of administration, dosage and times of treatment; (7) outcome indicators. Outcome indicators presented graphically in the study were digitized using GetData Graph Digitizer software (version 2.26). For studies reporting multiple time points, data from the last time point were extracted.

### 2.3 Risk of bias assessment

Two researchers independently evaluated the methodological quality of the included studies using the Center for Systematic Review Centre for Laboratory animal Experimentation (SYRCLE) bias risk tool. This evaluation examined potential biases in 10 areas: (1) sequence generation; (2) baseline characteristics; (3) allocation concealment; (4) random housing; (5) blinding of animal breeders and researchers; (6) random outcome assessment; (7) blinding of outcome evaluators; (8) incomplete outcome data; (9) selective outcome reporting; and (10) other potential sources of bias. Discrepancies between the two researchers were resolved through consultation with a third author.

### 2.4 Data synthesis and analysis

The RevMan software (version 5.4) was used to analyze the extracted data. Standard mean difference (SMD) with 95% confidence intervals (95% CI) was employed to quantify the effect size of TMP intervention in renal I/R injury, as the extracted data in the included studies were continuous. We used Cochran’s Q statistic and I^2^ to determine heterogeneity. I^2^ > 50% and PQ−test <0.1 indicate significant heterogeneity. At this time, the random effects model is adopted, while the fixed effects model is used (Borenstein et al., 2020; Higgins et al., 2003). In order to address high heterogeneity, subgroup analyses were conducted based on factors such as species, renal I/R injury model (unilateral/bilateral), anesthetic methods, application time of TMP, dosage, route of administration, and frequency of treatments to evaluate their influence on heterogeneity and estimated effect sizes. Publication bias was assessed using Egger’s test, while sensitivity analyses were performed to evaluate the stability of the findings.

## 3 Results

### 3.1 Study selection

**FIGURE 1:**
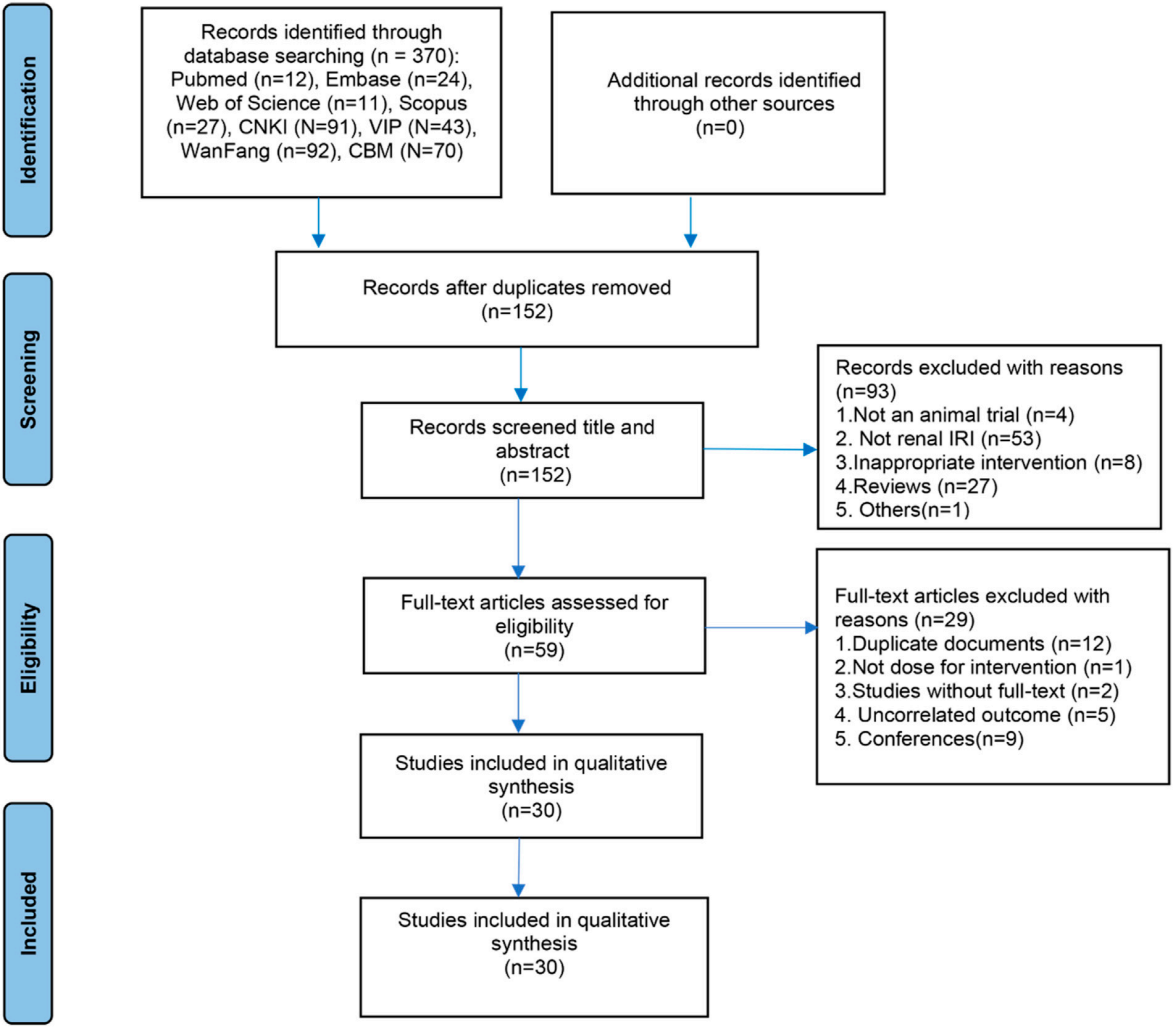
Flow diagram depicting the selection of studies.

A total of 370 articles were initially retrieved from eight databases, including PubMed, Embase, Web of Science, Scopus, CNKI, VIP, Wanfang, and CBM. Following a systematic screening of titles, abstracts, and full texts based on predetermined inclusion and exclusion criteria, 30 studies were deemed eligible for inclusion in this systematic review and meta-analysis. The detailed study selection process is illustrated in Figure 1.

### 3.2 Study characteristics

A total of 30 studies involving 559 animal models of renal I/R injury were included in the analysis. Among these, 22 studies used rats (Sun et al., 2002; Wang et al., 2011; Ye et al., 2007; Wang et al., 2019; Wang and Xu, 2016; Li, 2007; Wan et al., 2011; Liu et al., 2008; Ma et al., 2007; Zhu et al., 2003; Pan et al., 2004; Zhou, 2017; Miao et al., 2000; Jiang et al., 2020; Liu, 2019; Hai and Cui, 2005; Wang et al., 2008; Shang et al., 2006; Kong et al., 1998; Wang and Shi, 2005; Sun et al., 2020; Wang et al., 2005), five used mice (Feng et al., 2011; Feng et al., 2004; Liu et al., 2002; Huo et al., 2004; Xie and Lu, 2020), and three used rabbits as the animal model (Chen et al., 2013; Li et al., 1994; Chen et al., 2009). All but one study (Miao et al., 2000) reported the body weights of the animals, with rats weighing 170–320 g, mice 19–33 g, and rabbits 1.7–3 kg. Regarding the anesthetic drugs employed, sodium pentobarbital was used in 17 studies (Sun et al., 2002; Wang et al., 2019; Wang and Xu, 2016; Liu et al., 2008; Pan et al., 2004; Miao et al., 2000; Jiang et al., 2020; Liu, 2019; Hai and Cui, 2005; Wang et al., 2008; Kong et al., 1998; Wang and Shi, 2005; Sun et al., 2020; Liu et al., 2002; Huo et al., 2004; Xie and Lu, 2020; Li et al., 1994), chloral hydrate was used in six studies (Wang et al., 2011; Li, 2007; Wan et al., 2011; Ma et al., 2007; Zhou, 2017; Shang et al., 2006), urethane was used in three studies (Zhu et al., 2003; Chen et al., 2013; Chen et al., 2009), and ketamine hydrochloride was used in one study (Wang et al., 2005); however, three studies did not report the use of anesthetic drugs (Feng et al., 2011; Ye et al., 2007; Feng et al., 2004). For modeling methods, one study used unilateral I/R injury (Ma et al., 2007), while bilateral I/R injury and unilateral I/R injury with contralateral nephrectomy (uIRIx) were utilized in 18 (Sun et al., 2002; Wang et al., 2011; Wang et al., 2019; Wan et al., 2011; Liu et al., 2008; Zhu et al., 2003; Pan et al., 2004; Zhou, 2017; Jiang et al., 2020; Liu, 2019; Hai and Cui, 2005; Wang et al., 2008; Wang and Shi, 2005; Sun et al., 2020; Xie and Lu, 2020; Chen et al., 2013; Li et al., 1994; Chen et al., 2009) and 11 studies (Feng et al., 2011; Ye et al., 2007; Wang and Xu, 2016; Li, 2007; Miao et al., 2000; Shang et al., 2006; Kong et al., 1998; Wang et al., 2005; Feng et al., 2004; Liu et al., 2002; Huo et al., 2004), respectively. The ischemia duration ranged from 30 to 60 min, and reperfusion time ranged from 30 min to 7 days. In the included studies, TMP was applied during ischemia in 18 studies (Feng et al., 2011; Sun et al., 2002; Wang et al., 2011; Ye et al., 2007; Li, 2007; Wan et al., 2011; Ma et al., 2007; Zhu et al., 2003; Miao et al., 2000; Wang et al., 2008; Shang et al., 2006; Wang and Shi, 2005; Wang et al., 2005; Feng et al., 2004; Liu et al., 2002; Huo et al., 2004; Xie and Lu, 2020; Li et al., 1994), during reperfusion in 7 studies (Wang et al., 2019; Liu et al., 2008; Zhou, 2017; Jiang et al., 2020; Liu, 2019; Hai and Cui, 2005; Sun et al., 2020), and continuously in five studies (Wang and Xu, 2016; Pan et al., 2004; Kong et al., 1998; Chen et al., 2013; Chen et al., 2009). The administered drug doses ranged from 10 to 200 mg, with three studies (Wang et al., 2019; Wang and Xu, 2016; Ma et al., 2007) employing a dose gradient. TMP was administered orally in only one study (Shang et al., 2006), while most others utilized intraperitoneal or intravenous administration. Detailed characteristics of the included studies are summarized in Table 1.

**TABLE 1:** Basic characteristics of included studies in the meta-analysis.

Study ID	Species,Sex or Weight at time of I/R	Renal I/R Injury	Occluded Artery	Anesthetic Regimen	Duration of ischemia	Duration of reperfusion	Time of application	Route, Dosage	Control	Outcomes	Intergroup differences
Jiang et al. (2020)	Rat,M 280–300 g	Bilateral	Both renal arteries	i.p.: pentobarbital sodium (50 mg/kg)	50 min	24 h	Reperfusion	i.p. 40 mg/kg	saline	Body weight (g) Scr BUN TNF-α IL-6 MCP-1 CD68^+^ NOD2 Caspase 3/cleaved caspase 3	P > 0.05 P < 0.05 P < 0.05 P < 0.05 P < 0.05 P < 0.05 P < 0.05 P < 0.05 P < 0.05
Sun et al. (2002)	Rat,NA 200–250 g	Bilateral	Both renal arteries	i.p.: pentobarbital sodium (30 mg/kg)	60 min	24 h	Ischemia	i.p. 80 mg/kg	Saline	SOD MDA ET-1	P < 0.05 P < 0.05 P < 0.05
Sun et al. (2020)	Rat,M 200–250 g	Bilateral	Both renal arteries	i.p.: 3% pentobarbital sodium (50 mg/kg)	45 min	24 h	Reperfusion	i.p. 40 mg/kg	saline	KIM-1 Scr BUN TNF-α IL-6 Caspase 3 NLRP3	P < 0.05 P < 0.05 P < 0.05 P < 0.05 P < 0.05 P < 0.05 P < 0.05
Feng et al. (2011)	Mice,M 20–25 g	Unilateral	Left renal artery	NA	50 min	24 h	Ischemia	i.p. 80 mg/kg	saline	MDA SOD MPO TNF-α ICAM-1	P < 0.05 P < 0.05 P < 0.05 P < 0.05 P < 0.05
Feng et al. (2004)	Mice,M 20–25 g	Unilateral	Left renal artery	NA	50 min	24 h	Ischemia	i.p. 80 mg/kg	No treatment	SCR BUN MDA SOD ICAM-1 Bcl-2	P < 0.05 P < 0.05 P < 0.05 P < 0.05 P < 0.05 P < 0.05
Ma et al. (2007)	Rat,M/F 180–220 g	Unilateral	Right renal pedicle	i.p.: 6%Chloral hydrate (1 mL/piece)	60 min	24 h	Ischemia	i.v.15 mg/kg, 30 mg/kg, 45 mg/kg, 60 mg/kg, 90 mg/kg	0.9% sodium chloride solution	Scr BUN INOs	P < 0.05 P < 0.05 P < 0.05
Hai and Cui (2005)	Rat,M 180–220 g	Bilateral	Both renal pedicles	i.p.: 3% pentobarbital sodium (40 mg/kg)	45 min	24 h	Reperfusion	i.v. 80 mg/kg	Saline	Scr BUN MDA SOD	P < 0.05 P < 0.05 P < 0.05 P < 0.05
Wang and Xu (2016)	Rat,M 250–280 g	Unilateral	Left renal pedicle	i.p.:pentobarbital sodium	60 min	24 h	Continuous	i.p.20 mg/kg, 30 mg/kg, 40 mg/kg	No treatment	Scr BUN MDA SOD TNF-α	P < 0.05 P < 0.05 P < 0.05 P < 0.05 P < 0.05
Wang et al. (2011)	Rat,M 225–265 g	Bilateral	Both renal pedicles	i.p.: 10%Chloral hydrate (3 mL/kg)	45 min	24 h	Ischemia	i.p. 20 mg/kg	No treatment	Scr BUN GRP78 Caspase-12 Caspase-3	P < 0.05 P < 0.05 P < 0.05 P < 0.05 P < 0.05
Li (2007)	Rat,M 200–250 g	Unilateral	Left renal artery	i.p.: 10%Chloral hydrate (3 mL/kg)	45 min	24 h	Ischemia	i.v. 40 mg/kg	Saline	Scr BUN MDA SOD TNF-α IL-10	P < 0.05 P < 0.05 P < 0.05 P < 0.05 P < 0.05 P < 0.05
Wang et al. (2008)	Rat,M/F 200–240 g	Bilateral	Both renal arteries	i.p.:30 g/L pentobarbital sodium (30–50 mg/kg)	45 min	24 h	Ischemia	i.p. 32 mg/kg	No treatment	Scr BUN Bcl-2 Bax	P < 0.05 P < 0.05 P < 0.05 P < 0.05
Miao et al. (2000)	Rat,M NA	Unilateral	Left renal pedicle	i.p.:pentobarbital sodium (45 mg/kg)	60 min	24 h	Ischemia	i.v. 4 mg^−1^/只	Saline	NO NOS MDA	P < 0.05 P < 0.05 P < 0.05
Wang et al. (2005)	Rat,M 250–270 g	Unilateral	Right renal artery	i.p.: Ketamine hydrochloride (100 mg/kg)	60 min	7 days	Ischemia	i.v. 80 mg/kg	Saline	Scr BUN	P < 0.05 P < 0.05
Wang et al. (2019)	Rat,NA 250–320 g	Bilateral	Both renal arteries	i.p.:1% pentobarbital sodium	45 min	24 h	Reperfusion	i.p.10 mg/kg, 15 mg/kg, 20 mg/kg	No treatment	Scr BUN MDA SOD TNF-α	P < 0.05 P < 0.05 P < 0.05 P < 0.05 P < 0.05
Zhu et al. (2003)	Rat,M 235–265 g	Bilateral	Both renal arteries	i.p.: 20%Urethane (60 mg/kg)	60 min	24 h	Ischemia	i.v. 40 mg/kg	No treatment	c-fos bcl-2 ICAM-1	P < 0.05 P < 0.05 P < 0.05
Liu (2019)	Rat,M 180–200 g	Bilateral	Both renal arteries	i.p.: 3% pentobarbital sodium (80 mg/kg)	45 min	24 h	Reperfusion	40 mg/kg	No treatment	Scr BUN NLRP3	P < 0.05 P < 0.05 P < 0.05
Wang et al. (2005)	Rat,M 232–268 g	Bilateral	Both renal pedicles	i.p.: 3% pentobarbital sodium (60 mg/kg)	30 min	2 h	Ischemia	i.v. 1 mL·100 g^−1^h^−1^	Saline	Scr BUN Urine volume Ccr	P < 0.05 P < 0.05 P < 0.05 P < 0.05
Xie and Lu (2020)	Mice,M 19–21 g	Bilateral	Both renal pedicles	i.p.: 2% pentobarbital sodium (40 mg/kg)	35 min	24 h	Ischemia	i.p. 60 mg/kg	Saline	Scr BUN MDA SOD TNF-α IL-10	P < 0.05 P < 0.05 P < 0.05 P < 0.05 P < 0.05 P < 0.05
Wan et al. (2011)	Rat,M/F 225–265 g	Bilateral	Both renal pedicles	i.p.: 10%Chloral hydrate l (3 mL/kg)	45 min	24 h	Ischemia	i.p. 20 mg/kg	No treatment	Scr BUN GRP78 Caspase-12	P < 0.05 P < 0.05 P < 0.05 P < 0.05
Huo et al. (2004)	Mice,M 27–33 g	Unilateral	Right renal pedicle	i.p.: 0.2% pentobarbital sodium (40 mg/kg)	30 min	24 h	Ischemia	i.p. 100 mg/kg	Saline	Scr Bun MDA SOD	P < 0.05 P < 0.05 P < 0.05 P < 0.05
Liu et al. (2002)	Mice,M 27–33 g	Unilateral	Right renal pedicle	i.p.: 0.2% pentobarbital sodium (40 mg/kg)	30 min	24 h	Ischemia	i.p. 100 mg/kg	Saline	Scr BUN MDA NO	P < 0.05 P < 0.05 P < 0.05 P < 0.05
Kong et al. (1998)	Rat,M 250–300 g	Unilateral	Left renal pedicle	i.p.: 3% pentobarbital sodium (40 mg/kg)	60 min	24 h	Continuous	i.p. 32 mg/kg	No treatment	Scr BUN MDA	P < 0.05 P < 0.05 P < 0.05
Shang et al. (2006)	Rat,M 280–320 g	Unilateral	Left renal artery	i.p.: 10%Chloral hydrate l (3 mg/kg)	60 min	24 h	Ischemia	Intragastric 200 mg/kg	Saline	Scr Bun MDA SOD GSHPX	P < 0.05 P < 0.05 P < 0.05 P < 0.05 P < 0.05
Pan et al. (2004)	Rat,M 190–250 g	Bilateral	Both renal arteries	i.p.: pentobarbital sodium (35 mg/kg)	45 min	24 h	Continuous	i.p. 36 mg/kg	Saline	Scr BUN NO ET	P < 0.05 P < 0.05 P < 0.05 P < 0.05
Zhou (2017)	Rat,M 250–300 g	Bilateral	Both renal arteries	i.p.: 10%Chloral hydrate l (3–5 mL/kg)	60 min	24 h	Reperfusion	i.p. 100 mg/kg	No treatment	Scr BUN K VEGF	P > 0.05 P < 0.05 P < 0.05 P < 0.05
Ye et al. (2007)	Rat,M 170–270 g	Unilateral	Left renal arteriovenous	NA	60 min	24 h	Ischemia	i.v. 100 mg/kg	Saline	Scr MDA SOD	P < 0.05 P < 0.05 P < 0.05
Liu et al. (2008)	Rat,F 222.3–242.7 g	Unilateral	Left renal artery	i.p.:30 g/L pentobarbital sodium (50 mg/kg)	45 min	24 h	Reperfusion	i.p. 32 mg/kg	No treatment	Scr BUN SOD MDA CAT TAC	P < 0.05 P < 0.05 P < 0.05 P < 0.05 P < 0.05 P < 0.05
Chen et al. (2013)	Rabbit,M/F,1.7–2.7 kg	Bilateral	Both renal arteries	i.v.: 20%Urethane (4 mL/kg)	60 min	5 h	Continuous	i.v. 200 mg/kg	Saline	NO ET-1	P < 0.05 P < 0.05
Chen et al. (2009)	Rabbit,M/F,1.7–2.7 kg	Bilateral	Both renal arteries	i.v.: 20%Urethane (4 mL/kg)	60 min	5 h	Continuous	i.v. 200 mg/kg	Saline	SOD MDA XO	P < 0.05 P < 0.05 P < 0.05
Li et al. (1994)	Rabbit,M,2.5–3 kg	Bilateral	Both renal arteries	i.v.: 3% pentobarbital sodium	60 min	30 min	Ischemia	i.v. 40 mg/kg	Saline	Scr SOD MDA	P < 0.05 P < 0.05 P < 0.05

Abbreviations:Scr: serum creatinine; BUN: blood urea nitrogen; GSH-Px: glutathione peroxidase; IL-6: interleukin-6; IL-10: interleukin-10; KIM-1: Kidney Injury Molecule-1; MCP-1: monocyte chemoattractant protein-1; MDA: malondialdehyde; SOD: superoxide dismutase; TNF-α: tumor necrosis factor-α; XO: xanthine oxidase; ET-1: endothelin-1; NO: nitric oxide; CAT: catalase; TAC: total anti-oxidant capacity; VEGF: vascular endothelial growth factor; GRP78: glucose regulating protein78; NLRP3: NOD-like receptor pyrin domain-containing protein 3; Caspase-12: cysteiny-12 aspartate specific protease; Caspase-3: cysteiny-3, aspartate specific protease; NOD2: nucleotide-binding oligomerization domain-containing 2; bcl-2: B-cell lymphoma-2; ICAM-1: intercellular cell adhesion molecule-1; Bax: bcl2-associated x; ischemia: pre and/or during ischemia; reperfusion: pre and/or during reperfusion; continuous: both ischemia and reperfusion i.v: IntraVenous; i.p: IntraPeritoneal.

### 3.3 Risk of bias and quality of the included studies

Among the included 30 studies, none specified a method for random allocation or detailed the process for allocation concealment. Only one study (Pan et al., 2004) reported similar baseline characteristics between groups. Nine studies (Feng et al., 2011; Sun et al., 2002; Wang and Xu, 2016; Zhou, 2017; Jiang et al., 2020; Liu, 2019; Wang et al., 2008; Sun et al., 2020; Xie and Lu, 2020) reported similar housing and environmental conditions. Regarding the randomization of outcome assessments, all studies except two (Liu et al., 2002; Huo et al., 2004) were classified as high-risk. None of the studies reported whether animal keepers, researchers, or outcome evaluators were blinded. Nevertheless, all included studies adhered to their predetermined protocols and reported results in full accordance with their study prospectuses. No additional sources of bias were identified. A detailed quality assessment of the included studies is presented in Table 2.

**TABLE 2:** Risk of bias of included studies.

Study	(1)	(2)	(3)	(4)	(5)	(6)	(7)	(8)	(9)	(10)	Scores
Jiang et al. (2020)	U	U	U	Y	U	N	U	Y	Y	Y	4
Sun et al. (2002)	U	U	U	Y	U	N	U	Y	Y	Y	4
Sun et al. (2020)	U	U	U	Y	U	N	U	Y	Y	Y	4
Feng et al. (2011)	U	U	U	Y	U	N	U	Y	Y	Y	4
Feng et al. (2004)	U	U	U	U	U	N	U	Y	Y	Y	3
Ma et al. (2007)	U	U	U	U	U	N	U	Y	Y	Y	3
Hai and Cui (2005)	U	U	U	U	U	N	U	Y	Y	Y	3
Wang and Xu (2016)	U	U	U	Y	U	N	U	Y	Y	Y	4
Wang et al. (2011)	U	U	U	U	U	N	U	Y	Y	Y	3
Li (2007)	U	U	U	U	U	N	U	Y	Y	Y	3
Wang et al. (2008)	U	U	U	Y	U	N	U	Y	Y	Y	4
Miao et al. (2000)	U	U	U	U	U	N	U	Y	Y	Y	3
Wang et al. (2005)	U	U	U	U	U	N	U	Y	Y	Y	3
Wang et al. (2019)	U	U	U	U	U	N	U	Y	Y	Y	3
Zhu et al. (2003)	U	U	U	U	U	N	U	Y	Y	Y	3
Liu (2019)	U	U	U	Y	U	N	U	Y	Y	Y	4
Wang et al. (2005)	U	U	U	U	U	N	U	Y	Y	Y	3
Xie and Lu (2020)	U	U	U	Y	U	N	U	Y	Y	Y	4
Wan et al. (2011)	U	U	U	U	U	N	U	Y	Y	Y	3
Huo et al. (2004)	U	U	U	U	U	U	U	Y	Y	Y	3
Liu et al. (2002)	U	U	U	U	U	U	U	Y	Y	Y	3
Kong et al. (1998)	U	U	U	U	U	N	U	Y	Y	Y	3
Shang et al. (2006)	U	U	U	U	U	N	U	Y	Y	Y	3
Pan et al. (2004)	U	Y	U	U	U	N	U	Y	Y	Y	4
Zhou (2017)	Y	U	U	Y	U	N	U	Y	Y	Y	5
Ye et al. (2007)	U	U	U	U	U	N	U	Y	Y	Y	3
Liu et al. (2008)	U	U	U	U	U	N	U	Y	Y	Y	3
Chen et al. (2013)	U	U	U	U	U	N	U	Y	Y	Y	3
Chen et al. (2009)	U	U	U	U	U	N	U	Y	Y	Y	3
Li et al. (1994)	U	U	U	U	U	N	U	Y	Y	Y	3

(1) Sequence gneration (2) baseline characteristics (3) allocation concealment (4) random housing (5) blinding (performance bias) (6) random outcome assessment (7) blinding (detection bias) (8) incomplete outcome data (9) selective outcome reporting (10) other sources of bias.

Y: yes; N: no; U: unclear.

### 3.4 Effects of TMP on kidney function

**FIGURE 2:**
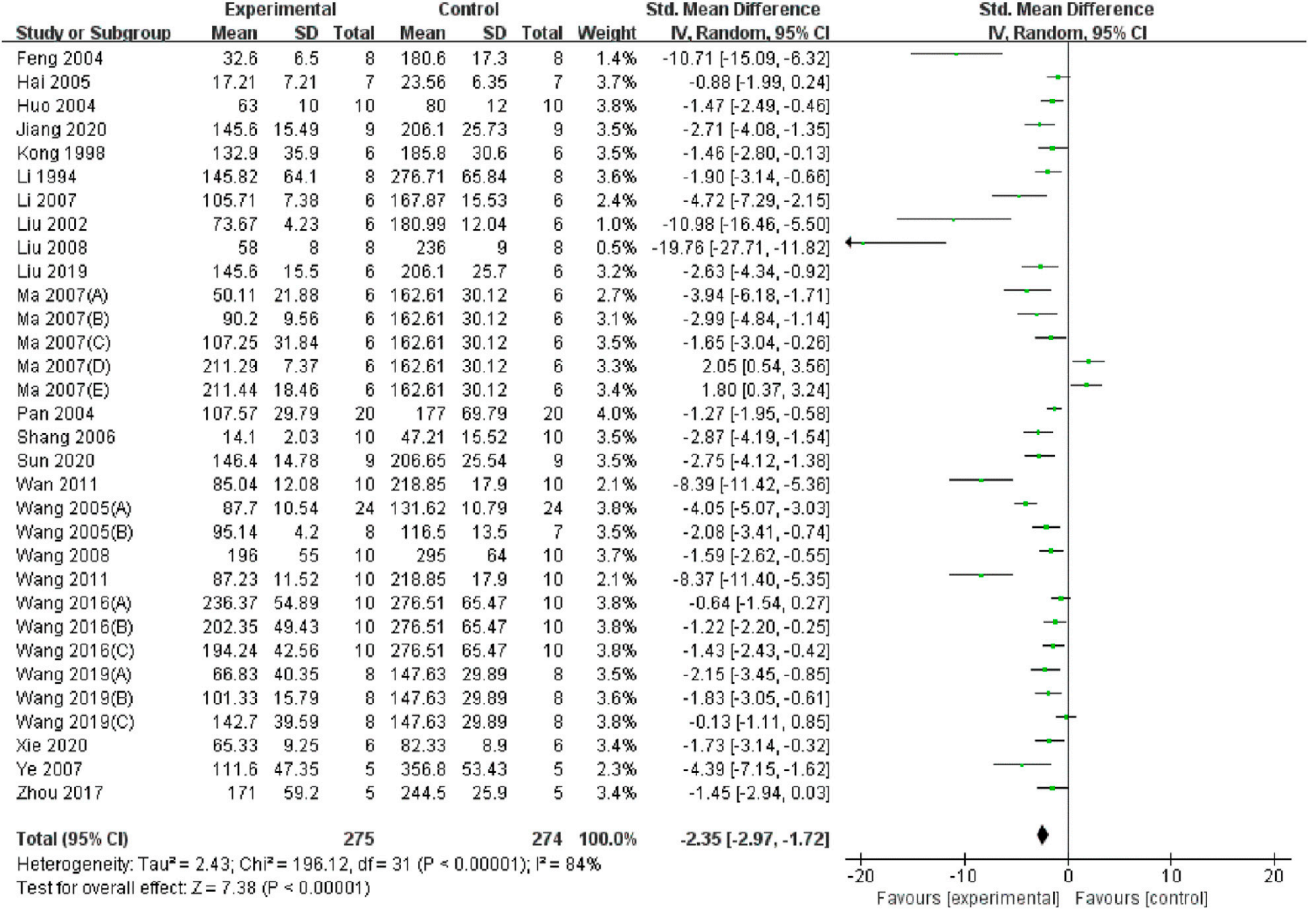
Forest plot showing the pooled effect estimation of TMP on Scr.

The summary analysis revealed that, compared to the control group, TMP significantly decreased Scr levels (SMD = −2.35, 95% CI: −2.97 to −1.72, P < 0.05; heterogeneity: I^2^ = 85%; PQ test < 0.05, Figure 2). To address this heterogeneity, subgroup analysis was performed, which indicated no significant differences in the effects of TMP on species (SMD: −2.2 vs. −5.00 vs. −1.90, P > 0.05), I/R injury model (SMD: −2.89 vs. −2.39 vs. −0.87, P > 0.05), renal ischemia duration (SMD: −2.68 vs. −1.98, P > 0.05), route of administration (SMD: −2.58 vs. −1.89 vs. −2.87, P > 0.05) administration times (SMD: −2.66 vs. −2.18, P > 0.05), or drug dose (SMD: −2.53 vs. −2.35, P > 0.05). However, TMP demonstrated significantly greater efficacy when applied during ischemia compared to reperfusion or continuous administration (SMD: −3.13 vs. −2.05 vs. −1.18, P < 0.05), and this approach also reduced heterogeneity. The anesthesia methods may also be a source of heterogeneity among studies (SMD: 1.71 vs. −2.83 vs. −4.05 vs. −7.31, P < 0.05), but there is still a high degree of heterogeneity and two studies have not reported the anesthesia method yet. Positive results should be treated with caution. Comprehensive details of the subgroup analyses are presented in Table 3.

**TABLE 3:** The results of Subgroup analyses for Scr and BUN.

Comparison	Subgroup	No. of studies	SMD [95% CI]	P for meta-analysis	I2	P for heterogeneity
SCr						
Species	Rat	27	−2.20 [−2.86, −1.53]	<0.00001	85	<0.00001
	Mice	4	−5.00 [−8.15, −1.84]	0.02	89	<0.00001
	Rabbit	1	−1.90 [−3.14, −0.66]	0.003	—	—
Renal IRI model	uIRIx	11	−2.89 [−3.98, −1.81]	<0.00001	84	<0.00001
	Bilateral	16	−2.39 [−3.17, −1.62]	<0.00001	81	<0.00001
	Unilateral	5	−0.87 [−3.16, 1.43]	0.46	90	<0.00001
Anesthesia methods	pentobarbital sodium	19	−1.71 [−2.21, −1.22]	<0.00001	67	<0.0001
	Chloral hydrate	10	−2.83 [−4.69, −0.96]	0.003	91	<0.00001
	Ketamine hydrochloride	1	−4.05 [−5.07, −3.03]	<0.00001	—	—
	NA	2	−7.31 [−13.49, −1.13]	0.02	82	0.02
Duration of ischemia	≤45 min	17	−2.68 [−3.51, −1.85]	<0.00001	82	<0.00001
	>45 min	15	−1.98 [−2.96, −1.00]	<0.0001	87	<0.00001
Application time of TMP	Ischemia	18	−3.13 [−4.25, −2.01]	<0.00001	88	<0.00001
	Reperfusion	9	−2.05 [−3.08, −1.02]	<0.0001	79	<0.00001
	Continuous	5	−1.18 [−1.59, −0.77]	<0.00001	0	0.76
Dose of TMP	≤50 mg/kg	21	−2.53 [−3.22, −1.85]	<0.00001	82	<0.00001
	>50 mg/kg	11	−2.35 [−2.97, −1.72]	0.003	87	<0.00001
Route of administration	IP	19	−2.58 [−3.35, −1.81]	<0.00001	83	<0.00001
	IV	12	−1.89 [−3.08, −0.70]	0.002	87	<0.00001
	Intragastric	1	−2.87 [−4.19, −1.54]	<0.0001	—	—
Administration times	Single	17	−2.66 [−3.79, −1.54]	<0.00001	88	<0.00001
	Multiple	15	−2.18 [−2.84, −1.52]	<0.00001	76	<0.00001
BUN						
Species	Rat	26	−2.35 [−3.02, −1.68]	<0.00001	84	<0.00001
	Mice	4	−2.73 [−4.18, −1.27]	0.0002	70	0.02
renal IRI model	uIRIx	10	−2.55 [−3.60, −1.50]	<0.00001	85	<0.00001
	Bilateral	15	−2.59 [−3.26, −1.91]	<0.00001	71	<0.00001
	Unilateral	5	−1.20 [−3.74, 1.34]	0.36	91	<0.00001
Anesthesia methods	pentobarbital sodium	18	−2.26 [−2.85, −1.68]	<0.00001	72	<0.00001
	Chloral hydrate	10	−1.77 [−3.03, −0.51]	0.006	86	<0.00001
	Ketamine hydrochloride	1	−5.70 [−7.02, −4.38]	<0.00001	—	—
	NA	1	−6.52 [−9.29, −3.74]	<0.00001	—	—
Duration of ischemia	≤45 min	16	−2.44 [−3.00, −1.88]	<0.00001	62	0.0005
	>45 min	14	−2.22 [−3.39, −1.05]	0.0002	90	<0.00001
Application time of TMP	Ischemia	16	−2.42 [−3.40, −1.45]	<0.00001	86	<0.00001
	Reperfusion	9	−3.25 [−4.55, −1.94]	<0.00001	82	<0.00001
	Continuous	5	−1.28 [−1.78, −0.77]	<0.00001	28	0.24
Dose of TMP	≤50 mg/kg	20	−2.84 [−3.52, −2.16]	<0.00001	79	<0.00001
	>50 mg/kg	10	−1.49 [−2.68, −0.30]	0.01	86	<0.00001
Route of administration	IP	19	−2.44 [−3.04, −1.85]	<0.00001	73	<0.00001
	IV	8	−1.41 [−2.94, 0.12]	0.07	87	<0.00001
	Intragastric	1	−2.88 [−4.20, −1.55]	<0.0001	—	—
Administration times	Single	17	−1.98 [−2.78, −1.18]	<0.00001	80	<0.00001
	Multiple	13	−2.96 [−3.92, −2.00]	<0.00001	86	<0.00001

**FIGURE 3:**
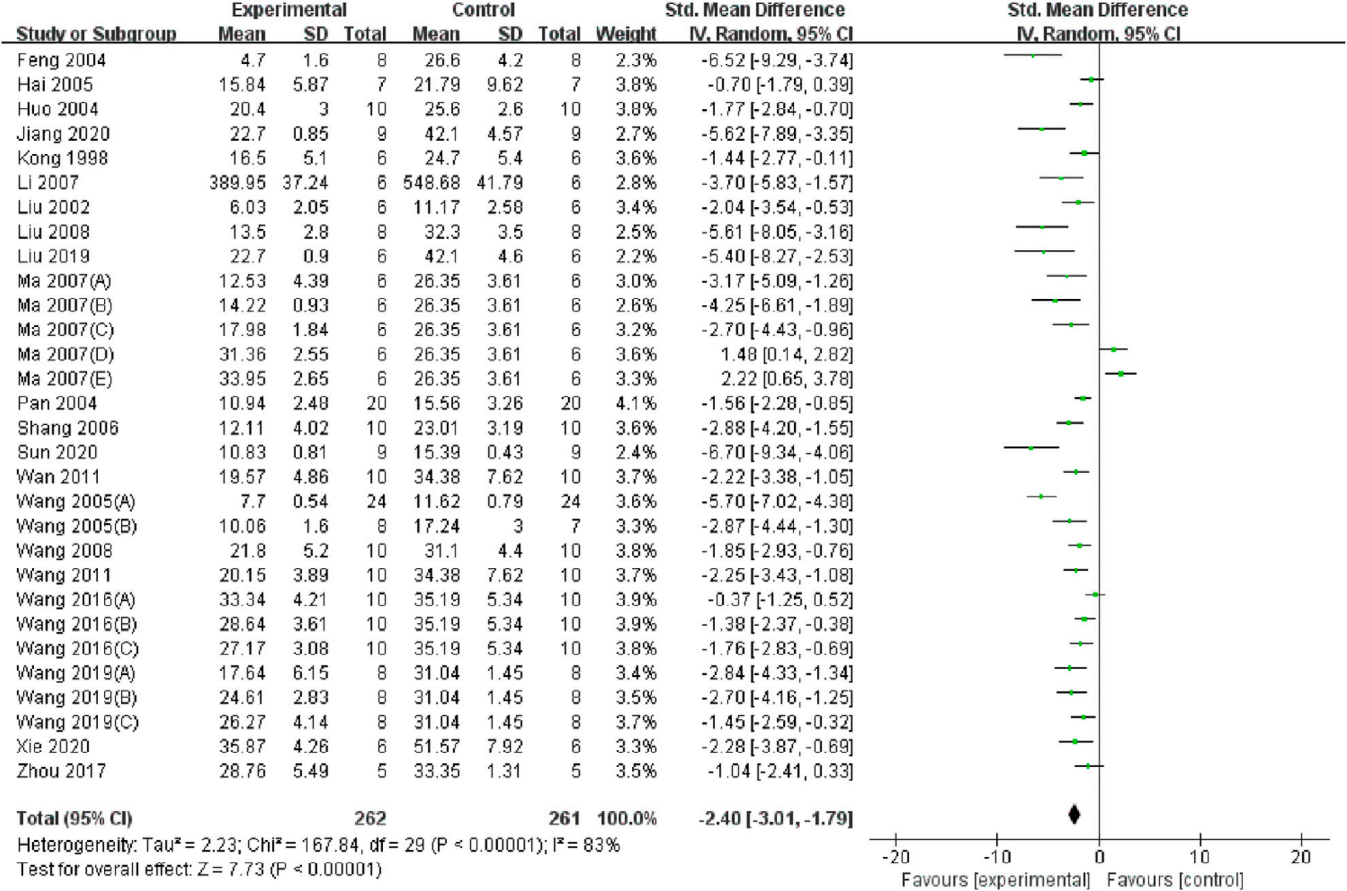
Forest plot showing the pooled effect estimation of TMP on BUN.

Twenty-two studies (Wang et al., 2011; Wang et al., 2019; Wang and Xu, 2016; Li, 2007; Wan et al., 2011; Liu et al., 2008; Ma et al., 2007; Pan et al., 2004; Zhou, 2017; Jiang et al., 2020; Liu, 2019; Hai and Cui, 2005; Wang et al., 2008; Shang et al., 2006; Kong et al., 1998; Wang and Shi, 2005; Sun et al., 2020; Wang et al., 2005; Feng et al., 2004; Liu et al., 2002; Huo et al., 2004; Xie and Lu, 2020) encompassing 30 datasets reported significantly lower BUN levels in the TMP group compared to the control group (SMD = −2.4, 95% CI: −3.01 to −1.79, P < 0.05; heterogeneity: I^2^ = 83%; PQ test < 0.05, Figure 3). The results of subgroup analysis revealed that the application time of TMP (SMD: −2.42 vs. −3.25 vs. −1.28, P < 0.05) and anesthesia methods (SMD: −2.26 vs. −1.77 vs. −5.7, P < 0.05) may be the sources of heterogeneity among studies. However, the effects of TMP were not significant on such species (SMD: −2.35 vs. −2.73, P > 0.05), renal ischemia duration (SMD: −2.44 vs. −2.22, P > 0.05), I/R injury model (SMD: −2.55 vs. −2.59 vs. −1.2., P > 0.05), drug dosage (SMD: −2.84 vs. −1.49, P > 0.05), route of administration (SMD: −2.44 vs. −1.41 vs. −2.88, P > 0.05), or administration times (SMD: −1.98 vs. −2.96, P > 0.05). Details of the subgroup analyses are presented in Table 3.

### 3.5 Overall pooled effect of TMP on oxidative stress, inflammation and apoptosis

**FIGURE 4:**
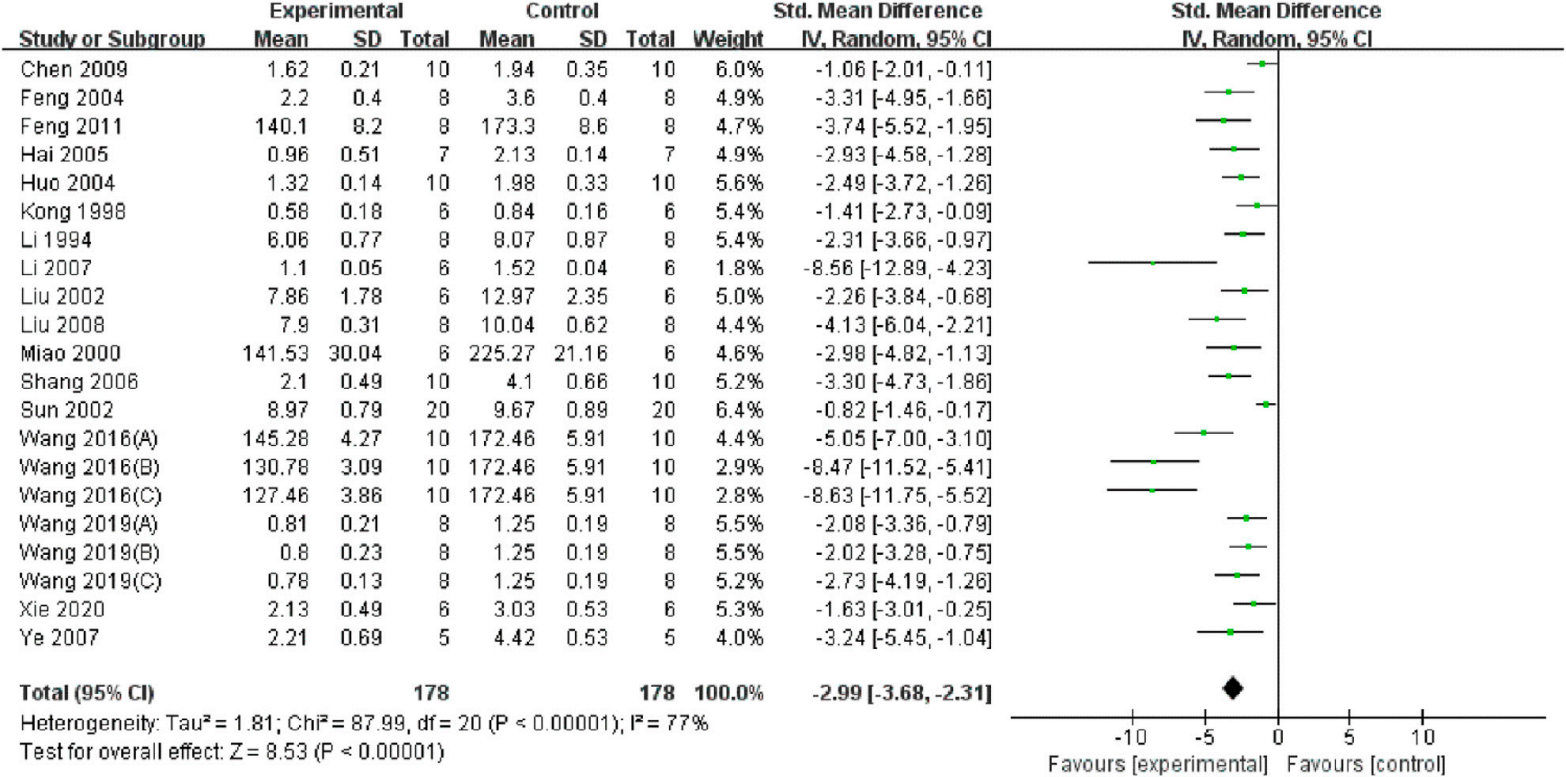
Pooled results of effect of TMP on MDA level.

**FIGURE 5:**
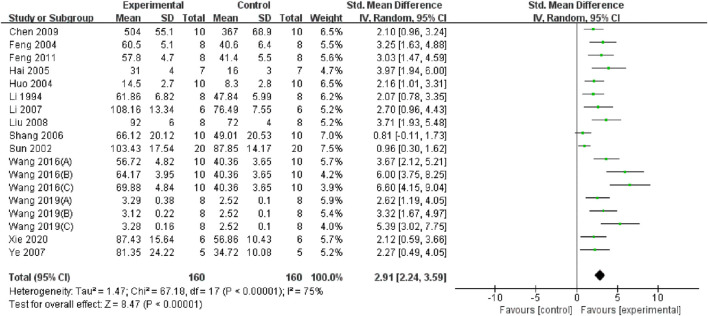
Pooled results of effect of TMP on SOD level.

**FIGURE 6:**
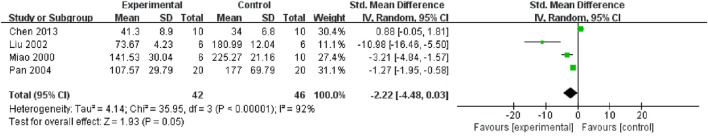
Pooled results of effect of TMP on NO level.

Through a summary analysis of the oxidative stress-related indicators, we observed that compared with the control group, TMP treatment significantly decreased the level of malondialdehyde (MDA) (n = 356; SMD = −2.99, 95% CI: −3.68 to −2.31; P < 0.005; heterogeneity: I^2^ = 77%; PQ test < 0.05, Figure 4). Additionally, TMP treatment significantly increased Superoxide dismutase (SOD) levels (n = 320; SMD = 2.92, 95% CI: 2.25 to 3.60; P < 0.05; heterogeneity: I^2^ = 75%; PQ test < 0.05, Figure 5) in renal tissue. However, no significant effect was observed for renal tissue nitric oxide (NO) levels (n = 88; SMD = −2.22, 95% CI: −4.48 to 0.33; P = 0.05; heterogeneity: I^2^ = 92%; PQ test < 0.05, Figure 6). Two study (Li et al., 1994; Chen et al., 2009) reported that TMP regulates serum MDA and SOD levels, while another highlighted increased levels of glutathione peroxidase (GSHPX) in kidney tissue (Shang et al., 2006). Furthermore, positive effects of TMP were noted on catalase (CAT) and total antioxidant capacity (TAC) by another (Liu et al., 2008).

**FIGURE 7:**
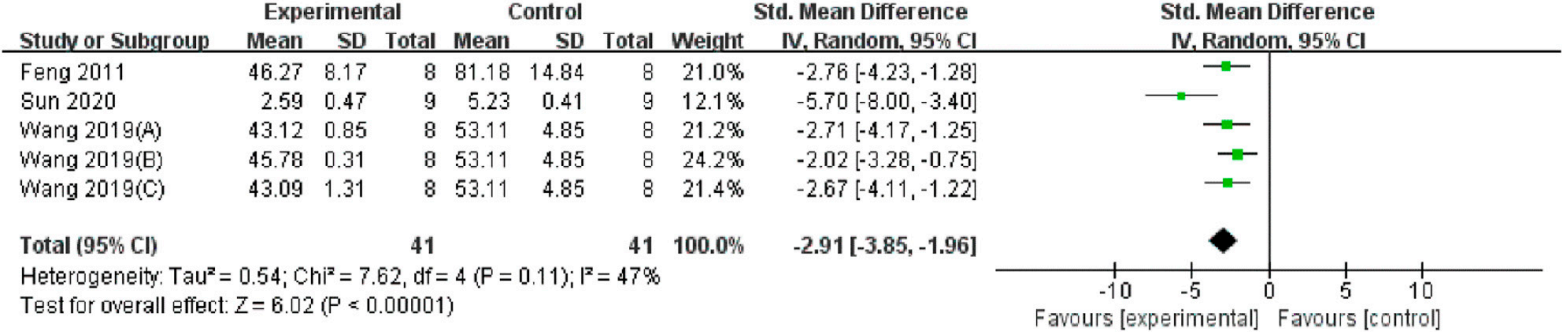
Pooled results of effect of TMP on Serum TNF-α level.

**FIGURE 8:**
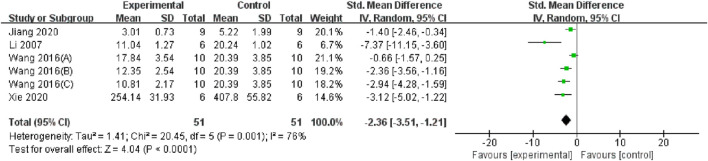
Pooled results of effect of TMP on renal tissue TNF-α level.

**FIGURE 9:**
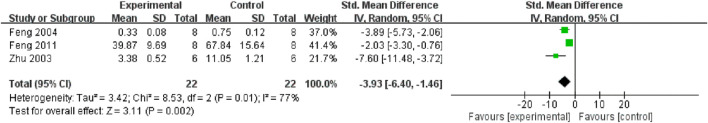
Pooled results of effect of TMP on ICAM-1 level.

Serum and renal tissue TNF-α levels were reported in three (Feng et al., 2011; Wang et al., 2019; Sun et al., 2020) and four studies (Wang and Xu, 2016; Li, 2007; Jiang et al., 2020; Xie and Lu, 2020), respectively. The TMP treatment group demonstrated significantly lower levels compared to the control group (n = 82; SMD = −2.91, 95% CI: −3.85 to −1.96; P < 0.05; heterogeneity: I^2^ = 47%; PQ test = 0.11, Figure 7) and (n = 102; SMD = −2.36, 95% CI: −3.51 to −1.21; P < 0.05; heterogeneity: I^2^ = 76%; PQ test = 0.001, Figure 8). Additionally, three studies (Feng et al., 2011; Zhu et al., 2003; Feng et al., 2004) have reported that TMP treatment reduced the expression of ICAM-1 (n = 44; SMD = −3.93, 95% CI: −6.4 to −1.46; P = 0.002; heterogeneity: I^2^ = 77%; PQ test = 0.01, Figure 9) in renal tissue. Furthermore, two studies (Jiang et al., 2020; Sun et al., 2020) have shown that TMP reduces serum IL-6 levels, while two other studies (Ma et al., 2007; Xie and Lu, 2020) have reported lower IL-10 levels. TMP also reduced the nucleotide-binding oligomerization domain-containing 2 (NOD2) and NLRP3 protein levels (Jiang et al., 2020; Liu, 2019; Sun et al., 2020).

**FIGURE 10:**
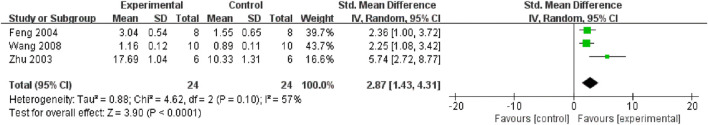
Pooled results of effect of TMP on bcl-2 level.

TMP plays a protective role by alleviating apoptosis in the renal tissue. Three studies (Zhu et al., 2003; Wang et al., 2008; Feng et al., 2004) reported the effect of TMP on the Bcl-2 protein, and the results of the meta-analysis indicated that TMP could upregulate Bcl-2 protein expression (n = 48; SMD = 2.87, 95% CI: 1.43 to 4.31; P < 0.05; heterogeneity: I^2^ = 57%; PQ test = 0.1, Figure 10). Two studies (Wang et al., 2011; Wan et al., 2011) reported the positive effects of TMP on GRP78 and Caspase-12 levels. Furthermore, TMP could regulate the expression of other apoptosis-related proteins, including caspase 3, caspase 3/cleaved caspase 3 and Bax (Jiang et al., 2020; Wang et al., 2008; Sun et al., 2020).

### 3.6 Sensitivity analysis and publication bias

Sensitivity analyses of the main outcome indicators, Scr and BUN, were conducted using a stepwise exclusion method. The analysis revealed no significant differences in the combined effect sizes. After excluding the studies by Ma (2005 E) and Wang 2011, the maximum and minimum effect sizes of Scr were −2.46 (95% CI: −3.06, −1.85) and −2.19 (95% CI: −2.79, −1.59), respectively; Similarly, for BUN, after excluding the studies by Ma (2005 E) and Wang (2005 A) the effect sizes ranged from–2.53 (95% CI: −3.1, −1.95) to −2.24 (95% CI: −2.81, −1.67). These findings indicate that the summary analysis results for Scr and BUN were robust.

**FIGURE 11:**
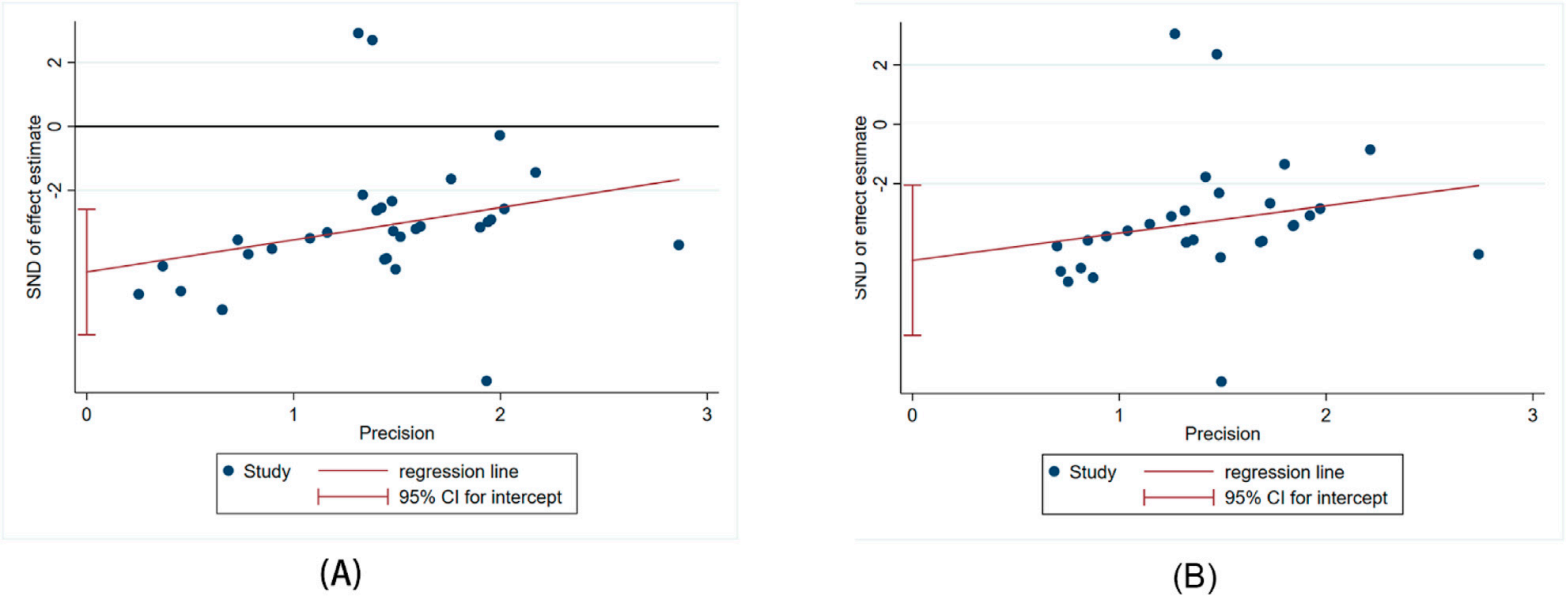
Egger’s publication bias plot for Scr **(A)** and BUN **(B)**.

**FIGURE 12:**
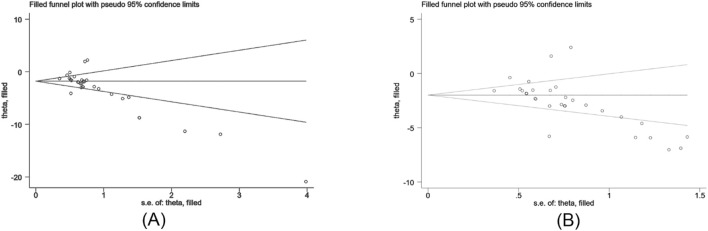
Trim-and-fill analysis for Scr **(A)** and BUN **(B)**.

The Egger’s test was used to assess publication bias for Scr and BUN, and the results indicated publishing bias in both observations (Figure 11A, P > ItI = 0.000 and Figure 11B, P > ItI = 0.001). We assessed the impact of publication bias on the results using the trim-and-fill method (Figures 12A,B). And the results showed there was no supplemental dummy study and no change in the pooled effect of Scr and BUN.

## 4 Discussion

### 4.1 Efficacy of tetramethylpyrazine

This study represents the first preclinical systematic review to evaluate the renoprotective effects of TMP in renal I/R injury. Our meta-analysis revealed that TMP effectively improved renal function markers, including Scr and BUN, while alleviating kidney injury and pathological changes in kidney tissue. These protective effects are likely attributed to the antioxidative, anti-inflammatory, and anti-apoptotic properties of TMP. The results of subgroup analysis in this study showed that the application time of TMP was the source of heterogeneity. In addition, through Egger’s test showed possible publication bias in Scr and BUN, publication bias did not affect the stability of our results through the trim-and-fill method.

Poor research design or insufficient experimental reports may be the key factors leading to high heterogeneity among studies and also the obstacles to the clinical transformation of animal experimental research results. Randomization and blinding are the core set of reporting standards for rigorous study design. Lack of randomization and blinding might lead to overestimation of study results. Meanwhile, the results in such studies being un-interpretable and difficult to reproduce due to inadequate experimental reporting (e.g., lack of randomization, blinding, Data handling). In this meta-analysis and systematic review, most of the literature does not mention the implementation of randomization and blinding, which may introduce potential bias and affect the reliability of the conclusion. Therefore, we recommend that the design, implementation, and reporting of the findings of future preclinical studies should strictly follow the *in vivo* Reporting of Experiments (ARRIVE) (Kilkenny et al., 2012) or Harmonized Reporting of Animal Studies (HARRP) standards (Osborne et al., 2018), which will help researchers improve the quality of animal experiments, increase the reliability of results, and further improve the clinical conversion rate of animal experiment research outcomes.

The rapid increase in Scr and BUN levels is an important sign of renal failure in patients with AKI. Our meta-analysis indicates that TMP significantly improves renal function. Subgroup analysis identified the timing of TMP administration as a major source of heterogeneity in Scr and BUN levels. The timing of drug administration plays a pivotal role in the prognosis of renal I/R injury, and this treatment window can vary significantly between drugs. While some studies have reported that the optimal administration time of TMP for cerebral ischemia-reperfusion injury occurs within 4 h of I/R injury (Zhu et al., 2009), few studies have explored its optimal timing for renal I/R injury. In this analysis, we only extracted the final measurement values for groups with multiple time points to account for variability in study designs and experimental conditions. However, this approach may have overlooked valuable information regarding the time-response relationship of TMP administration. A careful review of the existing literature identified 12 studies (Ye et al., 2007; Li, 2007; Zhu et al., 2003; Zhou, 2017; Miao et al., 2000; Wang and Shi, 2005; Wang et al., 2005; Liu et al., 2002; Huo et al., 2004; Xie and Lu, 2020; Chen et al., 2013; Chen et al., 2009) reporting measured outcomes at multiple time points. Among these, nine studies (Ye et al., 2007; Li, 2007; Zhu et al., 2003; Miao et al., 2000; Wang and Shi, 2005; Wang et al., 2005; Liu et al., 2002; Huo et al., 2004; Xie and Lu, 2020) involved the administration of TMP during ischemia, with seven studies indicating that extended treatment durations does not achieve the desired efficacy in treating renal I/R injury (Ye et al., 2007; Li, 2007; Zhu et al., 2003; Miao et al., 2000; Liu et al., 2002; Huo et al., 2004; Xie and Lu, 2020). And the administration of TMP during reperfusion showed the same results (Zhou, 2017). In addition, two studies (Chen et al., 2013; Chen et al., 2009) examined the continuous use of TMP during both ischemia and reperfusion, finding that its efficacy was greatest at earlier time points. In summary, TMP appears most effective for treating early-stage renal I/R injury. More high-quality studies are needed to determine the optimal TMP administration regimen for the treatment of renal I/R injury and to support its broader clinical application. Future research should also focus on evaluating the efficacy of TMP analogs.

Dose-response relationships play a critical role in evaluating the efficacy of drugs for treating renal I/R injury. Our review of the existing literature revealed that only three studies used dose gradients (Wang et al., 2019; Wang and Xu, 2016; Ma et al., 2007), and among these, only one study (≤50 mg/kg) demonstrated dose-dependent reductions in Scr and BUN levels (Wang and Xu, 2016). Furthermore, Ma et al. observed that the renal pathological results of the high-dose group showed a large amount of tubular epithelial cell necrosis without improving renal function (Ma et al., 2007). The same results were shown in subgroup analyses, in which the pooled analysis effect size showed that lower doses (≤50 mg/kg) were more effective than higher doses, although the dose of TMP was not identified as a source of heterogeneity. These observations indicate that higher doses of TMP fail to deliver the expected therapeutic benefits, potentially due to the low bioavailability (Tsai and Liang, 2001). Pharmacokinetic studies have found that oral TMP has a significant first-pass effect, low water solubility, short biological half-life and low bioavailability. Frequent administration of drugs is required to maintain an effective blood drug concentration. Long-term medication can easily lead to drug accumulation in the body and increase toxic and side effects (Yanyu et al., 2012). In response, many new pharmaceutical advancements, including the structural modification of TMPs, drug delivery methods, and dosage forms, have been explored to improve bioavailability. For example, Nanocarriers (He et al., 2016) have shown the ability to increase the biological half-life and membrane permeability of TMP, thereby prolonging its pharmacological activity. Additionally, microemulsions and fat (Wei et al., 2012) emulsions improved the stability, bioavailability, and tissue distribution of TMP. Transdermal patch administration, such as ethosome (Liu et al., 2011) and pressure-sensitive patches (Qiu et al., 2006), can bypass hepatic and gastrointestinal first-pass metabolism, reduce adverse gastrointestinal reactions to TMP, and improve patient compliance with therapy. Furthermore, due to the complexity of the disease and species-specific metabolism, there may be certain differences in the optimal dosage and frequency of administration between preclinical animal models and human diseases. These factors might limit the clinical transformation of TMP. In response, we suggest that pharmacokinetic studies can be conducted using human data, and adaptive clinical trials can be proposed to determine the efficacy of TMP treatment and the optimal dosage in the future.

In addition, the choice of anesthetic drug also has an impact on the degree of kidney damage and thus interferes with the actual efficacy of the drug. In our systematic review and meta-analysis, the main narcotic drugs were pentobarbital sodium (17%), chloral hydrate (6%), urethane (3%) and ketamine hydrochloride (1%), not mentioned (3%). As we all know, chloral hydrate has poor anesthetic effect, strong irritation, and greater toxic side effects, which may interfere with the experimental results. Intravenous ulatane can cause hemolysis and lead to changes in hemorheology, which may affect the relevant experimental results (Yu et al., 1997). Ketamine is neurotoxic to laboratory animals (Choudhury et al., 2021). However, pentobarbital provides relatively reliable anesthesia for renal IRI surgery in mice (Colin and Daniel, 2024). Therefore, in the experimental research, we should try to choose the anesthesia method which has little influence on the experimental results and is relatively reliable.

### 4.2 Possible mechanisms of action of TMP against renal I/R injury

This systematic review of preclinical studies on the role of TMP in treating renal I/R injury offers valuable insights for future mechanistic research. Possible renal protective mechanisms of TMP include the following: (1) alterations in renal hemodynamics during renal I/R injury lead to a large increase in ROS and downregulation of antioxidant enzyme systems such as catalase (CAT), superoxide dismutase (SOD), and glutathione peroxidase (GPx) (Singh et al., 1993; Johnson and Weinberg, 1993; Qin and Han, 2004). As a potent antioxidant, existing evidence has shown that TMP can reduce MDA levels in renal tissues by regulating NOS activity, inhibiting the overproduction of oxygen free radicals after reperfusion; increasing the activities of SOD, GSHPx, CAT, and TAC in renal tissues; These actions collectively enhances the body’s antioxidant and free radical scavenging ability, thus playing a protective role in the kidney. (2) Interleukin-10 (IL-10) is an important endogenous anti-inflammatory cytokine that inhibits neutrophil activation and plays a significant anti-inflammatory and cytoprotective role (Xie and Lu, 2020). Neutrophil adhesion molecule ICAM-1 and inflammatory factors such as TNF-α, IL-6, and MCP-1 contribute to kidney inflammatory injury (Feng et al., 2011; Jiang et al., 2020). TMP alleviates the inflammatory damage of kidney tissue by down-regulating NOD2-mediated inflammation, inhibiting the expression of NLRP3 protein, reducing the production of cytokines ICAM-1, TNF-α, IL-6 and MCP-1, and increasing the expression of anti-inflammatory cytokine IL-10 (Feng et al., 2011; Jiang et al., 2020; Sun et al., 2020; Feng et al., 2004; Xie and Lu, 2020). (3) Apoptosis of tubular epithelial cells in renal I/R injury is often accompanied by upregulation and activation of GRP78 and Caspase-12 proteins (Wan et al., 2011). TMP ameliorates renal injury by inhibiting the expression of GRP78, Caspase-12, Caspase-3, and caspase 3/cleaved caspase 3 proteins, upregulated bcl-2, and decreased the level of Bax protein (Wang et al., 2011; Wan et al., 2011; Jiang et al., 2020; Wang et al., 2008; Feng et al., 2004).

### 4.3 Limitations

This study had some limitations. First, the overall quality of the included studies was low, with quality assessment scores ranging from 3 to 5, potentially affecting the reliability of the results. Second, the high degree of heterogeneity remains difficult to overlook despite subgroup analyses. There are differences in the modeling methods of different indicators, the anesthesia methods and application time of TMP. This heterogeneity may have reduced the validity of the results. Third, detailed information on the core criteria of study design, such as randomization methods, allocation concealment, and quality control measures, such as consistency of baseline characteristics and blinding of outcome metrics, were not mentioned in the included studies, which may have led to an overestimation of the role of TMP in the included studies, thus affecting the results of the meta-analysis. Therefore, to address these issues and validate our conclusion, high-quality studies with larger sample sizes are needed to confirm our findings.

### 4.4 Conclusion

In conclusion, our meta-analysis indicates that TMP significantly improves renal function and reduces renal injury in the I/R injury model. Its protective effects are closely related to its antioxidant activity, reduction of renal inflammation and apoptosis, and promotion of autophagy. Additionally, the efficacy of TMP for the treatment of ischemic encephalopathy, cardiomyopathy, and chronic kidney disease has been demonstrated in clinical and experimental studies. These results support TMP as a promising candidate for the treatment of renal I/R injury. However, more robust and high-quality experimental designs are needed in future studies to fully elucidate the mechanism of action of TMP and to apply its protective effects in clinical settings. Determining the appropriate dose and application time of TMP should be the focus of future studies.

## Data Availability

The original contributions presented in the study are included in the article/Supplementary Material, further inquiries can be directed to the corresponding author.

## Glossary

AKI: Acute kidney injury
I/R: ischemia-reperfusion
CKD: Chonic kidney disease
ESRD: end-stage renal disease
ICU: intensive care unit
TCM: Traditional Chinese medicine
SMD: standard mean difference
BUN: blood urea nitrogen
GSH-Px: glutathione peroxidase
MCP-1: monocyte chemoattractant protein-1
MDA: malondialdehyde
Scr: serum creatinine
SOD: superoxide dismutase
TNF-α: tumor necrosis factor-α
GSP: glycated serum protein
ROS: reactive oxygen species
NLRP3: NOD-like receptor pyrin domain-containing protein 3
Caspase3: cysteiny-3 aspartate specific protease
Caspase12: cysteiny-12 aspartate specific protease
ICAM-1: intercellular cell adhesion molecule-1
VEGF: vascular endothelial growth factor
Bcl-2: B-cell lymphoma-2
Bax: bcl2-associated x
SOD: superoxide dismutase
IL-10: Interleukin-10
IL-6: Interleukin-6
GRP78: glucose regulating protein78
NOD2: nucleotide-binding oligomerization domain-containing 2
KIM-1: Kidney Injury Molecule-1
XO: xanthine oxidase
ET-1: endothelin-1
NO: nitric oxide
CAT: catalase
TAC: total anti-oxidant capacity
uIRIx: unilateral I/R injury with contralateral nephrectomy
