# Supplementary material for: Tetramethylpyrazine and renal ischemia-reperfusion injury: a systematic review and meta-analysis of preclinical studies
Source: Front Pharmacol. 2025 Jun 2;16:1559314. doi: 10.3389/fphar.2025.1559314 (PMC12171215; doi:10.3389/fphar.2025.1559314)
Supplement: Supplementary file 1 [file Table1.docx]

Supplementary Material

# Supplementary Tables

# 1.1 Supplementary Table 1: Search strategy of included studies in the meta-analysis

# Search strategy for PubMed

| Number | Search terms |
| --- | --- |
| 1 | (kidney [Title/Abstract]) OR (renal [Title/Abstract]) |
| 2 | (((((ischemia/reperfusion [Title/Abstract]) OR (ischemia-reperfusion [Title/Abstract])) OR (ischemia reperfusion [Title/Abstract])) OR (reperfusion [Title/Abstract])) OR (I/R[Title/Abstract])) OR (IRI[Title/Abstract]) |
| 3 | (damage [Title/Abstract]) OR (injury [Title/Abstract]) |
| 4 | ((((((tetramethylpyrazine[Title/Abstract]) OR (chuanxiongzine[Title/Abstract])) OR (ligustrazine[Title/Abstract])) OR (TMPZ[Title/Abstract])) OR (tetramethylpyrazine hydrochloride[Title/Abstract])) OR (Liqustrazine[Title/Abstract])) OR (tetramethyl pyrazine[Title/Abstract]) |
| 5 | 1 AND 2 AND 3 AND 4 |

#

# Search strategy for Embase

| Number | Search terms |
| --- | --- |
| 1 | kidney*: ab,ti OR renal*:ab,ti |
| 2 | 'ischemia reperfusion*': ab,ti OR reperfusion*:ab,ti OR iri*:ab,ti OR 'ischemia/reperfusion*':ab,ti OR 'i/r*':ab,ti |
| 3 | damage*: ab,ti OR injury*:ab,ti |
| 4 | 'tetramethylpyrazine'/exp OR tetramethylpyrazine*: ab,ti OR '2, 3, 5, 6 tetramethylpyrazine*':ab,ti OR ligustizine*:ab,ti OR ligustracin*:ab,ti OR ligustrazine*:ab,ti |
| 5 | 1 AND 2 AND 3 AND 4 |

# Search strategy for Web of science

| Number | Search terms |
| --- | --- |
| 1 | (TS=(kidney)) OR TS=(renal) |
| 2 | (((((TS=(ischemia/reperfusion)) OR TS=(ischemia-reperfusion)) OR TS= (ischemia reperfusion)) OR TS=(reperfusion)) OR TS=(I/R)) OR TS=(IRI) |
| 3 | (TS=(damage)) OR TS=(injury) |
| 4 | ((((((TS=(tetramethylpyrazine)) OR TS=(chuanxiongzine)) OR TS=(ligustrazine)) OR TS=(TMPZ)) OR TS= (tetramethylpyrazine hydrochloride)) OR TS=(Liqustrazine)) OR TS= (tetramethyl pyrazine) |
| 5 | 1 AND 2 AND 3 AND 4 |
