# Supplementary material for: Tetramethylpyrazine and renal ischemia-reperfusion injury: a systematic review and meta-analysis of preclinical studies
Source: Front Pharmacol. 2025 Jun 2;16:1559314. doi: 10.3389/fphar.2025.1559314 (PMC12171215; doi:10.3389/fphar.2025.1559314)

frontiers

Supplementary Material

1 Supplementary Figures

1.1 **Figure S1.** Effects of TMP on serum creatinine (Scr) in animals with Renal ischemia-reperfusion injury (IRI) compared with vehicle control by the subgroup of different (A) species (B) renal IRI model (uIRIx/bilateral/unilateral), (C) anesthetic methods, (D) duration of ischemia, (E) application time of TMP, (F) dose of TMP, (G) route of administration, (H) administration times (single/multiple). Abbreviations: SCr, serum creatinine; CI, confidence interval; IV, inverse variance; SD, standard deviation; iv: intravenous; ip: intraperitoneal.

(A)

Study or Subgroup	Experimental Mean	Experimental SD	Experimental Total	Control Mean	Control SD	Control Total	Weight	Std. Mean Difference IV, Random, 95% CI
**1.2.1 Rat**								
Hai 2005	17.21	7.21	7	23.56	6.35	7	3.7%	-0.88 [-1.99, 0.24]
Jiang 2020	145.6	15.49	9	206.1	25.73	9	3.5%	-2.71 [-4.08, -1.35]
Kong 1998	132.9	35.9	6	185.8	30.6	6	3.5%	-1.46 [-2.80, -0.13]
Li 2007	105.71	7.38	6	167.87	15.53	6	2.4%	-4.72 [-7.29, -2.15]
Liu 2008	58	8	8	236	9	8	0.5%	-19.76 [-27.71, -11.82]
Liu 2019	145.6	15.5	6	206.1	25.7	6	3.2%	-2.63 [-4.34, -0.92]
Ma 2007(A)	50.11	21.88	6	162.61	30.12	6	2.7%	-3.94 [-6.18, -1.71]
Ma 2007(B)	90.2	9.56	6	162.61	30.12	6	3.1%	-2.99 [-4.84, -1.14]
Ma 2007(C)	107.25	31.84	6	162.61	30.12	6	3.5%	-1.65 [-3.04, -0.26]
Ma 2007(D)	211.29	7.37	6	162.61	30.12	6	3.3%	2.05 [0.54, 3.56]
Ma 2007(E)	211.44	18.46	6	162.61	30.12	6	3.4%	1.80 [0.37, 3.24]
Pan 2004	107.57	29.79	20	177	69.79	20	4.0%	-1.27 [-1.95, -0.58]
Shang 2006	14.1	2.03	10	47.21	15.52	10	3.5%	-2.87 [-4.19, -1.54]
Sun 2020	146.4	14.78	9	206.65	25.54	9	3.5%	-2.75 [-4.12, -1.38]
Wan 2011	85.04	12.08	10	218.85	17.9	10	2.1%	-8.39 [-11.42, -5.36]
Wang 2005(A)	87.7	10.54	24	131.62	10.79	24	3.8%	-4.05 [-5.07, -3.03]
Wang 2005(B)	95.14	4.2	8	116.5	13.5	7	3.5%	-2.08 [-3.41, -0.74]
Wang 2008	196	55	10	295	64	10	3.7%	-1.59 [-2.62, -0.55]
Wang 2011	87.23	11.52	10	218.85	17.9	10	2.1%	-8.37 [-11.40, -5.35]
Wang 2016(A)	236.37	54.89	10	276.51	65.47	10	3.8%	-0.64 [-1.54, 0.27]
Wang 2016(B)	202.35	49.43	10	276.51	65.47	10	3.8%	-1.22 [-2.20, -0.25]
Wang 2016(C)	194.24	42.56	10	276.51	65.47	10	3.8%	-1.43 [-2.43, -0.42]
Wang 2019(A)	66.83	40.35	8	147.63	29.89	8	3.5%	-2.15 [-3.45, -0.85]
Wang 2019(B)	101.33	15.79	8	147.63	29.89	8	3.6%	-1.83 [-3.05, -0.61]
Wang 2019(C)	142.7	39.59	8	147.63	29.89	8	3.8%	-0.13 [-1.11, 0.85]
Ye 2007	111.6	47.35	5	356.8	53.43	5	2.3%	-4.39 [-7.15, -1.62]
Zhou 2017	171	59.2	5	244.5	25.9	5	3.4%	-1.45 [-2.94, 0.03]
**Subtotal (95% CI)**			**237**			**236**	**86.9%**	**-2.20 [-2.86, -1.53]**
Heterogeneity: Tau² = 2.40; Chi² = 168.47, df = 26 (P < 0.00001); I² = 85%								
Test for overall effect: Z = 6.47 (P < 0.00001)								
**1.2.2 Mice**								
Feng 2004	32.6	6.5	8	180.6	17.3	8	1.4%	-10.71 [-15.09, -6.32]
Huo 2004	63	10	10	80	12	10	3.8%	-1.47 [-2.49, -0.46]
Liu 2002	73.67	4.23	6	180.99	12.04	6	1.0%	-10.98 [-16.46, -5.50]
Xie 2020	65.33	9.25	6	82.33	8.9	6	3.4%	-1.73 [-3.14, -0.32]
**Subtotal (95% CI)**			**30**			**30**	**9.5%**	**-5.00 [-8.15, -1.84]**
Heterogeneity: Tau² = 7.79; Chi² = 26.62, df = 3 (P < 0.00001); I² = 89%								
Test for overall effect: Z = 3.11 (P = 0.002)								
**1.2.3 Rabbit**								
Li 1994	145.82	64.1	8	276.71	65.84	8	3.6%	-1.90 [-3.14, -0.66]
**Subtotal (95% CI)**			**8**			**8**	**3.6%**	**-1.90 [-3.14, -0.66]**
Heterogeneity: Not applicable								
Test for overall effect: Z = 3.01 (P = 0.003)								
**Total (95% CI)**			**275**			**274**	**100.0%**	**-2.35 [-2.97, -1.72]**
Heterogeneity: Tau² = 2.43; Chi² = 196.12, df = 31 (P < 0.00001); I² = 84%								
Test for overall effect: Z = 7.38 (P < 0.00001)								
Test for subgroup differences: Chi² = 3.23, df = 2 (P = 0.20), I² = 38.1%								

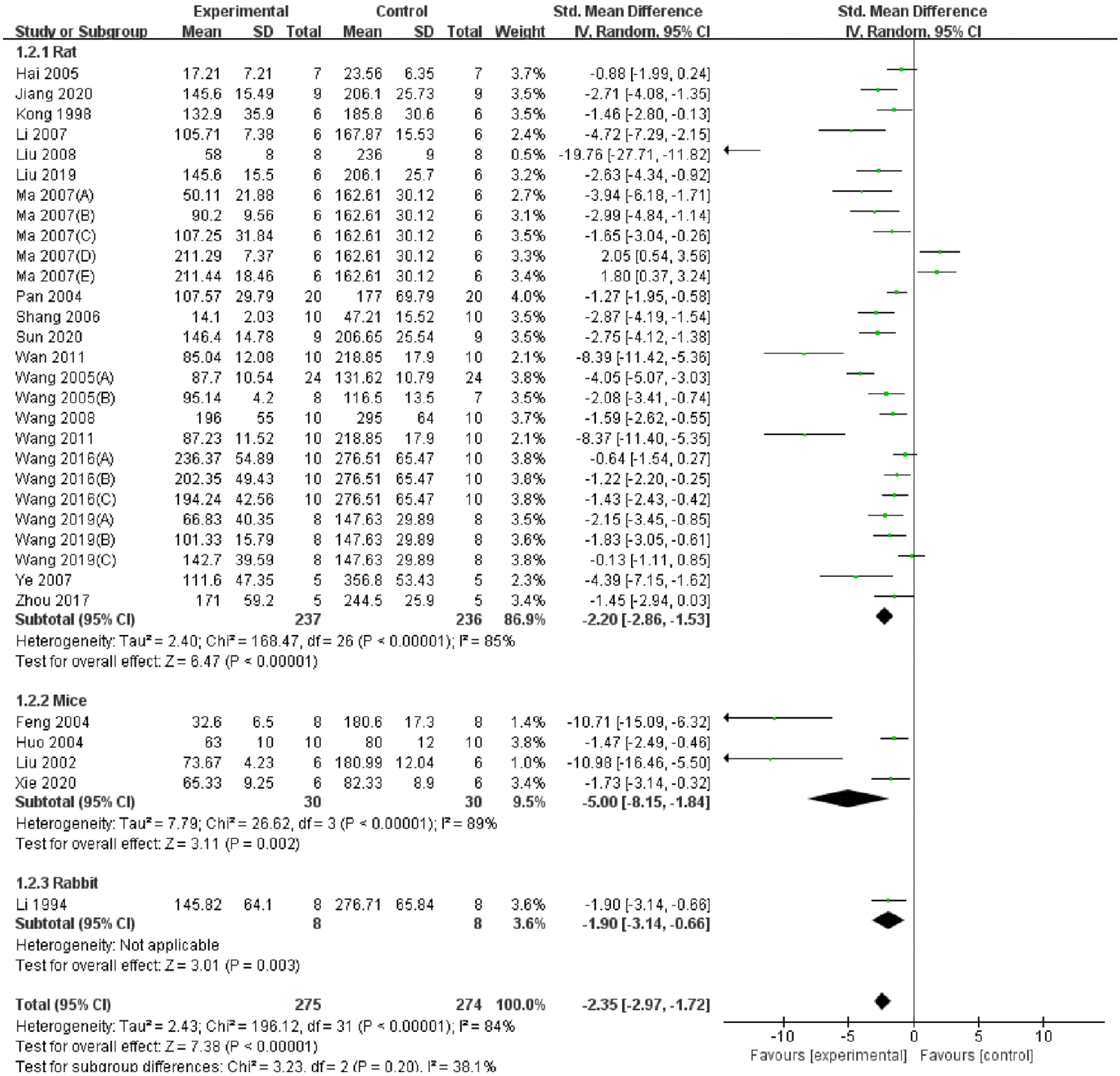

(B)

Study or Subgroup	Experimental Mean	Experimental SD	Experimental Total	Control Mean	Control SD	Control Total	Weight	Std. Mean Difference IV, Random, 95% CI
**1.3.1 ulRlx**								
Feng 2004	32.6	6.5	8	180.6	17.3	8	1.4%	-10.71 [-15.09, -6.32]
Huo 2004	63	10	10	80	12	10	3.8%	-1.47 [-2.49, -0.46]
Kong 1998	132.9	35.9	6	185.8	30.6	6	3.5%	-1.46 [-2.80, -0.13]
Li 2007	105.71	7.38	6	167.87	15.53	6	2.4%	-4.72 [-7.29, -2.15]
Liu 2002	73.67	4.23	6	180.99	12.04	6	1.0%	-10.98 [-16.46, -5.50]
Shang 2006	14.1	2.03	10	47.21	15.52	10	3.5%	-2.87 [-4.19, -1.54]
Wang 2005(A)	87.7	10.54	24	131.62	10.79	24	3.8%	-4.05 [-5.07, -3.03]
Wang 2016(A)	236.37	54.89	10	276.51	65.47	10	3.8%	-0.64 [-1.54, 0.27]
Wang 2016(B)	202.35	49.43	10	276.51	65.47	10	3.8%	-1.22 [-2.20, -0.25]
Wang 2016(C)	194.24	42.56	10	276.51	65.47	10	3.8%	-1.43 [-2.43, -0.42]
Ye 2007	111.6	47.35	5	356.8	53.43	5	2.3%	-4.39 [-7.15, -1.62]
**Subtotal (95% CI)**			**105**			**105**	**33.0%**	**-2.89 [-3.98, -1.81]**
Heterogeneity: Tau² = 2.43; Chi² = 63.97, df = 10 (P < 0.00001); I² = 84%								
Test for overall effect: Z = 5.22 (P < 0.00001)								
**1.3.2 Bilateral**								
Hai 2005	17.21	7.21	7	23.56	6.35	7	3.7%	-0.88 [-1.99, 0.24]
Jiang 2020	145.6	15.49	9	206.1	25.73	9	3.5%	-2.71 [-4.08, -1.35]
Li 1994	145.82	64.1	8	276.71	65.84	8	3.6%	-1.90 [-3.14, -0.66]
Liu 2008	58	8	8	236	9	8	0.5%	-19.76 [-27.71, -11.82]
Liu 2019	145.6	15.5	6	206.1	25.7	6	3.2%	-2.63 [-4.34, -0.92]
Pan 2004	107.57	29.79	20	177	69.79	20	4.0%	-1.27 [-1.95, -0.58]
Sun 2020	146.4	14.78	9	206.65	25.54	9	3.5%	-2.75 [-4.12, -1.38]
Wan 2011	85.04	12.08	10	218.85	17.9	10	2.1%	-8.39 [-11.42, -5.36]
Wang 2005(B)	95.14	4.2	8	116.5	13.5	7	3.5%	-2.08 [-3.41, -0.74]
Wang 2008	196	55	10	295	64	10	3.7%	-1.59 [-2.62, -0.55]
Wang 2011	87.23	11.52	10	218.85	17.9	10	2.1%	-8.37 [-11.40, -5.35]
Wang 2019(A)	66.83	40.35	8	147.63	29.89	8	3.5%	-2.15 [-3.45, -0.85]
Wang 2019(B)	101.33	15.79	8	147.63	29.89	8	3.6%	-1.83 [-3.05, -0.61]
Wang 2019(C)	142.7	39.59	8	147.63	29.89	8	3.8%	-0.13 [-1.11, 0.85]
Xie 2020	65.33	9.25	6	82.33	8.9	6	3.4%	-1.73 [-3.14, -0.32]
Zhou 2017	171	59.2	5	244.5	25.9	5	3.4%	-1.45 [-2.94, 0.03]
**Subtotal (95% CI)**			**140**			**139**	**51.0%**	**-2.39 [-3.17, -1.62]**
Heterogeneity: Tau² = 1.80; Chi² = 77.17, df = 15 (P < 0.00001); I² = 81%								
Test for overall effect: Z = 6.06 (P < 0.00001)								
**1.3.3 Unilateral**								
Ma 2007(A)	50.11	21.88	6	162.61	30.12	6	2.7%	-3.94 [-6.18, -1.71]
Ma 2007(B)	90.2	9.56	6	162.61	30.12	6	3.1%	-2.99 [-4.84, -1.14]
Ma 2007(C)	107.25	31.84	6	162.61	30.12	6	3.5%	-1.65 [-3.04, -0.26]
Ma 2007(D)	211.29	7.37	6	162.61	30.12	6	3.3%	2.05 [0.54, 3.56]
Ma 2007(E)	211.44	18.46	6	162.61	30.12	6	3.4%	1.80 [0.37, 3.24]
**Subtotal (95% CI)**			**30**			**30**	**16.0%**	**-0.87 [-3.16, 1.43]**
Heterogeneity: Tau² = 6.12; Chi² = 39.52, df = 4 (P < 0.00001); I² = 90%								
Test for overall effect: Z = 0.74 (P = 0.46)								
**Total (95% CI)**			**275**			**274**	**100.0%**	**-2.35 [-2.97, -1.72]**
Heterogeneity: Tau² = 2.43; Chi² = 196.12, df = 31 (P < 0.00001); I² = 84%								
Test for overall effect: Z = 7.38 (P < 0.00001)								
Test for subgroup differences: Chi² = 2.48, df = 2 (P = 0.29), I² = 19.3%								

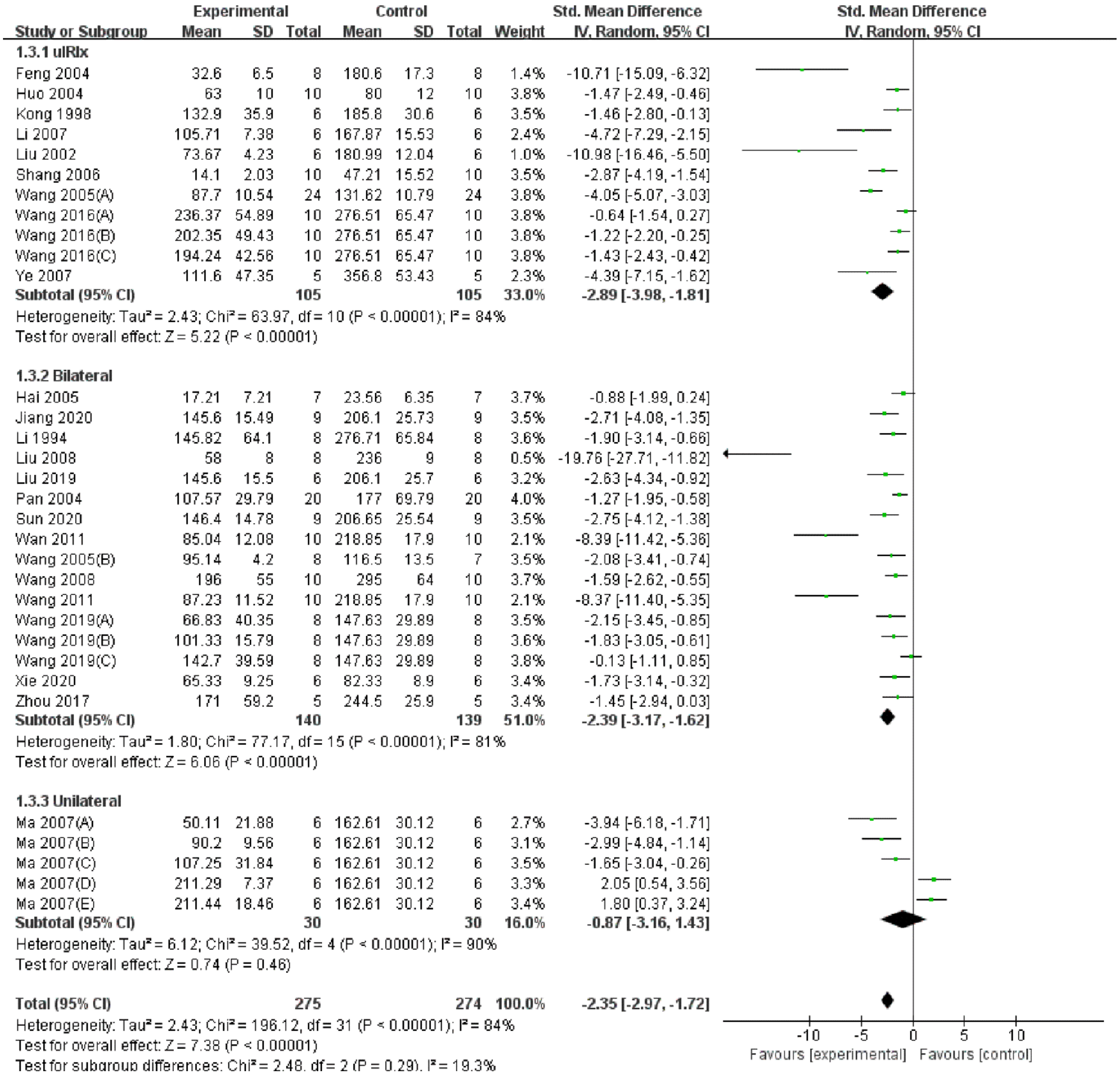

(C)

	Experimental			Control				Std. Mean Difference
**Study or Subgroup**	**Mean**	**SD**	**Total**	**Mean**	**SD**	**Total**	**Weight**	**IV, Random, 95% CI**
**1.4.1 pentobarbital sodium**								
Hai 2005	17.21	7.21	7	23.56	6.35	7	3.7%	-0.88 [-1.99, 0.24]
Huo 2004	63	10	10	80	12	10	3.8%	-1.47 [-2.49, -0.46]
Jiang 2020	145.6	15.49	9	206.1	25.73	9	3.5%	-2.71 [-4.08, -1.35]
Kong 1998	132.9	35.9	6	185.8	30.6	6	3.5%	-1.46 [-2.80, -0.13]
Li 1994	145.82	64.1	8	276.71	65.84	8	3.6%	-1.90 [-3.14, -0.66]
Liu 2002	73.67	4.23	6	180.99	12.04	6	1.0%	-10.98 [-16.46, -5.50]
Liu 2008	58	8	8	236	9	8	0.5%	-19.76 [-27.71, -11.82]
Liu 2019	145.6	15.5	6	206.1	25.7	6	3.2%	-2.63 [-4.34, -0.92]
Pan 2004	107.57	29.79	20	177	69.79	20	4.0%	-1.27 [-1.95, -0.58]
Sun 2020	146.4	14.78	9	206.65	25.54	9	3.5%	-2.75 [-4.12, -1.38]
Wang 2005(B)	95.14	4.2	8	116.5	13.5	7	3.5%	-2.08 [-3.41, -0.74]
Wang 2008	196	55	10	295	64	10	3.7%	-1.59 [-2.62, -0.55]
Wang 2016(A)	236.37	54.89	10	276.51	65.47	10	3.8%	-0.64 [-1.54, 0.27]
Wang 2016(B)	202.35	49.43	10	276.51	65.47	10	3.8%	-1.22 [-2.20, -0.25]
Wang 2016(C)	194.24	42.56	10	276.51	65.47	10	3.8%	-1.43 [-2.43, -0.42]
Wang 2019(A)	66.83	40.35	8	147.63	29.89	8	3.5%	-2.15 [-3.45, -0.85]
Wang 2019(B)	101.33	15.79	8	147.63	29.89	8	3.6%	-1.83 [-3.05, -0.61]
Wang 2019(C)	142.7	39.59	8	147.63	29.89	8	3.8%	-0.13 [-1.11, 0.85]
Xie 2020	65.33	9.25	6	82.33	8.9	6	3.4%	-1.73 [-3.14, -0.32]
**Subtotal (95% CI)**			**167**			**166**	**63.1%**	**-1.71 [-2.21, -1.22]**
Heterogeneity: Tau² = 0.74; Chi² = 55.12, df = 18 (P < 0.0001); I² = 67%								
Test for overall effect: Z = 6.79 (P < 0.00001)								
**1.4.2 Chloral hydrate**								
Li 2007	105.71	7.38	6	167.87	15.53	6	2.4%	-4.72 [-7.29, -2.15]
Ma 2007(A)	50.11	21.88	6	162.61	30.12	6	2.7%	-3.94 [-6.18, -1.71]
Ma 2007(B)	90.2	9.56	6	162.61	30.12	6	3.1%	-2.99 [-4.84, -1.14]
Ma 2007(C)	107.25	31.84	6	162.61	30.12	6	3.5%	-1.65 [-3.04, -0.26]
Ma 2007(D)	211.29	7.37	6	162.61	30.12	6	3.3%	2.05 [0.54, 3.56]
Ma 2007(E)	211.44	18.46	6	162.61	30.12	6	3.4%	1.80 [0.37, 3.24]
Shang 2006	14.1	2.03	10	47.21	15.52	10	3.5%	-2.87 [-4.19, -1.54]
Wan 2011	85.04	12.08	10	218.85	17.9	10	2.1%	-8.39 [-11.42, -5.36]
Wang 2011	87.23	11.52	10	218.85	17.9	10	2.1%	-8.37 [-11.40, -5.35]
Zhou 2017	171	59.2	5	244.5	25.9	5	3.4%	-1.45 [-2.94, 0.03]
**Subtotal (95% CI)**			**71**			**71**	**29.5%**	**-2.83 [-4.69, -0.96]**
Heterogeneity: Tau² = 7.98; Chi² = 98.18, df = 9 (P < 0.00001); I² = 91%								
Test for overall effect: Z = 2.97 (P = 0.003)								
**1.4.3 Ketamine hydrochloride**								
Wang 2005(A)	87.7	10.54	24	131.62	10.79	24	3.8%	-4.05 [-5.07, -3.03]
**Subtotal (95% CI)**			**24**			**24**	**3.8%**	**-4.05 [-5.07, -3.03]**
Heterogeneity: Not applicable								
Test for overall effect: Z = 7.80 (P < 0.00001)								
**1.4.4 NA**								
Feng 2004	32.6	6.5	8	180.6	17.3	8	1.4%	-10.71 [-15.09, -6.32]
Ye 2007	111.6	47.35	5	356.8	53.43	5	2.3%	-4.39 [-7.15, -1.62]
**Subtotal (95% CI)**			**13**			**13**	**3.7%**	**-7.31 [-13.49, -1.13]**
Heterogeneity: Tau² = 16.48; Chi² = 5.71, df = 1 (P = 0.02); I² = 82%								
Test for overall effect: Z = 2.32 (P = 0.02)								
**Total (95% CI)**			**275**			**274**	**100.0%**	**-2.35 [-2.97, -1.72]**
Heterogeneity: Tau² = 2.43; Chi² = 196.12, df = 31 (P < 0.00001); I² = 84%								
Test for overall effect: Z = 7.38 (P < 0.00001)								
Test for subgroup differences: Chi² = 19.50, df = 3 (P = 0.0002), I² = 84.6%								

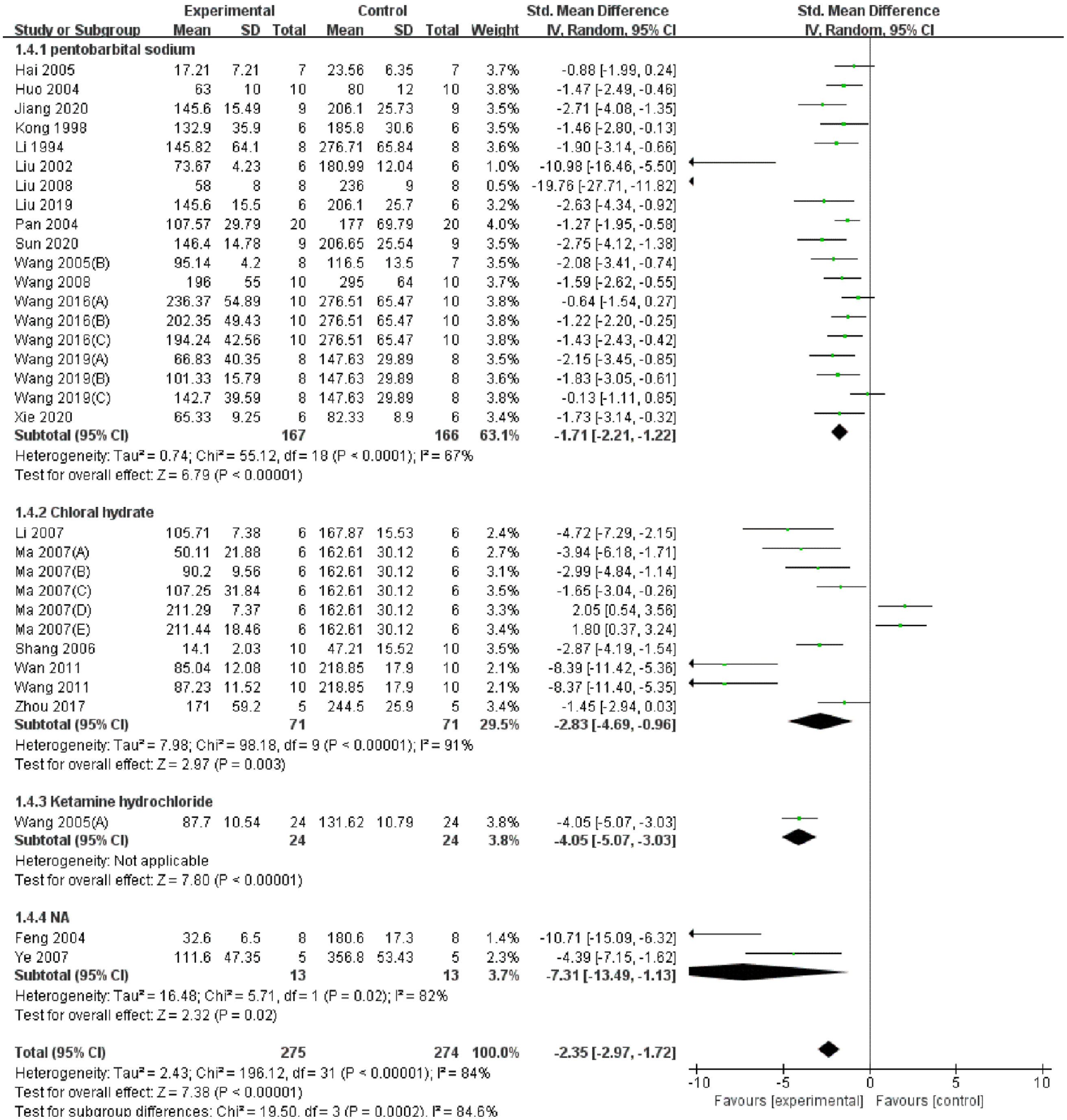

(D)

	Experimental			Control				Std. Mean Difference
Study or Subgroup	Mean	SD	Total	Mean	SD	Total	Weight	IV, Random, 95% CI
**1.5.1 ≤45min**								
Hai 2005	17.21	7.21	7	23.56	6.35	7	3.7%	-0.88 [-1.99, 0.24]
Huo 2004	63	10	10	80	12	10	3.8%	-1.47 [-2.49, -0.46]
Li 1994	145.82	64.1	8	276.71	65.84	8	3.6%	-1.90 [-3.14, -0.66]
Li 2007	105.71	7.38	6	167.87	15.53	6	2.4%	-4.72 [-7.29, -2.15]
Liu 2002	73.67	4.23	6	180.99	12.04	6	1.0%	-10.98 [-16.46, -5.50]
Liu 2008	58	8	8	236	9	8	0.5%	-19.76 [-27.71, -11.82]
Liu 2019	145.6	15.5	6	206.1	25.7	6	3.2%	-2.63 [-4.34, -0.92]
Pan 2004	107.57	29.79	20	177	69.79	20	4.0%	-1.27 [-1.95, -0.58]
Sun 2020	146.4	14.78	9	206.65	25.54	9	3.5%	-2.75 [-4.12, -1.38]
Wan 2011	85.04	12.08	10	218.85	17.9	10	2.1%	-8.39 [-11.42, -5.36]
Wang 2005(B)	95.14	4.2	8	116.5	13.5	7	3.5%	-2.08 [-3.41, -0.74]
Wang 2008	196	55	10	295	64	10	3.7%	-1.59 [-2.62, -0.55]
Wang 2011	87.23	11.52	10	218.85	17.9	10	2.1%	-8.37 [-11.40, -5.35]
Wang 2019(A)	66.83	40.35	8	147.63	29.89	8	3.5%	-2.15 [-3.45, -0.85]
Wang 2019(B)	101.33	15.79	8	147.63	29.89	8	3.6%	-1.83 [-3.05, -0.61]
Wang 2019(C)	142.7	39.59	8	147.63	29.89	8	3.8%	-0.13 [-1.11, 0.85]
Xie 2020	65.33	9.25	6	82.33	8.9	6	3.4%	-1.73 [-3.14, -0.32]
**Subtotal (95% CI)**			**148**			**147**	**51.4%**	**-2.68 [-3.51, -1.85]**
Heterogeneity: Tau^2 = 2.15; Chi^2 = 91.43, df = 16 (P < 0.00001); I^2 = 82%								
Test for overall effect: Z = 6.34 (P < 0.00001)								
**1.5.2 >45min**								
Feng 2004	32.6	6.5	8	180.6	17.3	8	1.4%	-10.71 [-15.09, -6.32]
Jiang 2020	145.6	15.49	9	206.1	25.73	9	3.5%	-2.71 [-4.08, -1.35]
Kong 1998	132.9	35.9	6	185.8	30.6	6	3.5%	-1.46 [-2.80, -0.13]
Ma 2007(A)	50.11	21.88	6	162.61	30.12	6	2.7%	-3.94 [-6.18, -1.71]
Ma 2007(B)	90.2	9.56	6	162.61	30.12	6	3.1%	-2.99 [-4.84, -1.14]
Ma 2007(C)	107.25	31.84	6	162.61	30.12	6	3.5%	-1.65 [-3.04, -0.26]
Ma 2007(D)	211.29	7.37	6	162.61	30.12	6	3.3%	2.05 [0.54, 3.56]
Ma 2007(E)	211.44	18.46	6	162.61	30.12	6	3.4%	1.80 [0.37, 3.24]
Shang 2006	14.1	2.03	10	47.21	15.52	10	3.5%	-2.87 [-4.19, -1.54]
Wang 2005(A)	87.7	10.54	24	131.62	10.79	24	3.8%	-4.05 [-5.07, -3.03]
Wang 2016(A)	236.37	54.89	10	276.51	65.47	10	3.8%	-0.64 [-1.54, 0.27]
Wang 2016(B)	202.35	49.43	10	276.51	65.47	10	3.8%	-1.22 [-2.20, -0.25]
Wang 2016(C)	194.24	42.56	10	276.51	65.47	10	3.8%	-1.43 [-2.43, -0.42]
Ye 2007	111.6	47.35	5	356.8	53.43	5	2.3%	-4.39 [-7.15, -1.62]
Zhou 2017	171	59.2	5	244.5	25.9	5	3.4%	-1.45 [-2.94, 0.03]
**Subtotal (95% CI)**			**127**			**127**	**48.6%**	**-1.98 [-2.96, -1.00]**
Heterogeneity: Tau^2 = 3.05; Chi^2 = 104.43, df = 14 (P < 0.00001); I^2 = 87%								
Test for overall effect: Z = 3.95 (P < 0.0001)								
**Total (95% CI)**			**275**			**274**	**100.0%**	**-2.35 [-2.97, -1.72]**
Heterogeneity: Tau^2 = 2.43; Chi^2 = 196.12, df = 31 (P < 0.00001); I^2 = 84%								
Test for overall effect: Z = 7.38 (P < 0.00001)								
Test for subgroup differences: Chi^2 = 1.15, df = 1 (P = 0.28), I^2 = 12.9%								

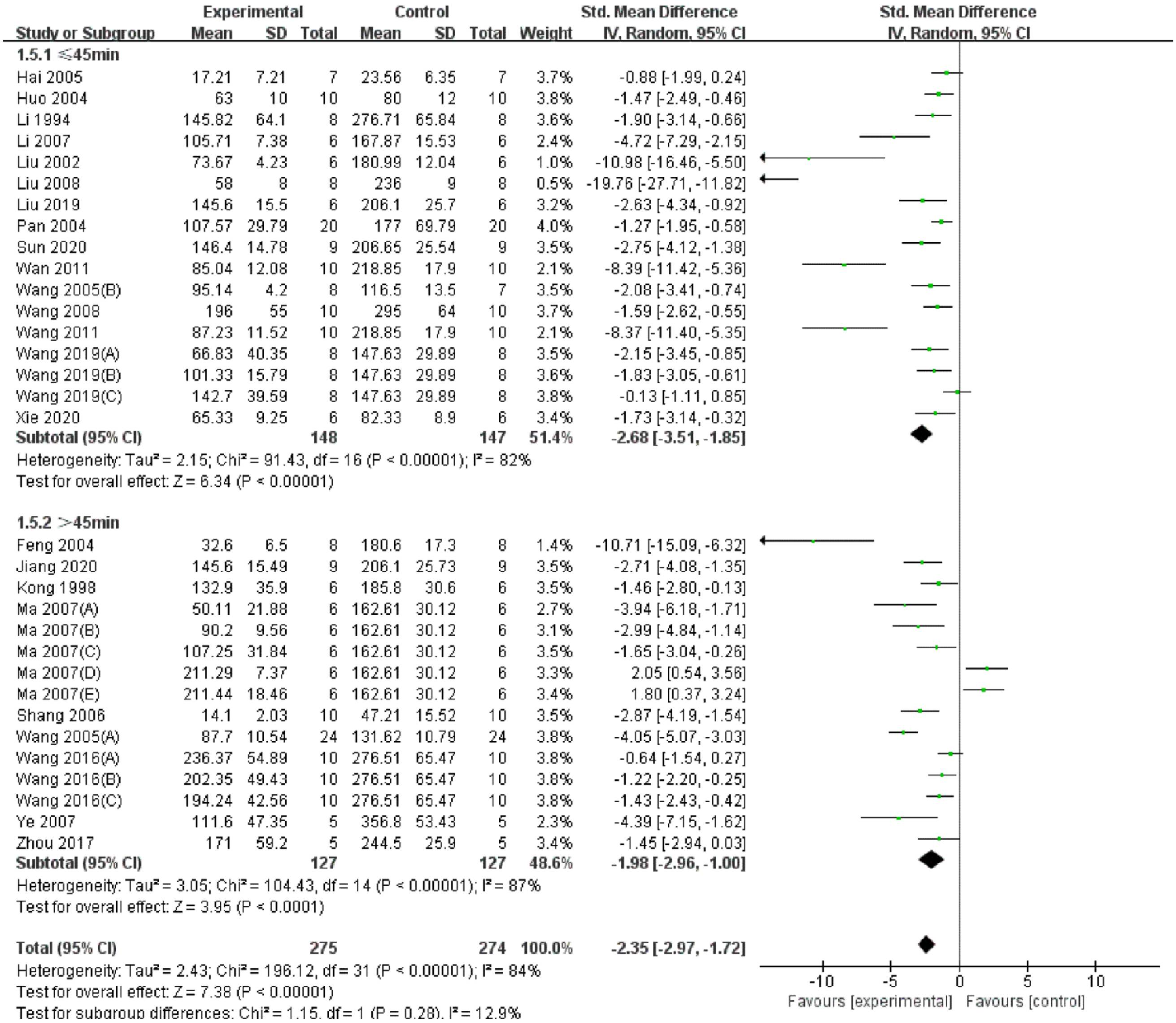

(E)

	Experimental			Control				Std. Mean Difference
Study or Subgroup	Mean	SD	Total	Mean	SD	Total	Weight	IV, Random, 95% CI
**1.6.1 Ischemia**								
Feng 2004	32.6	6.5	8	180.6	17.3	8	1.4%	-10.71 [-15.09, -6.32]
Huo 2004	63	10	10	80	12	10	3.8%	-1.47 [-2.49, -0.46]
Li 1994	145.82	64.1	8	276.71	65.84	8	3.6%	-1.90 [-3.14, -0.66]
Li 2007	105.71	7.38	6	167.87	15.53	6	2.4%	-4.72 [-7.29, -2.15]
Liu 2002	73.67	4.23	6	180.99	12.04	6	1.0%	-10.98 [-16.46, -5.50]
Ma 2007(A)	50.11	21.88	6	162.61	30.12	6	2.7%	-3.94 [-6.18, -1.71]
Ma 2007(B)	90.2	9.56	6	162.61	30.12	6	3.1%	-2.99 [-4.84, -1.14]
Ma 2007(C)	107.25	31.84	6	162.61	30.12	6	3.5%	-1.65 [-3.04, -0.26]
Ma 2007(D)	211.29	7.37	6	162.61	30.12	6	3.3%	2.05 [0.54, 3.56]
Ma 2007(E)	211.44	18.46	6	162.61	30.12	6	3.4%	1.80 [0.37, 3.24]
Shang 2006	14.1	2.03	10	47.21	15.52	10	3.5%	-2.87 [-4.19, -1.54]
Wan 2011	85.04	12.08	10	218.85	17.9	10	2.1%	-8.39 [-11.42, -5.36]
Wang 2005(A)	87.7	10.54	24	131.62	10.79	24	3.8%	-4.05 [-5.07, -3.03]
Wang 2005(B)	95.14	4.2	8	116.5	13.5	7	3.5%	-2.08 [-3.41, -0.74]
Wang 2008	196	55	10	295	64	10	3.7%	-1.59 [-2.62, -0.55]
Wang 2011	87.23	11.52	10	218.85	17.9	10	2.1%	-8.37 [-11.40, -5.35]
Xie 2020	65.33	9.25	6	82.33	8.9	6	3.4%	-1.73 [-3.14, -0.32]
Ye 2007	111.6	47.35	5	356.8	53.43	5	2.3%	-4.39 [-7.15, -1.62]
**Subtotal (95% CI)**			**151**			**150**	**52.6%**	**-3.13 [-4.25, -2.01]**
Heterogeneity: Tau² = 4.70; Chi² = 144.07, df = 17 (P < 0.00001); I² = 88%								
Test for overall effect: Z = 5.49 (P < 0.00001)								
**1.6.2 Reperfusion**								
Hai 2005	17.21	7.21	7	23.56	6.35	7	3.7%	-0.88 [-1.99, 0.24]
Jiang 2020	145.6	15.49	9	206.1	25.73	9	3.5%	-2.71 [-4.08, -1.35]
Liu 2008	58	8	8	236	9	8	0.5%	-19.76 [-27.71, -11.82]
Liu 2019	145.6	15.5	6	206.1	25.7	6	3.2%	-2.63 [-4.34, -0.92]
Sun 2020	146.4	14.78	9	206.65	25.54	9	3.5%	-2.75 [-4.12, -1.38]
Wang 2019(A)	66.83	40.35	8	147.63	29.89	8	3.5%	-2.15 [-3.45, -0.85]
Wang 2019(B)	101.33	15.79	8	147.63	29.89	8	3.6%	-1.83 [-3.05, -0.61]
Wang 2019(C)	142.7	39.59	8	147.63	29.89	8	3.8%	-0.13 [-1.11, 0.85]
Zhou 2017	171	59.2	5	244.5	25.9	5	3.4%	-1.45 [-2.94, 0.03]
**Subtotal (95% CI)**			**68**			**68**	**28.6%**	**-2.05 [-3.08, -1.02]**
Heterogeneity: Tau² = 1.80; Chi² = 37.76, df = 8 (P < 0.00001); I² = 79%								
Test for overall effect: Z = 3.89 (P = 0.0001)								
**1.6.3 Continuous**								
Kong 1998	132.9	35.9	6	185.8	30.6	6	3.5%	-1.46 [-2.80, -0.13]
Pan 2004	107.57	29.79	20	177	69.79	20	4.0%	-1.27 [-1.95, -0.58]
Wang 2016(A)	236.37	54.89	10	276.51	65.47	10	3.8%	-0.64 [-1.54, 0.27]
Wang 2016(B)	202.35	49.43	10	276.51	65.47	10	3.8%	-1.22 [-2.20, -0.25]
Wang 2016(C)	194.24	42.56	10	276.51	65.47	10	3.8%	-1.43 [-2.43, -0.42]
**Subtotal (95% CI)**			**56**			**56**	**18.8%**	**-1.18 [-1.59, -0.77]**
Heterogeneity: Tau² = 0.00; Chi² = 1.87, df = 4 (P = 0.76); I² = 0%								
Test for overall effect: Z = 5.62 (P < 0.00001)								
**Total (95% CI)**			**275**			**274**	**100.0%**	**-2.35 [-2.97, -1.72]**
Heterogeneity: Tau² = 2.43; Chi² = 196.12, df = 31 (P < 0.00001); I² = 84%								
Test for overall effect: Z = 7.38 (P < 0.00001)								
Test for subgroup differences: Chi² = 11.68, df = 2 (P = 0.003), I² = 82.9%								

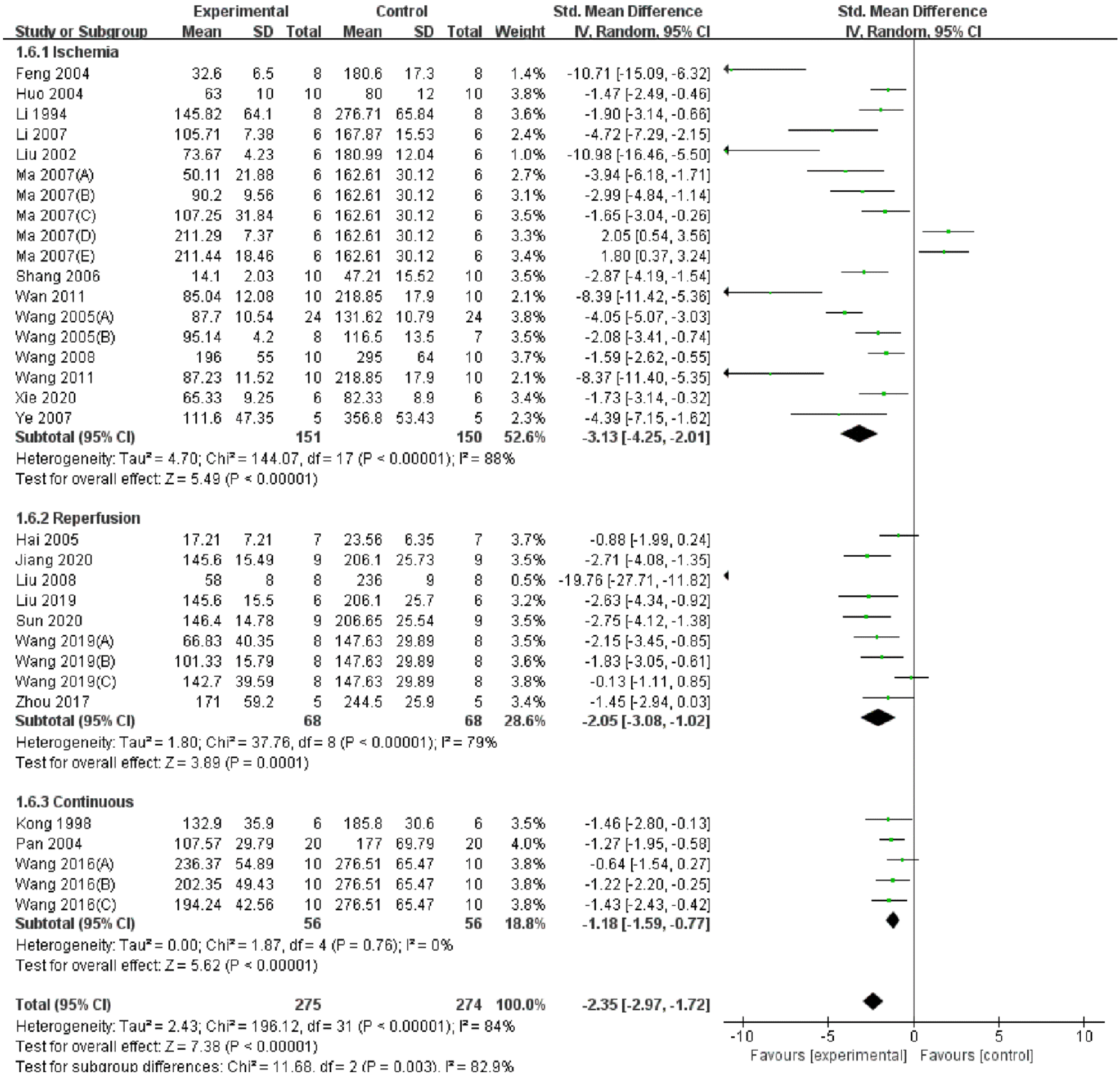

(F)

Study or Subgroup	Experimental Mean	Experimental SD	Experimental Total	Control Mean	Control SD	Control Total	Weight	Std. Mean Difference IV, Random, 95% CI
**1.7.1 ≤50mg/kg**								
Jiang 2020	145.6	15.49	9	206.1	25.73	9	3.5%	-2.71 [-4.08, -1.35]
Kong 1998	132.9	35.9	6	185.8	30.6	6	3.5%	-1.46 [-2.80, -0.13]
Li 1994	145.82	64.1	8	276.71	65.84	8	3.6%	-1.90 [-3.14, -0.66]
Li 2007	105.71	7.38	6	167.87	15.53	6	2.4%	-4.72 [-7.29, -2.15]
Liu 2008	58	8	8	236	9	8	0.5%	-19.76 [-27.71, -11.82]
Liu 2019	145.6	15.5	6	206.1	25.7	6	3.2%	-2.63 [-4.34, -0.92]
Ma 2007(A)	50.11	21.88	6	162.61	30.12	6	2.7%	-3.94 [-6.18, -1.71]
Ma 2007(B)	90.2	9.56	6	162.61	30.12	6	3.1%	-2.99 [-4.84, -1.14]
Ma 2007(C)	107.25	31.84	6	162.61	30.12	6	3.5%	-1.65 [-3.04, -0.26]
Pan 2004	107.57	29.79	20	177	69.79	20	4.0%	-1.27 [-1.95, -0.58]
Sun 2020	146.4	14.78	9	206.65	25.54	9	3.5%	-2.75 [-4.12, -1.38]
Wan 2011	85.04	12.08	10	218.85	17.9	10	2.1%	-8.39 [-11.42, -5.36]
Wang 2005(A)	87.7	10.54	24	131.62	10.79	24	3.8%	-4.05 [-5.07, -3.03]
Wang 2008	196	55	10	295	64	10	3.7%	-1.59 [-2.62, -0.55]
Wang 2011	87.23	11.52	10	218.85	17.9	10	2.1%	-8.37 [-11.40, -5.35]
Wang 2016(A)	236.37	54.89	10	276.51	65.47	10	3.8%	-0.64 [-1.54, 0.27]
Wang 2016(B)	202.35	49.43	10	276.51	65.47	10	3.8%	-1.22 [-2.20, -0.25]
Wang 2016(C)	194.24	42.56	10	276.51	65.47	10	3.8%	-1.43 [-2.43, -0.42]
Wang 2019(A)	66.83	40.35	8	147.63	29.89	8	3.5%	-2.15 [-3.45, -0.85]
Wang 2019(B)	101.33	15.79	8	147.63	29.89	8	3.6%	-1.83 [-3.05, -0.61]
Wang 2019(C)	142.7	39.59	8	147.63	29.89	8	3.8%	-0.13 [-1.11, 0.85]
**Subtotal (95% CI)**			**198**			**198**	**67.3%**	**-2.53 [-3.22, -1.85]**
Heterogeneity: Tau² = 1.89; Chi² = 111.42, df = 20 (P < 0.00001); I² = 82%								
Test for overall effect: Z = 7.23 (P < 0.00001)								
**1.7.2 >50mg/kg**								
Feng 2004	32.6	6.5	8	180.6	17.3	8	1.4%	-10.71 [-15.09, -6.32]
Hai 2005	17.21	7.21	7	23.56	6.35	7	3.7%	-0.88 [-1.99, 0.24]
Huo 2004	63	10	10	80	12	10	3.8%	-1.47 [-2.49, -0.46]
Liu 2002	73.67	4.23	6	180.99	12.04	6	1.0%	-10.98 [-16.46, -5.50]
Ma 2007(D)	211.29	7.37	6	162.61	30.12	6	3.3%	2.05 [0.54, 3.56]
Ma 2007(E)	211.44	18.46	6	162.61	30.12	6	3.4%	1.80 [0.37, 3.24]
Shang 2006	14.1	2.03	10	47.21	15.52	10	3.5%	-2.87 [-4.19, -1.54]
Wang 2005(B)	95.14	4.2	8	116.5	13.5	7	3.5%	-2.08 [-3.41, -0.74]
Xie 2020	65.33	9.25	6	82.33	8.9	6	3.4%	-1.73 [-3.14, -0.32]
Ye 2007	111.6	47.35	5	356.8	53.43	5	2.3%	-4.39 [-7.15, -1.62]
Zhou 2017	171	59.2	5	244.5	25.9	5	3.4%	-1.45 [-2.94, 0.03]
**Subtotal (95% CI)**			**77**			**76**	**32.7%**	**-2.04 [-3.39, -0.69]**
Heterogeneity: Tau² = 4.10; Chi² = 79.04, df = 10 (P < 0.00001); I² = 87%								
Test for overall effect: Z = 2.96 (P = 0.003)								
**Total (95% CI)**			**275**			**274**	**100.0%**	**-2.35 [-2.97, -1.72]**
Heterogeneity: Tau² = 2.43; Chi² = 196.12, df = 31 (P < 0.00001); I² = 84%								
Test for overall effect: Z = 7.38 (P < 0.00001)								
Test for subgroup differences: Chi² = 0.42, df = 1 (P = 0.52), I² = 0%								

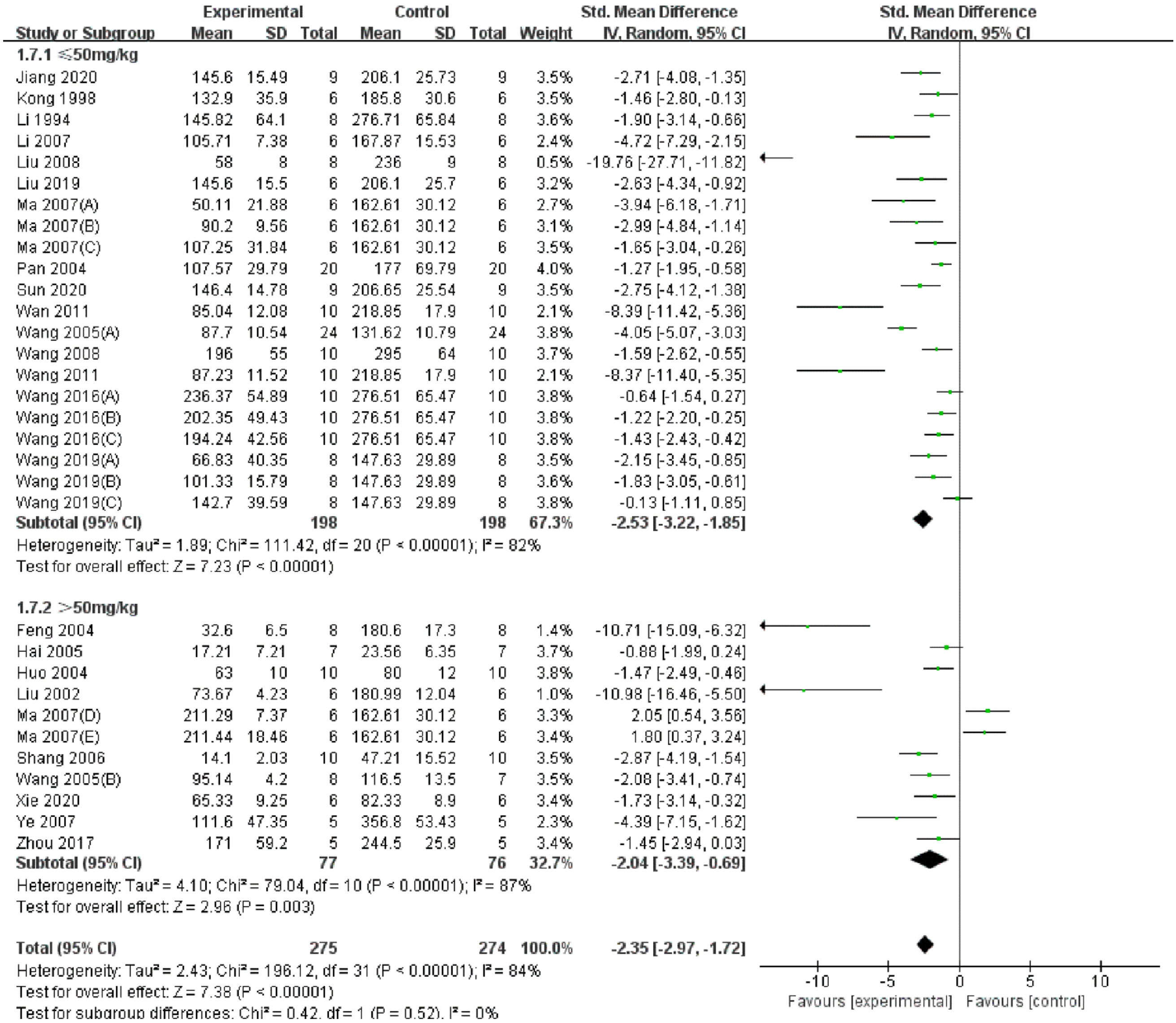

(G)

Study or Subgroup	Experimental Mean	Experimental SD	Experimental Total	Control Mean	Control SD	Control Total	Weight	Std. Mean Difference IV, Random, 95% CI
**1.8.1 IP**								
Feng 2004	32.6	6.5	8	180.6	17.3	8	1.4%	-10.71 [-15.09, -6.32]
Huo 2004	63	10	10	80	12	10	3.8%	-1.47 [-2.49, -0.46]
Jiang 2020	145.6	15.49	9	206.1	25.73	9	3.5%	-2.71 [-4.08, -1.35]
Liu 2002	73.67	4.23	6	180.99	12.04	6	1.0%	-10.98 [-16.46, -5.50]
Liu 2008	58	8	8	236	9	8	0.5%	-19.76 [-27.71, -11.82]
Liu 2019	145.6	15.5	6	206.1	25.7	6	3.2%	-2.63 [-4.34, -0.92]
Pan 2004	107.57	29.79	20	177	69.79	20	4.0%	-1.27 [-1.95, -0.58]
Sun 2020	146.4	14.78	9	206.65	25.54	9	3.5%	-2.75 [-4.12, -1.38]
Wan 2011	85.04	12.08	10	218.85	17.9	10	2.1%	-8.39 [-11.42, -5.36]
Wang 2008	196	55	10	295	64	10	3.7%	-1.59 [-2.62, -0.55]
Wang 2011	87.23	11.52	10	218.85	17.9	10	2.1%	-8.37 [-11.40, -5.35]
Wang 2016(A)	236.37	54.89	10	276.51	65.47	10	3.8%	-0.64 [-1.54, 0.27]
Wang 2016(B)	202.35	49.43	10	276.51	65.47	10	3.8%	-1.22 [-2.20, -0.25]
Wang 2016(C)	194.24	42.56	10	276.51	65.47	10	3.8%	-1.43 [-2.43, -0.42]
Wang 2019(A)	66.83	40.35	8	147.63	29.89	8	3.5%	-2.15 [-3.45, -0.85]
Wang 2019(B)	101.33	15.79	8	147.63	29.89	8	3.6%	-1.83 [-3.05, -0.61]
Wang 2019(C)	142.7	39.59	8	147.63	29.89	8	3.8%	-0.13 [-1.11, 0.85]
Xie 2020	65.33	9.25	6	82.33	8.9	6	3.4%	-1.73 [-3.14, -0.32]
Zhou 2017	171	59.2	5	244.5	25.9	5	3.4%	-1.45 [-2.94, 0.03]
**Subtotal (95% CI)**			**171**			**171**	**57.8%**	**-2.58 [-3.35, -1.81]**
Heterogeneity: Tau² = 2.10; Chi² = 108.51, df = 18 (P < 0.00001); I² = 83%								
Test for overall effect: Z = 6.54 (P < 0.00001)								
**1.8.2 IV**								
Hai 2005	17.21	7.21	7	23.56	6.35	7	3.7%	-0.88 [-1.99, 0.24]
Kong 1998	132.9	35.9	6	185.8	30.6	6	3.5%	-1.46 [-2.80, -0.13]
Li 1994	145.82	64.1	8	276.71	65.84	8	3.6%	-1.90 [-3.14, -0.66]
Li 2007	105.71	7.38	6	167.87	15.53	6	2.4%	-4.72 [-7.29, -2.15]
Ma 2007(A)	50.11	21.88	6	162.61	30.12	6	2.7%	-3.94 [-6.18, -1.71]
Ma 2007(B)	90.2	9.56	6	162.61	30.12	6	3.1%	-2.99 [-4.84, -1.14]
Ma 2007(C)	107.25	31.84	6	162.61	30.12	6	3.5%	-1.65 [-3.04, -0.26]
Ma 2007(D)	211.29	7.37	6	162.61	30.12	6	3.3%	2.05 [0.54, 3.56]
Ma 2007(E)	211.44	18.46	6	162.61	30.12	6	3.4%	1.80 [0.37, 3.24]
Wang 2005(A)	87.7	10.54	24	131.62	10.79	24	3.8%	-4.05 [-5.07, -3.03]
Wang 2005(B)	95.14	4.2	8	116.5	13.5	7	3.5%	-2.08 [-3.41, -0.74]
Ye 2007	111.6	47.35	5	356.8	53.43	5	2.3%	-4.39 [-7.15, -1.62]
**Subtotal (95% CI)**			**94**			**93**	**38.7%**	**-1.89 [-3.08, -0.70]**
Heterogeneity: Tau² = 3.71; Chi² = 84.45, df = 11 (P < 0.00001); I² = 87%								
Test for overall effect: Z = 3.11 (P = 0.002)								
**1.8.3 Intragastric**								
Shang 2006	14.1	2.03	10	47.21	15.52	10	3.5%	-2.87 [-4.19, -1.54]
**Subtotal (95% CI)**			**10**			**10**	**3.5%**	**-2.87 [-4.19, -1.54]**
Heterogeneity: Not applicable								
Test for overall effect: Z = 4.24 (P < 0.0001)								
**Total (95% CI)**			**275**			**274**	**100.0%**	**-2.35 [-2.97, -1.72]**
Heterogeneity: Tau² = 2.43; Chi² = 196.12, df = 31 (P < 0.00001); I² = 84%								
Test for overall effect: Z = 7.38 (P < 0.00001)								
Test for subgroup differences: Chi² = 1.32, df = 2 (P = 0.52), I² = 0%								

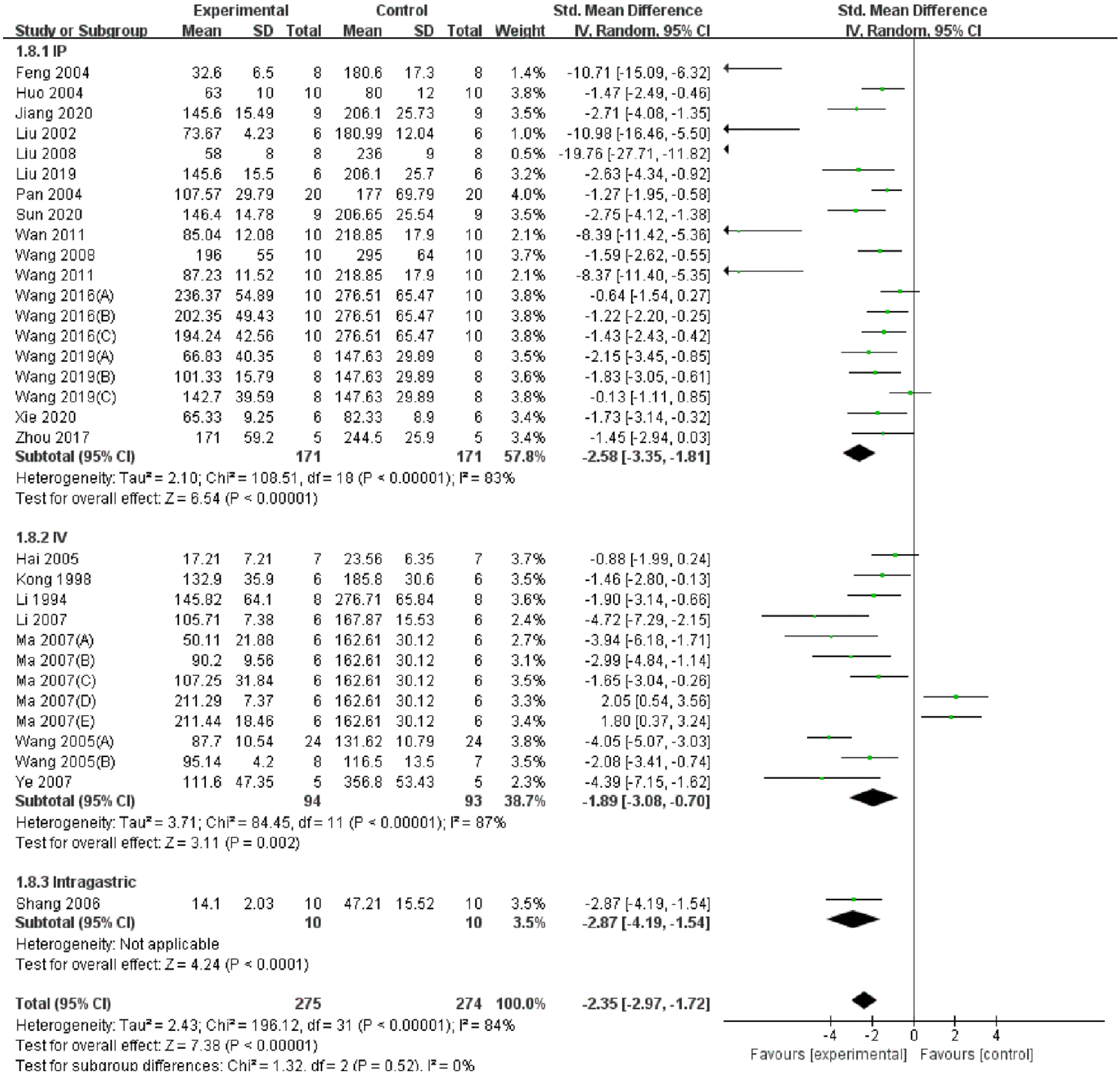

(H)

Study or Subgroup	Experimental Mean	Experimental SD	Experimental Total	Control Mean	Control SD	Control Total	Weight	Std. Mean Difference IV, Random, 95% CI
**1.9.1 Single**								
Feng 2004	32.6	6.5	8	180.6	17.3	8	1.4%	-10.71 [-15.09, -6.32]
Hai 2005	17.21	7.21	7	23.56	6.35	7	3.7%	-0.88 [-1.99, 0.24]
Huo 2004	63	10	10	80	12	10	3.8%	-1.47 [-2.49, -0.46]
Li 2007	105.71	7.38	6	167.87	15.53	6	2.4%	-4.72 [-7.29, -2.15]
Liu 2002	73.67	4.23	6	180.99	12.04	6	1.0%	-10.98 [-16.46, -5.50]
Ma 2007(A)	50.11	21.88	6	162.61	30.12	6	2.7%	-3.94 [-6.18, -1.71]
Ma 2007(B)	90.2	9.56	6	162.61	30.12	6	3.1%	-2.99 [-4.84, -1.14]
Ma 2007(C)	107.25	31.84	6	162.61	30.12	6	3.5%	-1.65 [-3.04, -0.26]
Ma 2007(D)	211.29	7.37	6	162.61	30.12	6	3.3%	2.05 [0.54, 3.56]
Ma 2007(E)	211.44	18.46	6	162.61	30.12	6	3.4%	1.80 [0.37, 3.24]
Wan 2011	85.04	12.08	10	218.85	17.9	10	2.1%	-8.39 [-11.42, -5.36]
Wang 2005(B)	95.14	4.2	8	116.5	13.5	7	3.5%	-2.08 [-3.41, -0.74]
Wang 2011	87.23	11.52	10	218.85	17.9	10	2.1%	-8.37 [-11.40, -5.35]
Wang 2019(A)	66.83	40.35	8	147.63	29.89	8	3.5%	-2.15 [-3.45, -0.85]
Wang 2019(B)	101.33	15.79	8	147.63	29.89	8	3.6%	-1.83 [-3.05, -0.61]
Wang 2019(C)	142.7	39.59	8	147.63	29.89	8	3.8%	-0.13 [-1.11, 0.85]
Zhou 2017	171	59.2	5	244.5	25.9	5	3.4%	-1.45 [-2.94, 0.03]
**Subtotal (95% CI)**			**124**			**123**	**50.2%**	**-2.66 [-3.79, -1.54]**
Heterogeneity: Tau² = 4.48; Chi² = 133.48, df = 16 (P < 0.00001); I² = 88%								
Test for overall effect: Z = 4.64 (P < 0.00001)								
**1.9.2 Multiple**								
Jiang 2020	145.6	15.49	9	206.1	25.73	9	3.5%	-2.71 [-4.08, -1.35]
Kong 1998	132.9	35.9	6	185.8	30.6	6	3.5%	-1.46 [-2.80, -0.13]
Li 1994	145.82	64.1	8	276.71	65.84	8	3.6%	-1.90 [-3.14, -0.66]
Liu 2008	58	8	8	236	9	8	0.5%	-19.76 [-27.71, -11.82]
Liu 2019	145.6	15.5	6	206.1	25.7	6	3.2%	-2.63 [-4.34, -0.92]
Pan 2004	107.57	29.79	20	177	69.79	20	4.0%	-1.27 [-1.95, -0.58]
Shang 2006	14.1	2.03	10	47.21	15.52	10	3.5%	-2.87 [-4.19, -1.54]
Sun 2020	146.4	14.78	9	206.65	25.54	9	3.5%	-2.75 [-4.12, -1.38]
Wang 2005(A)	87.7	10.54	24	131.62	10.79	24	3.8%	-4.05 [-5.07, -3.03]
Wang 2008	196	55	10	295	64	10	3.7%	-1.59 [-2.62, -0.55]
Wang 2016(A)	236.37	54.89	10	276.51	65.47	10	3.8%	-0.64 [-1.54, 0.27]
Wang 2016(B)	202.35	49.43	10	276.51	65.47	10	3.8%	-1.22 [-2.20, -0.25]
Wang 2016(C)	194.24	42.56	10	276.51	65.47	10	3.8%	-1.43 [-2.43, -0.42]
Xie 2020	65.33	9.25	6	82.33	8.9	6	3.4%	-1.73 [-3.14, -0.32]
Ye 2007	111.6	47.35	5	356.8	53.43	5	2.3%	-4.39 [-7.15, -1.62]
**Subtotal (95% CI)**			**151**			**151**	**49.8%**	**-2.18 [-2.84, -1.52]**
Heterogeneity: Tau² = 1.18; Chi² = 59.51, df = 14 (P < 0.00001); I² = 76%								
Test for overall effect: Z = 6.47 (P < 0.00001)								
**Total (95% CI)**			**275**			**274**	**100.0%**	**-2.35 [-2.97, -1.72]**
Heterogeneity: Tau² = 2.43; Chi² = 196.12, df = 31 (P < 0.00001); I² = 84%								
Test for overall effect: Z = 7.38 (P < 0.00001)								
Test for subgroup differences: Chi² = 0.52, df = 1 (P = 0.47), I² = 0%								

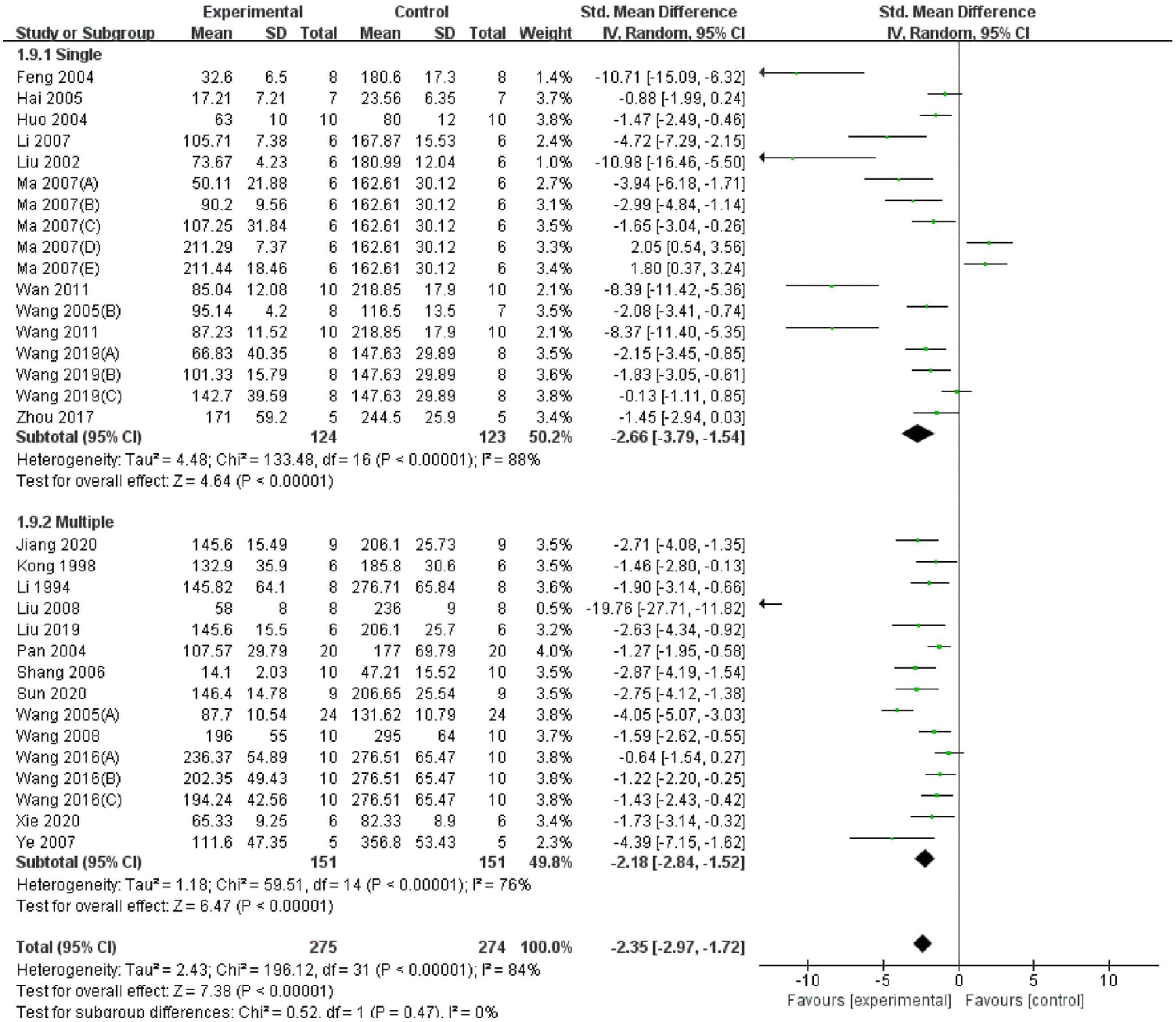

1.2 Figure S2. Effects of ligustrazine on blood urea nitrogen (BUN) in animals with Renal ischemia-reperfusion injury (IRI) compared with vehicle control by the subgroup of different (A) species (B) renal IRI model (uIRIx/bilateral/unilateral), (C) anesthetic methods, (D) duration of ischemia, (E) application time of TMP, (F) dose of TMP, (G) route of administration, (H) administration times (single/multiple). Abbreviations: BUN, blood urea nitrogen; CI, confidence interval; IV, inverse variance; SD, standard deviation. iv: intravenous; ip: intraperitoneal.

(A)

	Experimental			Control				Std. Mean Difference
Study or Subgroup	Mean	SD	Total	Mean	SD	Total	Weight	IV, Random, 95% CI
**5.2.1 Rat**								
Hai 2005	15.84	5.87	7	21.79	9.62	7	3.8%	-0.70 [-1.79, 0.39]
Jiang 2020	22.7	0.85	9	42.1	4.57	9	2.7%	-5.62 [-7.89, -3.35]
Kong 1998	16.5	5.1	6	24.7	5.4	6	3.6%	-1.44 [-2.77, -0.11]
Li 2007	389.95	37.24	6	548.68	41.79	6	2.8%	-3.70 [-5.83, -1.57]
Liu 2008	13.5	2.8	8	32.3	3.5	8	2.5%	-5.61 [-8.05, -3.16]
Liu 2019	22.7	0.9	6	42.1	4.6	6	2.2%	-5.40 [-8.27, -2.53]
Ma 2007(A)	12.53	4.39	6	26.35	3.61	6	3.0%	-3.17 [-5.09, -1.26]
Ma 2007(B)	14.22	0.93	6	26.35	3.61	6	2.6%	-4.25 [-6.61, -1.89]
Ma 2007(C)	17.98	1.84	6	26.35	3.61	6	3.2%	-2.70 [-4.43, -0.96]
Ma 2007(D)	31.36	2.55	6	26.35	3.61	6	3.6%	1.48 [0.14, 2.82]
Ma 2007(E)	33.95	2.65	6	26.35	3.61	6	3.3%	2.22 [0.65, 3.78]
Pan 2004	10.94	2.48	20	15.56	3.26	20	4.1%	-1.56 [-2.28, -0.85]
Shang 2006	12.11	4.02	10	23.01	3.19	10	3.6%	-2.88 [-4.20, -1.55]
Sun 2020	10.83	0.81	9	15.39	0.43	9	2.4%	-6.70 [-9.34, -4.06]
Wan 2011	19.57	4.86	10	34.38	7.62	10	3.7%	-2.22 [-3.38, -1.05]
Wang 2005(A)	7.7	0.54	24	11.62	0.79	24	3.6%	-5.70 [-7.02, -4.38]
Wang 2005(B)	10.06	1.6	8	17.24	3	7	3.3%	-2.87 [-4.44, -1.30]
Wang 2008	21.8	5.2	10	31.1	4.4	10	3.8%	-1.85 [-2.93, -0.76]
Wang 2011	20.15	3.89	10	34.38	7.62	10	3.7%	-2.25 [-3.43, -1.08]
Wang 2016(A)	33.34	4.21	10	35.19	5.34	10	3.9%	-0.37 [-1.25, 0.52]
Wang 2016(B)	28.64	3.61	10	35.19	5.34	10	3.9%	-1.38 [-2.37, -0.38]
Wang 2016(C)	27.17	3.08	10	35.19	5.34	10	3.8%	-1.76 [-2.83, -0.69]
Wang 2019(A)	17.64	6.15	8	31.04	1.45	8	3.4%	-2.84 [-4.33, -1.34]
Wang 2019(B)	24.61	2.83	8	31.04	1.45	8	3.5%	-2.70 [-4.16, -1.25]
Wang 2019(C)	26.27	4.14	8	31.04	1.45	8	3.7%	-1.45 [-2.59, -0.32]
Zhou 2017	28.76	5.49	5	33.35	1.31	5	3.5%	-1.04 [-2.41, 0.33]
**Subtotal (95% CI)**			**232**			**231**	**87.2%**	**-2.35 [-3.02, -1.68]**
Heterogeneity: Tau² = 2.40; Chi² = 156.85, df = 25 (P < 0.00001); I² = 84%								
Test for overall effect: Z = 6.87 (P < 0.00001)								
**5.2.2 Mice**								
Feng 2004	4.7	1.6	8	26.6	4.2	8	2.3%	-6.52 [-9.29, -3.74]
Huo 2004	20.4	3	10	25.6	2.6	10	3.8%	-1.77 [-2.84, -0.70]
Liu 2002	6.03	2.05	6	11.17	2.58	6	3.4%	-2.04 [-3.54, -0.53]
Xie 2020	35.87	4.26	6	51.57	7.92	6	3.3%	-2.28 [-3.87, -0.69]
**Subtotal (95% CI)**			**30**			**30**	**12.8%**	**-2.73 [-4.18, -1.27]**
Heterogeneity: Tau² = 1.46; Chi² = 9.88, df = 3 (P = 0.02); I² = 70%								
Test for overall effect: Z = 3.68 (P = 0.0002)								
**Total (95% CI)**			**262**			**261**	**100.0%**	**-2.40 [-3.01, -1.79]**
Heterogeneity: Tau² = 2.23; Chi² = 167.84, df = 29 (P < 0.00001); I² = 83%								
Test for overall effect: Z = 7.73 (P < 0.00001)								
Test for subgroup differences: Chi² = 0.22, df = 1 (P = 0.64), I² = 0%								

Std. Mean Difference
IV, Random, 95% CI

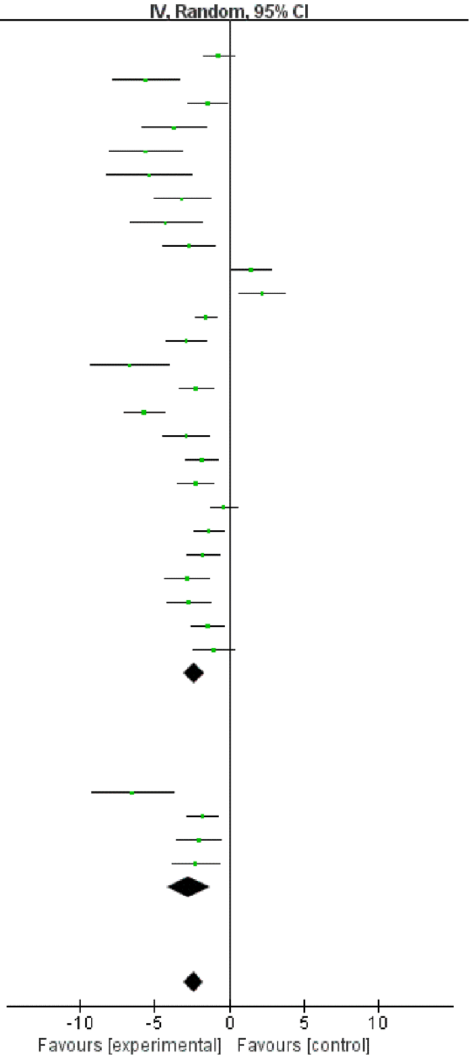

(B)

Study or Subgroup	Experimental Mean	Experimental SD	Experimental Total	Control Mean	Control SD	Control Total	Weight	Std. Mean Difference IV, Random, 95% CI
**5.3.1 ulRIx**								
Feng 2004	4.7	1.6	8	26.6	4.2	8	2.4%	-6.52 [-9.29, -3.74]
Huo 2004	20.4	3	10	25.6	2.6	10	4.7%	-1.77 [-2.84, -0.70]
Kong 1998	16.5	5.1	6	24.7	5.4	6	4.3%	-1.44 [-2.77, -0.11]
Li 2007	389.95	37.24	6	548.68	41.79	6	3.1%	-3.70 [-5.83, -1.57]
Liu 2002	6.03	2.05	6	11.17	2.58	6	4.0%	-2.04 [-3.54, -0.53]
Shang 2006	12.11	4.02	10	23.01	3.19	10	4.3%	-2.88 [-4.20, -1.55]
Wang 2005(A)	7.7	0.54	24	11.62	0.79	24	4.3%	-5.70 [-7.02, -4.38]
Wang 2016(A)	33.34	4.21	10	35.19	5.34	10	4.9%	-0.37 [-1.25, 0.52]
Wang 2016(B)	28.64	3.61	10	35.19	5.34	10	4.8%	-1.38 [-2.37, -0.38]
Wang 2016(C)	27.17	3.08	10	35.19	5.34	10	4.7%	-1.76 [-2.83, -0.69]
**Subtotal (95% CI)**			**100**			**100**	**41.4%**	**-2.55 [-3.60, -1.50]**
Heterogeneity: Tau² = 2.33; Chi² = 60.10, df = 9 (P < 0.00001); I² = 85%								
Test for overall effect: Z = 4.76 (P < 0.00001)								
**5.3.2 Bilateral**								
Hai 2005	15.84	5.87	7	21.79	9.62	7	4.6%	-0.70 [-1.79, 0.39]
Jiang 2020	22.7	0.85	9	42.1	4.57	9	3.0%	-5.62 [-7.89, -3.35]
Liu 2008	13.5	2.8	8	32.3	3.5	8	2.8%	-5.61 [-8.05, -3.16]
Liu 2019	22.7	0.9	6	42.1	4.6	6	2.3%	-5.40 [-8.27, -2.53]
Pan 2004	10.94	2.48	20	15.56	3.26	20	5.1%	-1.56 [-2.28, -0.85]
Sun 2020	10.83	0.81	9	15.39	0.43	9	2.5%	-6.70 [-9.34, -4.06]
Wan 2011	19.57	4.86	10	34.38	7.62	10	4.5%	-2.22 [-3.38, -1.05]
Wang 2005(B)	10.06	1.6	8	17.24	3	7	3.9%	-2.87 [-4.44, -1.30]
Wang 2008	21.8	5.2	10	31.1	4.4	10	4.6%	-1.85 [-2.93, -0.76]
Wang 2011	20.15	3.89	10	34.38	7.62	10	4.5%	-2.25 [-3.43, -1.08]
Wang 2019(A)	17.64	6.15	8	31.04	1.45	8	4.0%	-2.84 [-4.33, -1.34]
Wang 2019(B)	24.61	2.83	8	31.04	1.45	8	4.1%	-2.70 [-4.16, -1.25]
Wang 2019(C)	26.27	4.14	8	31.04	1.45	8	4.6%	-1.45 [-2.59, -0.32]
Xie 2020	35.87	4.26	6	51.57	7.92	6	3.9%	-2.28 [-3.87, -0.69]
Zhou 2017	28.76	5.49	5	33.35	1.31	5	4.2%	-1.04 [-2.41, 0.33]
**Subtotal (95% CI)**			**132**			**131**	**58.6%**	**-2.59 [-3.26, -1.91]**
Heterogeneity: Tau² = 1.16; Chi² = 48.75, df = 14 (P < 0.00001); I² = 71%								
Test for overall effect: Z = 7.51 (P < 0.00001)								
**5.3.3 Unilateral**								
Ma 2007(A)	0	0	0	0	0	0		Not estimable
Ma 2007(B)	0	0	0	0	0	0		Not estimable
Ma 2007(C)	0	0	0	0	0	0		Not estimable
Ma 2007(D)	0	0	0	0	0	0		Not estimable
Ma 2007(E)	0	0	0	0	0	0		Not estimable
**Subtotal (95% CI)**			**0**			**0**		**Not estimable**
Heterogeneity: Not applicable								
Test for overall effect: Not applicable								
**Total (95% CI)**			**232**			**231**	**100.0%**	**-2.58 [-3.16, -2.01]**
Heterogeneity: Tau² = 1.54; Chi² = 108.93, df = 24 (P < 0.00001); I² = 78%								
Test for overall effect: Z = 8.85 (P < 0.00001)								
Test for subgroup differences: Chi² = 0.00, df = 1 (P = 0.96), I² = 0%								

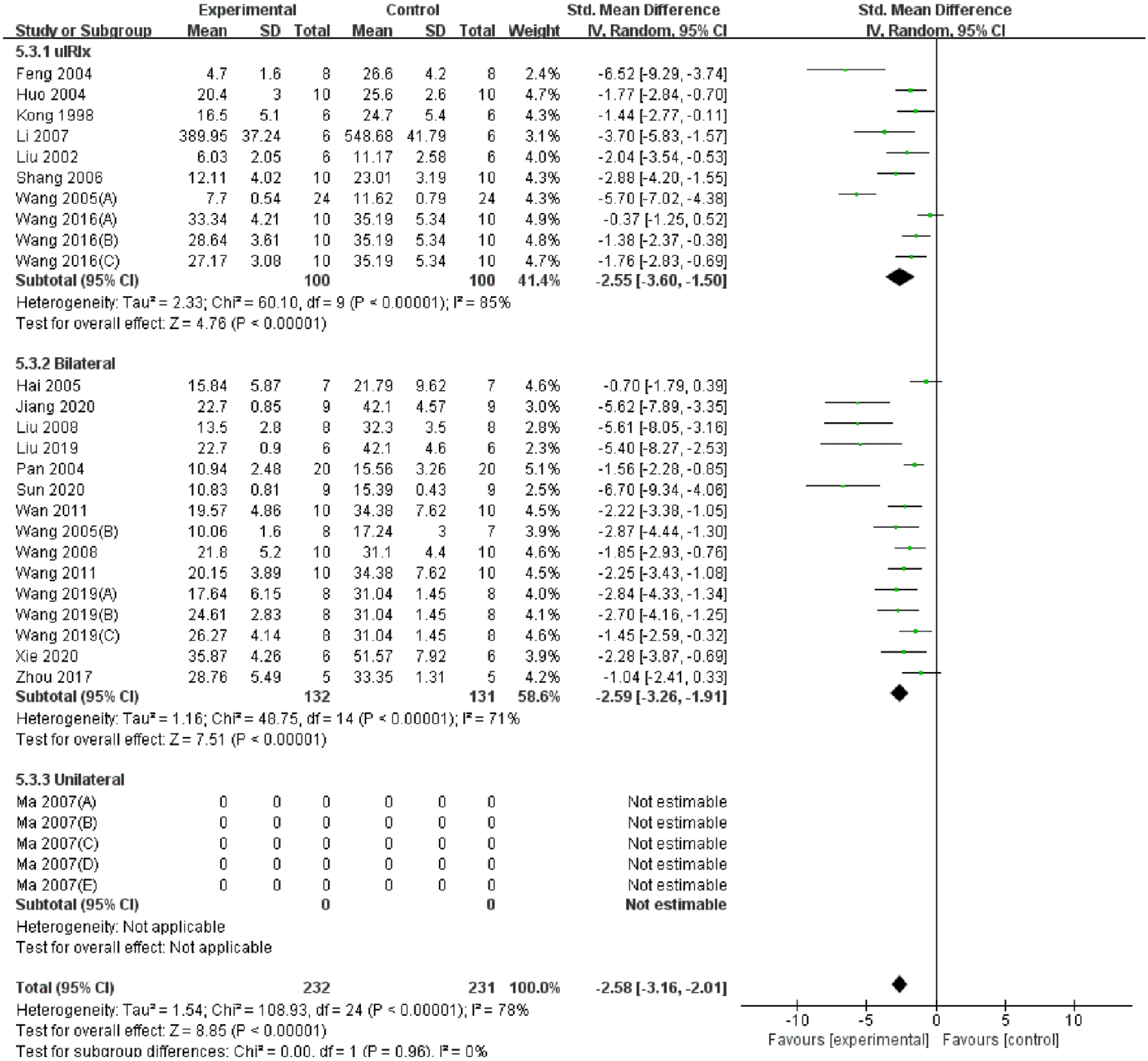

(C)

Study or Subgroup	Experimental Mean	Experimental SD	Experimental Total	Control Mean	Control SD	Control Total	Weight	Std. Mean Difference IV, Random, 95% CI
**2.1.1 pentobarbital sodium**								
Hai 2005	15.84	5.87	7	21.79	9.62	7	3.8%	-0.70 [-1.79, 0.39]
Huo 2004	20.4	3	10	25.6	2.6	10	3.8%	-1.77 [-2.84, -0.70]
Jiang 2020	22.7	0.85	9	42.1	4.57	9	2.7%	-5.62 [-7.89, -3.35]
Kong 1998	16.5	5.1	6	24.7	5.4	6	3.6%	-1.44 [-2.77, -0.11]
Liu 2002	6.03	2.05	6	11.17	2.58	6	3.4%	-2.04 [-3.54, -0.53]
Liu 2008	13.5	2.8	8	32.3	3.5	8	2.5%	-5.61 [-8.05, -3.16]
Liu 2019	22.7	0.9	6	42.1	4.6	6	2.2%	-5.40 [-8.27, -2.53]
Pan 2004	10.94	2.48	20	15.56	3.26	20	4.1%	-1.56 [-2.28, -0.85]
Sun 2020	10.83	0.81	9	15.39	0.43	9	2.4%	-6.70 [-9.34, -4.06]
Wang 2005(B)	10.06	1.6	8	17.24	3	7	3.3%	-2.87 [-4.44, -1.30]
Wang 2008	21.8	5.2	10	31.1	4.4	10	3.8%	-1.85 [-2.93, -0.76]
Wang 2016(A)	33.34	4.21	10	35.19	5.34	10	3.9%	-0.37 [-1.25, 0.52]
Wang 2016(B)	28.64	3.61	10	35.19	5.34	10	3.9%	-1.38 [-2.37, -0.38]
Wang 2016(C)	27.17	3.08	10	35.19	5.34	10	3.8%	-1.76 [-2.83, -0.69]
Wang 2019(A)	17.64	6.15	8	31.04	1.45	8	3.4%	-2.84 [-4.33, -1.34]
Wang 2019(B)	24.61	2.83	8	31.04	1.45	8	3.5%	-2.70 [-4.16, -1.25]
Wang 2019(C)	26.27	4.14	8	31.04	1.45	8	3.7%	-1.45 [-2.59, -0.32]
Xie 2020	35.87	4.26	6	51.57	7.92	6	3.3%	-2.28 [-3.87, -0.69]
**Subtotal (95% CI)**			**159**			**158**	**61.1%**	**-2.26 [-2.85, -1.68]**
Heterogeneity: Tau² = 1.05; Chi² = 60.69, df = 17 (P < 0.00001); I² = 72%								
Test for overall effect: Z = 7.58 (P < 0.00001)								
**2.1.2 Chloral hydrate**								
Li 2007	389.95	37.24	6	548.68	41.79	6	2.8%	-3.70 [-5.83, -1.57]
Ma 2007(A)	12.53	4.39	6	26.35	3.61	6	3.0%	-3.17 [-5.09, -1.26]
Ma 2007(B)	14.22	0.93	6	26.35	3.61	6	2.6%	-4.25 [-6.61, -1.89]
Ma 2007(C)	17.98	1.84	6	26.35	3.61	6	3.2%	-2.70 [-4.43, -0.96]
Ma 2007(D)	31.36	2.55	6	26.35	3.61	6	3.6%	1.48 [0.14, 2.82]
Ma 2007(E)	33.95	2.65	6	26.35	3.61	6	3.3%	2.22 [0.65, 3.78]
Shang 2006	12.11	4.02	10	23.01	3.19	10	3.6%	-2.88 [-4.20, -1.55]
Wan 2011	19.57	4.86	10	34.38	7.62	10	3.7%	-2.22 [-3.38, -1.05]
Wang 2011	20.15	3.89	10	34.38	7.62	10	3.7%	-2.25 [-3.43, -1.08]
Zhou 2017	28.76	5.49	5	33.35	1.31	5	3.5%	-1.04 [-2.41, 0.33]
**Subtotal (95% CI)**			**71**			**71**	**33.1%**	**-1.77 [-3.03, -0.51]**
Heterogeneity: Tau² = 3.46; Chi² = 62.17, df = 9 (P < 0.00001); I² = 86%								
Test for overall effect: Z = 2.75 (P = 0.006)								
**2.1.3 Ketamine hydrochloride**								
Wang 2005(A)	7.7	0.54	24	11.62	0.79	24	3.6%	-5.70 [-7.02, -4.38]
**Subtotal (95% CI)**			**24**			**24**	**3.6%**	**-5.70 [-7.02, -4.38]**
Heterogeneity: Not applicable								
Test for overall effect: Z = 8.48 (P < 0.00001)								
**2.1.4 NA**								
Feng 2004	4.7	1.6	8	26.6	4.2	8	2.3%	-6.52 [-9.29, -3.74]
**Subtotal (95% CI)**			**8**			**8**	**2.3%**	**-6.52 [-9.29, -3.74]**
Heterogeneity: Not applicable								
Test for overall effect: Z = 4.60 (P < 0.00001)								
**Total (95% CI)**			**262**			**261**	**100.0%**	**-2.40 [-3.01, -1.79]**
Heterogeneity: Tau² = 2.23; Chi² = 167.84, df = 29 (P < 0.00001); I² = 83%								
Test for overall effect: Z = 7.73 (P < 0.00001)								
Test for subgroup differences: Chi² = 31.27, df = 3 (P < 0.00001), I² = 90.4%								

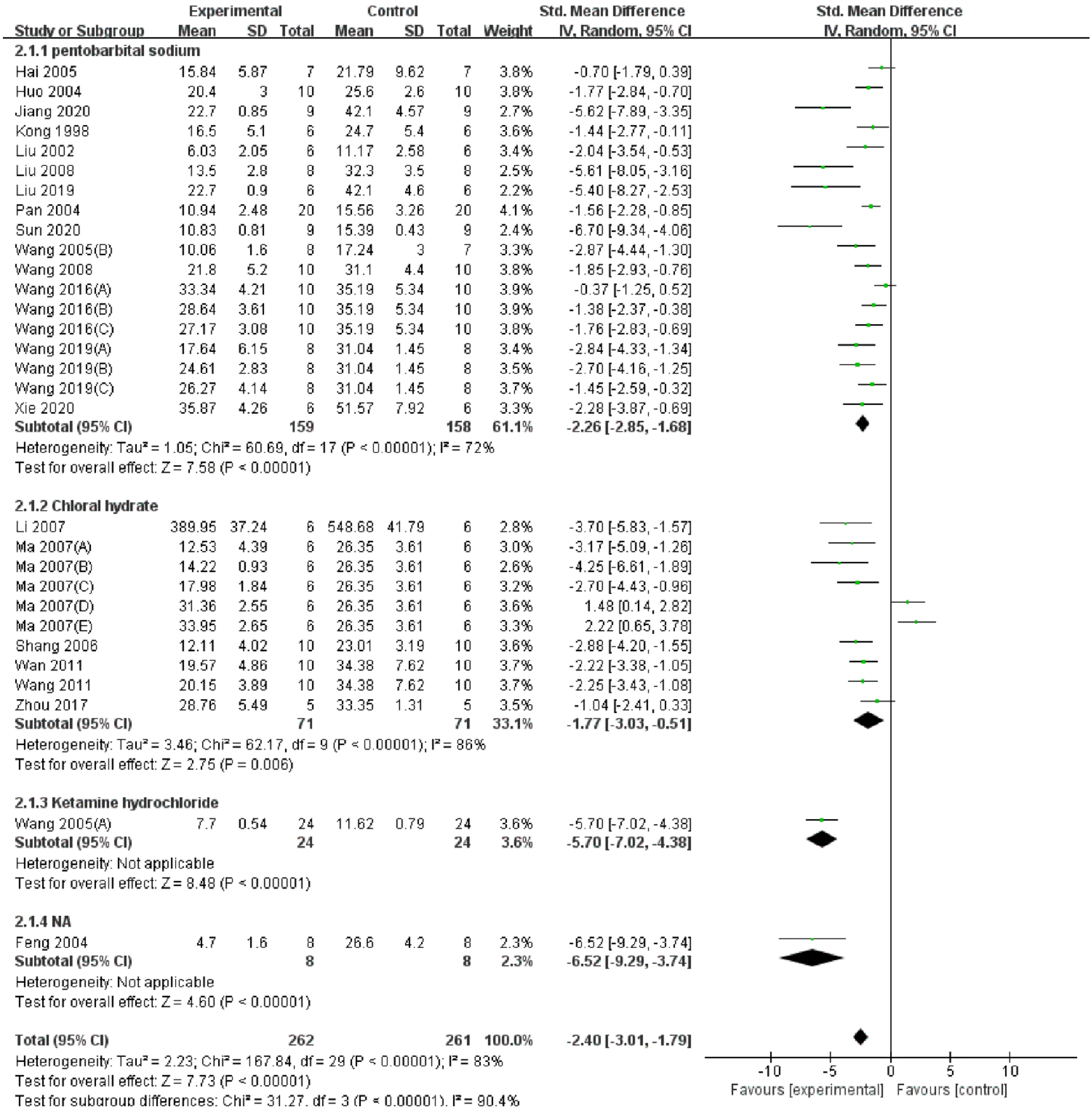

(D)

	Experimental			Control				Std. Mean Difference
**Study or Subgroup**	**Mean**	**SD**	**Total**	**Mean**	**SD**	**Total**	**Weight**	**IV, Random, 95% CI**
**5.5.1 ≤45min**								
Hai 2005	15.84	5.87	7	21.79	9.62	7	3.8%	-0.70 [-1.79, 0.39]
Huo 2004	20.4	3	10	25.6	2.6	10	3.8%	-1.77 [-2.84, -0.70]
Li 2007	389.95	37.24	6	548.68	41.79	6	2.8%	-3.70 [-5.83, -1.57]
Liu 2002	6.03	2.05	6	11.17	2.58	6	3.4%	-2.04 [-3.54, -0.53]
Liu 2008	13.5	2.8	8	32.3	3.5	8	2.5%	-5.61 [-8.05, -3.16]
Liu 2019	22.7	0.9	6	42.1	4.6	6	2.2%	-5.40 [-8.27, -2.53]
Pan 2004	10.94	2.48	20	15.56	3.26	20	4.1%	-1.56 [-2.28, -0.85]
Sun 2020	10.83	0.81	9	15.39	0.43	9	2.4%	-6.70 [-9.34, -4.06]
Wan 2011	19.57	4.86	10	34.38	7.62	10	3.7%	-2.22 [-3.38, -1.05]
Wang 2005(B)	10.06	1.6	8	17.24	3	7	3.3%	-2.87 [-4.44, -1.30]
Wang 2008	21.8	5.2	10	31.1	4.4	10	3.8%	-1.85 [-2.93, -0.76]
Wang 2011	20.15	3.89	10	34.38	7.62	10	3.7%	-2.25 [-3.43, -1.08]
Wang 2019(A)	17.64	6.15	8	31.04	1.45	8	3.4%	-2.84 [-4.33, -1.34]
Wang 2019(B)	24.61	2.83	8	31.04	1.45	8	3.5%	-2.70 [-4.16, -1.25]
Wang 2019(C)	26.27	4.14	8	31.04	1.45	8	3.7%	-1.45 [-2.59, -0.32]
Xie 2020	35.87	4.26	6	51.57	7.92	6	3.3%	-2.28 [-3.87, -0.69]
**Subtotal (95% CI)**			**140**			**139**	**53.5%**	**-2.44 [-3.00, -1.88]**
Heterogeneity: Tau² = 0.75; Chi² = 39.76, df = 15 (P = 0.0005); I² = 62%								
Test for overall effect: Z = 8.49 (P < 0.00001)								
**5.5.2 >45min**								
Feng 2004	4.7	1.6	8	26.6	4.2	8	2.3%	-6.52 [-9.29, -3.74]
Jiang 2020	22.7	0.85	9	42.1	4.57	9	2.7%	-5.62 [-7.89, -3.35]
Kong 1998	16.5	5.1	6	24.7	5.4	6	3.6%	-1.44 [-2.77, -0.11]
Ma 2007(A)	12.53	4.39	6	26.35	3.61	6	3.0%	-3.17 [-5.09, -1.26]
Ma 2007(B)	14.22	0.93	6	26.35	3.61	6	2.6%	-4.25 [-6.61, -1.89]
Ma 2007(C)	17.98	1.84	6	26.35	3.61	6	3.2%	-2.70 [-4.43, -0.96]
Ma 2007(D)	31.36	2.55	6	26.35	3.61	6	3.6%	1.48 [0.14, 2.82]
Ma 2007(E)	33.95	2.65	6	26.35	3.61	6	3.3%	2.22 [0.65, 3.78]
Shang 2006	12.11	4.02	10	23.01	3.19	10	3.6%	-2.88 [-4.20, -1.55]
Wang 2005(A)	7.7	0.54	24	11.62	0.79	24	3.6%	-5.70 [-7.02, -4.38]
Wang 2016(A)	33.34	4.21	10	35.19	5.34	10	3.9%	-0.37 [-1.25, 0.52]
Wang 2016(B)	28.64	3.61	10	35.19	5.34	10	3.9%	-1.38 [-2.37, -0.38]
Wang 2016(C)	27.17	3.08	10	35.19	5.34	10	3.8%	-1.76 [-2.83, -0.69]
Zhou 2017	28.76	5.49	5	33.35	1.31	5	3.5%	-1.04 [-2.41, 0.33]
**Subtotal (95% CI)**			**122**			**122**	**46.5%**	**-2.22 [-3.39, -1.05]**
Heterogeneity: Tau² = 4.33; Chi² = 125.05, df = 13 (P < 0.00001); I² = 90%								
Test for overall effect: Z = 3.71 (P = 0.0002)								
**Total (95% CI)**			**262**			**261**	**100.0%**	**-2.40 [-3.01, -1.79]**
Heterogeneity: Tau² = 2.23; Chi² = 167.84, df = 29 (P < 0.00001); I² = 83%								
Test for overall effect: Z = 7.73 (P < 0.00001)								
Test for subgroup differences: Chi² = 0.11, df = 1 (P = 0.74), I² = 0%								

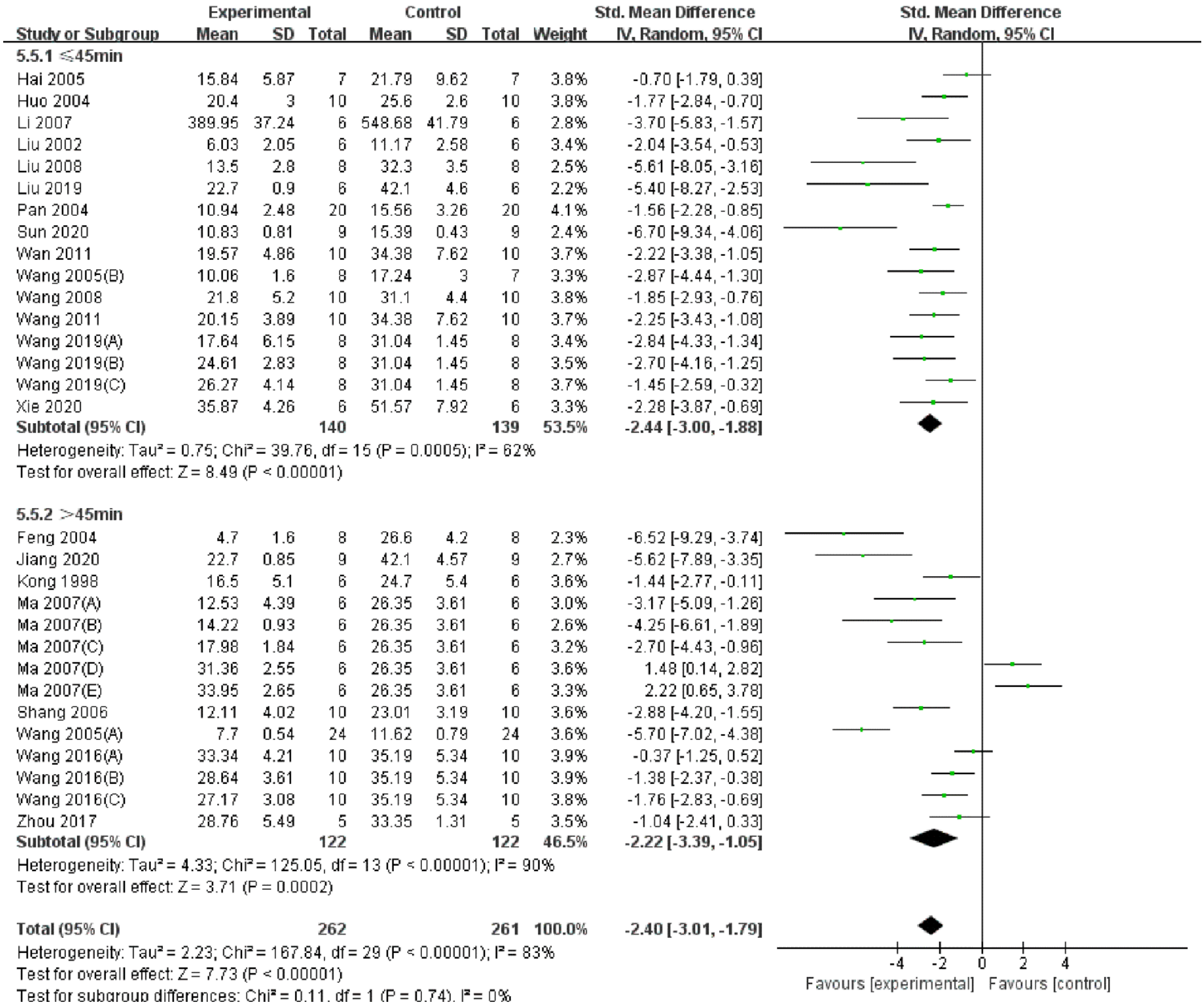

(E)

Study or Subgroup	Experimental Mean	Experimental SD	Experimental Total	Control Mean	Control SD	Control Total	Weight	Std. Mean Difference IV, Random, 95% CI
**5.6.1 Ischemia**								
Feng 2004	4.7	1.6	8	26.6	4.2	8	2.3%	-6.52 [-9.29, -3.74]
Huo 2004	20.4	3	10	25.6	2.6	10	3.8%	-1.77 [-2.84, -0.70]
Li 2007	389.95	37.24	6	548.68	41.79	6	2.8%	-3.70 [-5.83, -1.57]
Liu 2002	6.03	2.05	6	11.17	2.58	6	3.4%	-2.04 [-3.54, -0.53]
Ma 2007(A)	12.53	4.39	6	26.35	3.61	6	3.0%	-3.17 [-5.09, -1.26]
Ma 2007(B)	14.22	0.93	6	26.35	3.61	6	2.6%	-4.25 [-6.61, -1.89]
Ma 2007(C)	17.98	1.84	6	26.35	3.61	6	3.2%	-2.70 [-4.43, -0.96]
Ma 2007(D)	31.36	2.55	6	26.35	3.61	6	3.6%	1.48 [0.14, 2.82]
Ma 2007(E)	33.95	2.65	6	26.35	3.61	6	3.3%	2.22 [0.65, 3.78]
Shang 2006	12.11	4.02	10	23.01	3.19	10	3.6%	-2.88 [-4.20, -1.55]
Wan 2011	19.57	4.86	10	34.38	7.62	10	3.7%	-2.22 [-3.38, -1.05]
Wang 2005(A)	7.7	0.54	24	11.62	0.79	24	3.6%	-5.70 [-7.02, -4.38]
Wang 2005(B)	10.06	1.6	8	17.24	3	7	3.3%	-2.87 [-4.44, -1.30]
Wang 2008	21.8	5.2	10	31.1	4.4	10	3.8%	-1.85 [-2.93, -0.76]
Wang 2011	20.15	3.89	10	34.38	7.62	10	3.7%	-2.25 [-3.43, -1.08]
Xie 2020	35.87	4.26	6	51.57	7.92	6	3.3%	-2.28 [-3.87, -0.69]
**Subtotal (95% CI)**			**138**			**137**	**53.0%**	**-2.42 [-3.40, -1.45]**
Heterogeneity: Tau² = 3.29; Chi² = 104.65, df = 15 (P < 0.00001); I² = 86%								
Test for overall effect: Z = 4.86 (P < 0.00001)								
**5.6.2 Reperfusion**								
Hai 2005	15.84	5.87	7	21.79	9.62	7	3.8%	-0.70 [-1.79, 0.39]
Jiang 2020	22.7	0.85	9	42.1	4.57	9	2.7%	-5.62 [-7.89, -3.35]
Liu 2008	13.5	2.8	8	32.3	3.5	8	2.5%	-5.61 [-8.05, -3.16]
Liu 2019	22.7	0.9	6	42.1	4.6	6	2.2%	-5.40 [-8.27, -2.53]
Sun 2020	10.83	0.81	9	15.39	0.43	9	2.4%	-6.70 [-9.34, -4.06]
Wang 2019(A)	17.64	6.15	8	31.04	1.45	8	3.4%	-2.84 [-4.33, -1.34]
Wang 2019(B)	24.61	2.83	8	31.04	1.45	8	3.5%	-2.70 [-4.16, -1.25]
Wang 2019(C)	26.27	4.14	8	31.04	1.45	8	3.7%	-1.45 [-2.59, -0.32]
Zhou 2017	28.76	5.49	5	33.35	1.31	5	3.5%	-1.04 [-2.41, 0.33]
**Subtotal (95% CI)**			**68**			**68**	**27.7%**	**-3.25 [-4.55, -1.94]**
Heterogeneity: Tau² = 3.08; Chi² = 44.79, df = 8 (P < 0.00001); I² = 82%								
Test for overall effect: Z = 4.88 (P < 0.00001)								
**5.6.3 Continuous**								
Kong 1998	16.5	5.1	6	24.7	5.4	6	3.6%	-1.44 [-2.77, -0.11]
Pan 2004	10.94	2.48	20	15.56	3.26	20	4.1%	-1.56 [-2.28, -0.85]
Wang 2016(A)	33.34	4.21	10	35.19	5.34	10	3.9%	-0.37 [-1.25, 0.52]
Wang 2016(B)	28.64	3.61	10	35.19	5.34	10	3.9%	-1.38 [-2.37, -0.38]
Wang 2016(C)	27.17	3.08	10	35.19	5.34	10	3.8%	-1.76 [-2.83, -0.69]
**Subtotal (95% CI)**			**56**			**56**	**19.2%**	**-1.28 [-1.78, -0.77]**
Heterogeneity: Tau² = 0.09; Chi² = 5.54, df = 4 (P = 0.24); I² = 28%								
Test for overall effect: Z = 4.95 (P < 0.00001)								
**Total (95% CI)**			**262**			**261**	**100.0%**	**-2.40 [-3.01, -1.79]**
Heterogeneity: Tau² = 2.23; Chi² = 167.84, df = 29 (P < 0.00001); I² = 83%								
Test for overall effect: Z = 7.73 (P < 0.00001)								
Test for subgroup differences: Chi² = 10.18, df = 2 (P = 0.006), I² = 80.4%								

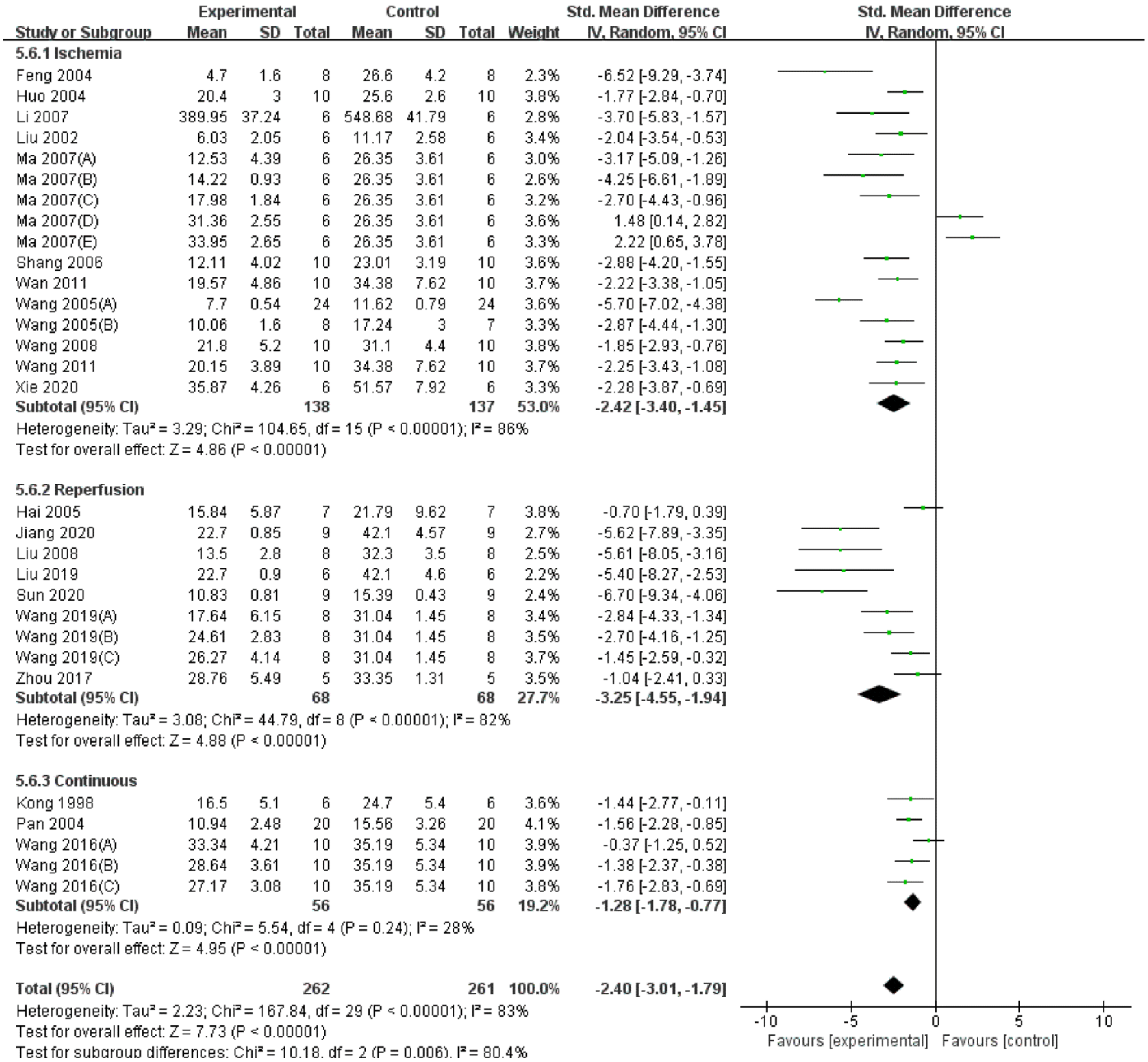

(F)

Study or Subgroup	Experimental Mean	Experimental SD	Experimental Total	Control Mean	Control SD	Control Total	Weight	Std. Mean Difference IV, Random, 95% CI
**5.7.1 ≤50mg/kg**								
Jiang 2020	22.7	0.85	9	42.1	4.57	9	2.7%	-5.62 [-7.89, -3.35]
Kong 1998	16.5	5.1	6	24.7	5.4	6	3.6%	-1.44 [-2.77, -0.11]
Li 2007	389.95	37.24	6	548.68	41.79	6	2.8%	-3.70 [-5.83, -1.57]
Liu 2008	13.5	2.8	8	32.3	3.5	8	2.5%	-5.61 [-8.05, -3.16]
Liu 2019	22.7	0.9	6	42.1	4.6	6	2.2%	-5.40 [-8.27, -2.53]
Ma 2007(A)	12.53	4.39	6	26.35	3.61	6	3.0%	-3.17 [-5.09, -1.26]
Ma 2007(B)	14.22	0.93	6	26.35	3.61	6	2.6%	-4.25 [-6.61, -1.89]
Ma 2007(C)	17.98	1.84	6	26.35	3.61	6	3.2%	-2.70 [-4.43, -0.96]
Pan 2004	10.94	2.48	20	15.56	3.26	20	4.1%	-1.56 [-2.28, -0.85]
Sun 2020	10.83	0.81	9	15.39	0.43	9	2.4%	-6.70 [-9.34, -4.06]
Wan 2011	19.57	4.86	10	34.38	7.62	10	3.7%	-2.22 [-3.38, -1.05]
Wang 2005(A)	7.7	0.54	24	11.62	0.79	24	3.6%	-5.70 [-7.02, -4.38]
Wang 2008	21.8	5.2	10	31.1	4.4	10	3.8%	-1.85 [-2.93, -0.76]
Wang 2011	20.15	3.89	10	34.38	7.62	10	3.7%	-2.25 [-3.43, -1.08]
Wang 2016(A)	33.34	4.21	10	35.19	5.34	10	3.9%	-0.37 [-1.25, 0.52]
Wang 2016(B)	28.64	3.61	10	35.19	5.34	10	3.9%	-1.38 [-2.37, -0.38]
Wang 2016(C)	27.17	3.08	10	35.19	5.34	10	3.8%	-1.76 [-2.83, -0.69]
Wang 2019(A)	17.64	6.15	8	31.04	1.45	8	3.4%	-2.84 [-4.33, -1.34]
Wang 2019(B)	24.61	2.83	8	31.04	1.45	8	3.5%	-2.70 [-4.16, -1.25]
Wang 2019(C)	26.27	4.14	8	31.04	1.45	8	3.7%	-1.45 [-2.59, -0.32]
**Subtotal (95% CI)**			**190**			**190**	**66.1%**	**-2.84 [-3.52, -2.16]**
Heterogeneity: Tau² = 1.75; Chi² = 92.44, df = 19 (P < 0.00001); I² = 79%								
Test for overall effect: Z = 8.17 (P < 0.00001)								
**5.7.2 >50mg/kg**								
Feng 2004	4.7	1.6	8	26.6	4.2	8	2.3%	-6.52 [-9.29, -3.74]
Hai 2005	15.84	5.87	7	21.79	9.62	7	3.8%	-0.70 [-1.79, 0.39]
Huo 2004	20.4	3	10	25.6	2.6	10	3.8%	-1.77 [-2.84, -0.70]
Liu 2002	6.03	2.05	6	11.17	2.58	6	3.4%	-2.04 [-3.54, -0.53]
Ma 2007(D)	31.36	2.55	6	26.35	3.61	6	3.6%	1.48 [0.14, 2.82]
Ma 2007(E)	33.95	2.65	6	26.35	3.61	6	3.3%	2.22 [0.65, 3.78]
Shang 2006	12.11	4.02	10	23.01	3.19	10	3.6%	-2.88 [-4.20, -1.55]
Wang 2005(B)	10.06	1.6	8	17.24	3	7	3.3%	-2.87 [-4.44, -1.30]
Xie 2020	35.87	4.26	6	51.57	7.92	6	3.3%	-2.28 [-3.87, -0.69]
Zhou 2017	28.76	5.49	5	33.35	1.31	5	3.5%	-1.04 [-2.41, 0.33]
**Subtotal (95% CI)**			**72**			**71**	**33.9%**	**-1.49 [-2.68, -0.30]**
Heterogeneity: Tau² = 3.09; Chi² = 63.05, df = 9 (P < 0.00001); I² = 86%								
Test for overall effect: Z = 2.45 (P = 0.01)								
**Total (95% CI)**			**262**			**261**	**100.0%**	**-2.40 [-3.01, -1.79]**
Heterogeneity: Tau² = 2.23; Chi² = 167.84, df = 29 (P < 0.00001); I² = 83%								
Test for overall effect: Z = 7.73 (P < 0.00001)								
Test for subgroup differences: Chi² = 3.68, df = 1 (P = 0.05), I² = 72.9%								

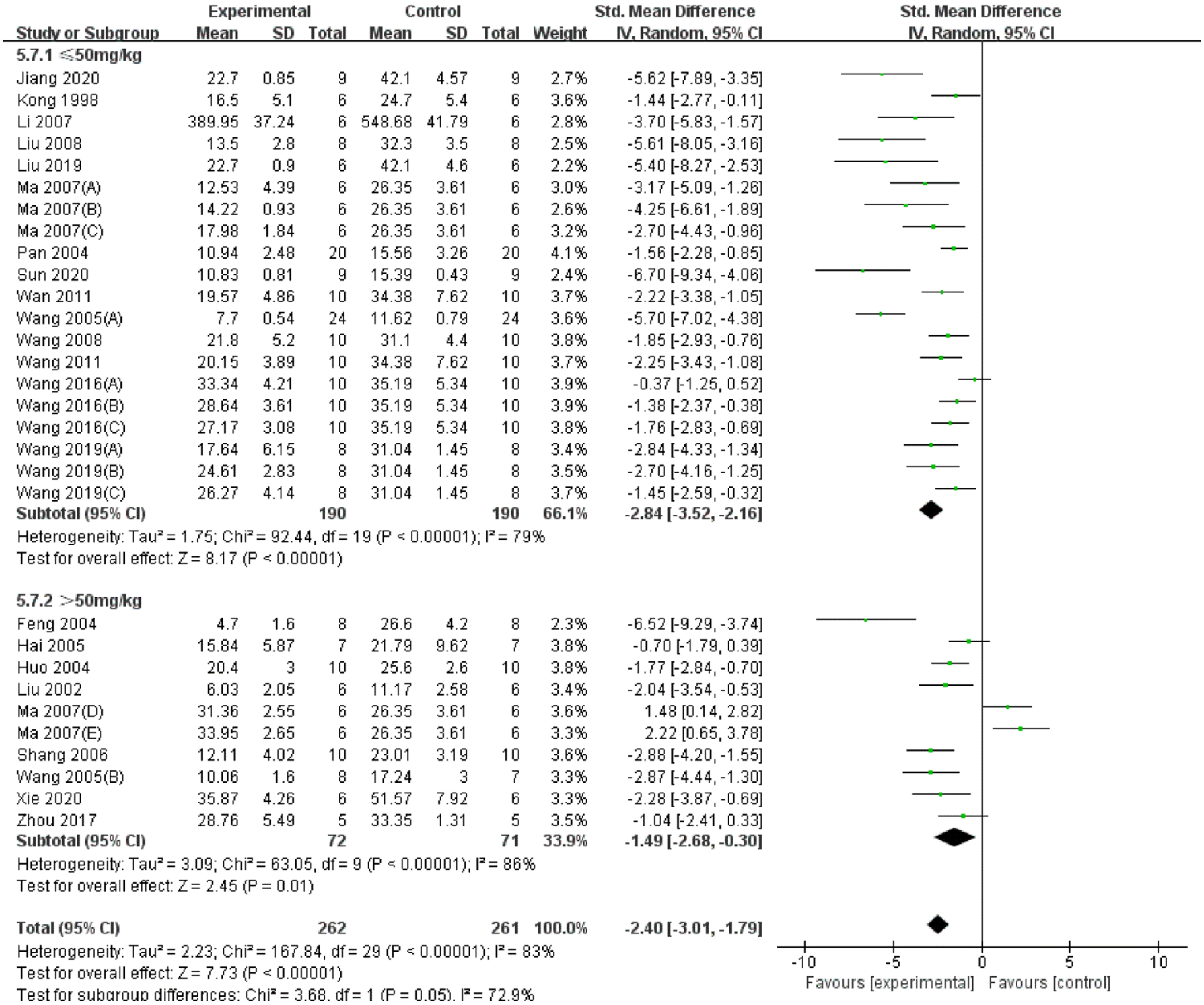

(G)

Study or Subgroup	Experimental Mean	Experimental SD	Experimental Total	Control Mean	Control SD	Control Total	Weight	Std. Mean Difference IV, Random, 95% CI
**5.8.1 IP**								
Feng 2011	4.7	1.6	8	26.6	4.2	8	2.3%	-6.52 [-9.29, -3.74]
Huo 2004	20.4	3	10	25.6	2.6	10	4.2%	-1.77 [-2.84, -0.70]
Jiang 2020	22.7	0.85	9	42.1	4.57	9	2.8%	-5.62 [-7.89, -3.35]
Liu 2002	6.03	2.05	6	11.17	2.58	6	3.7%	-2.04 [-3.54, -0.53]
Liu 2008	13.5	2.8	8	32.3	3.5	8	2.6%	-5.61 [-8.05, -3.16]
Liu 2019	22.7	0.9	6	42.1	4.6	6	2.2%	-5.40 [-8.27, -2.53]
Pan 2004	10.94	2.48	20	15.56	3.26	20	4.5%	-1.56 [-2.28, -0.85]
Sun 2020	10.83	0.81	9	15.39	0.43	9	2.4%	-6.70 [-9.34, -4.06]
Wan 2011	19.57	4.86	10	34.38	7.62	10	4.1%	-2.22 [-3.38, -1.05]
Wang 2008	21.8	5.2	10	31.1	4.4	10	4.1%	-1.85 [-2.93, -0.76]
Wang 2011	20.15	3.89	10	34.38	7.62	10	4.0%	-2.25 [-3.43, -1.08]
Wang 2016(A)	33.34	4.21	10	35.19	5.34	10	4.4%	-0.37 [-1.25, 0.52]
Wang 2016(B)	28.64	3.61	10	35.19	5.34	10	4.2%	-1.38 [-2.37, -0.38]
Wang 2016(C)	27.17	3.08	10	35.19	5.34	10	4.2%	-1.76 [-2.83, -0.69]
Wang 2019(A)	17.64	6.15	8	31.04	1.45	8	3.7%	-2.84 [-4.33, -1.34]
Wang 2019(B)	24.61	2.83	8	31.04	1.45	8	3.7%	-2.70 [-4.16, -1.25]
Wang 2019(C)	26.27	4.14	8	31.04	1.45	8	4.1%	-1.45 [-2.59, -0.32]
Xie 2020	35.87	4.26	6	51.57	7.92	6	3.6%	-2.28 [-3.87, -0.69]
Zhou 2017	28.76	5.49	5	33.35	1.31	5	3.8%	-1.04 [-2.41, 0.33]
**Subtotal (95% CI)**			**171**			**171**	**68.5%**	**-2.44 [-3.04, -1.85]**
Heterogeneity: Tau² = 1.15; Chi² = 67.26, df = 18 (P < 0.00001); I² = 73%								
Test for overall effect: Z = 8.08 (P < 0.00001)								
**5.8.2 IV**								
Hai 2005	15.84	5.87	7	21.79	9.62	7	4.1%	-0.70 [-1.79, 0.39]
Kong 1998	16.5	5.1	6	24.7	5.4	6	3.9%	-1.44 [-2.77, -0.11]
Li 2007	389.95	37.24	6	548.68	41.79	6	2.9%	-3.70 [-5.83, -1.57]
Ma 2007(A)	12.53	4.39	6	26.35	3.61	6	3.2%	-3.17 [-5.09, -1.26]
Ma 2007(B)	14.22	0.93	6	26.35	3.61	6	2.7%	-4.25 [-6.61, -1.89]
Ma 2007(C)	17.98	1.84	6	26.35	3.61	6	3.4%	-2.70 [-4.43, -0.96]
Ma 2007(D)	31.36	2.55	6	26.35	3.61	6	3.9%	1.48 [0.14, 2.82]
Ma 2007(E)	33.95	2.65	6	26.35	3.61	6	3.6%	2.22 [0.65, 3.78]
**Subtotal (95% CI)**			**49**			**49**	**27.6%**	**-1.41 [-2.94, 0.12]**
Heterogeneity: Tau² = 4.11; Chi² = 51.91, df = 7 (P < 0.00001); I² = 87%								
Test for overall effect: Z = 1.81 (P = 0.07)								
**5.8.3 Intragastric**								
Shang 2006	12.11	4.02	10	23.01	3.19	10	3.9%	-2.88 [-4.20, -1.55]
**Subtotal (95% CI)**			**10**			**10**	**3.9%**	**-2.88 [-4.20, -1.55]**
Heterogeneity: Not applicable								
Test for overall effect: Z = 4.25 (P < 0.0001)								
**Total (95% CI)**			**230**			**230**	**100.0%**	**-2.22 [-2.80, -1.64]**
Heterogeneity: Tau² = 1.82; Chi² = 132.94, df = 27 (P < 0.00001); I² = 80%								
Test for overall effect: Z = 7.48 (P < 0.00001)								
Test for subgroup differences: Chi² = 2.12, df = 2 (P = 0.35), I² = 5.6%								

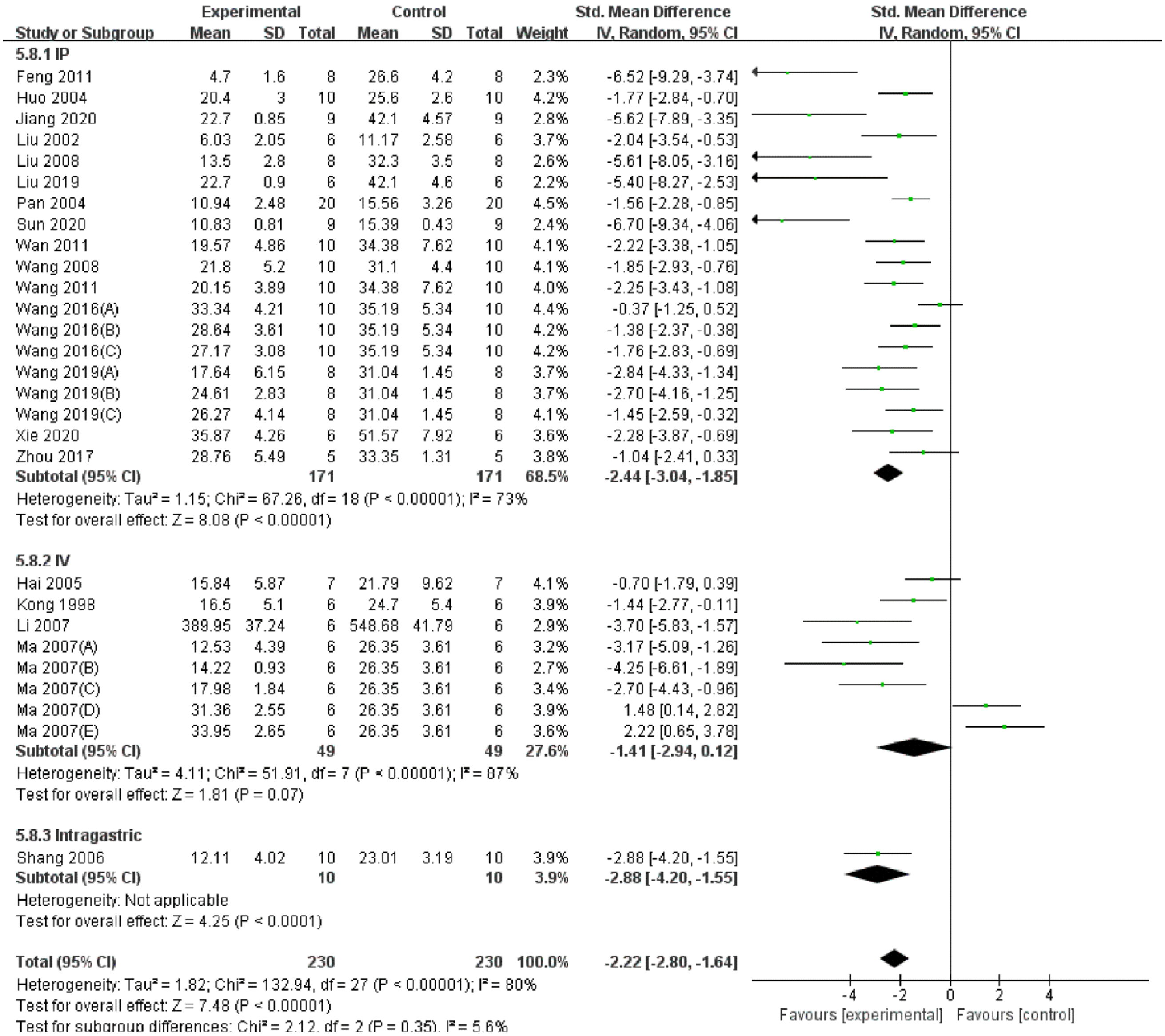

(H)

Study or Subgroup	Experimental Mean	Experimental SD	Experimental Total	Control Mean	Control SD	Control Total	Weight	Std. Mean Difference IV, Random, 95% CI
**5.9.1 Single**								
Feng 2004	4.7	1.6	8	26.6	4.2	8	2.3%	-6.52 [-9.29, -3.74]
Hai 2005	15.84	5.87	7	21.79	9.62	7	3.8%	-0.70 [-1.79, 0.39]
Huo 2004	20.4	3	10	25.6	2.6	10	3.8%	-1.77 [-2.84, -0.70]
Li 2007	389.95	37.24	6	548.68	41.79	6	2.8%	-3.70 [-5.83, -1.57]
Liu 2002	6.03	2.05	6	11.17	2.58	6	3.4%	-2.04 [-3.54, -0.53]
Ma 2007(A)	12.53	4.39	6	26.35	3.61	6	3.0%	-3.17 [-5.09, -1.26]
Ma 2007(B)	14.22	0.93	6	26.35	3.61	6	2.6%	-4.25 [-6.61, -1.89]
Ma 2007(C)	17.98	1.84	6	26.35	3.61	6	3.2%	-2.70 [-4.43, -0.96]
Ma 2007(D)	31.36	2.55	6	26.35	3.61	6	3.6%	1.48 [0.14, 2.82]
Ma 2007(E)	33.95	2.65	6	26.35	3.61	6	3.3%	2.22 [0.65, 3.78]
Wan 2011	19.57	4.86	10	34.38	7.62	10	3.7%	-2.22 [-3.38, -1.05]
Wang 2005(B)	10.06	1.6	8	17.24	3	7	3.3%	-2.87 [-4.44, -1.30]
Wang 2011	20.15	3.89	10	34.38	7.62	10	3.7%	-2.25 [-3.43, -1.08]
Wang 2019(A)	17.64	6.15	8	31.04	1.45	8	3.4%	-2.84 [-4.33, -1.34]
Wang 2019(B)	24.61	2.83	8	31.04	1.45	8	3.5%	-2.70 [-4.16, -1.25]
Wang 2019(C)	26.27	4.14	8	31.04	1.45	8	3.7%	-1.45 [-2.59, -0.32]
Zhou 2017	28.76	5.49	5	33.35	1.31	5	3.5%	-1.04 [-2.41, 0.33]
**Subtotal (95% CI)**			**124**			**123**	**56.7%**	**-1.98 [-2.78, -1.18]**
Heterogeneity: Tau² = 2.18; Chi² = 80.82, df = 16 (P < 0.00001); I² = 80%								
Test for overall effect: Z = 4.85 (P < 0.00001)								
**5.9.2 Multiple**								
Jiang 2020	22.7	0.85	9	42.1	4.57	9	2.7%	-5.62 [-7.89, -3.35]
Kong 1998	16.5	5.1	6	24.7	5.4	6	3.6%	-1.44 [-2.77, -0.11]
Liu 2008	13.5	2.8	8	32.3	3.5	8	2.5%	-5.61 [-8.05, -3.16]
Liu 2019	22.7	0.9	6	42.1	4.6	6	2.2%	-5.40 [-8.27, -2.53]
Pan 2004	10.94	2.48	20	15.56	3.26	20	4.1%	-1.56 [-2.28, -0.85]
Shang 2006	12.11	4.02	10	23.01	3.19	10	3.6%	-2.88 [-4.20, -1.55]
Sun 2020	10.83	0.81	9	15.39	0.43	9	2.4%	-6.70 [-9.34, -4.06]
Wang 2005(A)	7.7	0.54	24	11.62	0.79	24	3.6%	-5.70 [-7.02, -4.38]
Wang 2008	21.8	5.2	10	31.1	4.4	10	3.8%	-1.85 [-2.93, -0.76]
Wang 2016(A)	33.34	4.21	10	35.19	5.34	10	3.9%	-0.37 [-1.25, 0.52]
Wang 2016(B)	28.64	3.61	10	35.19	5.34	10	3.9%	-1.38 [-2.37, -0.38]
Wang 2016(C)	27.17	3.08	10	35.19	5.34	10	3.8%	-1.76 [-2.83, -0.69]
Xie 2020	35.87	4.26	6	51.57	7.92	6	3.3%	-2.28 [-3.87, -0.69]
**Subtotal (95% CI)**			**138**			**138**	**43.3%**	**-2.96 [-3.92, -2.00]**
Heterogeneity: Tau² = 2.47; Chi² = 84.26, df = 12 (P < 0.00001); I² = 86%								
Test for overall effect: Z = 6.04 (P < 0.00001)								
**Total (95% CI)**			**262**			**261**	**100.0%**	**-2.40 [-3.01, -1.79]**
Heterogeneity: Tau² = 2.23; Chi² = 167.84, df = 29 (P < 0.00001); I² = 83%								
Test for overall effect: Z = 7.73 (P < 0.00001)								
Test for subgroup differences: Chi² = 2.37, df = 1 (P = 0.12), I² = 57.8%								

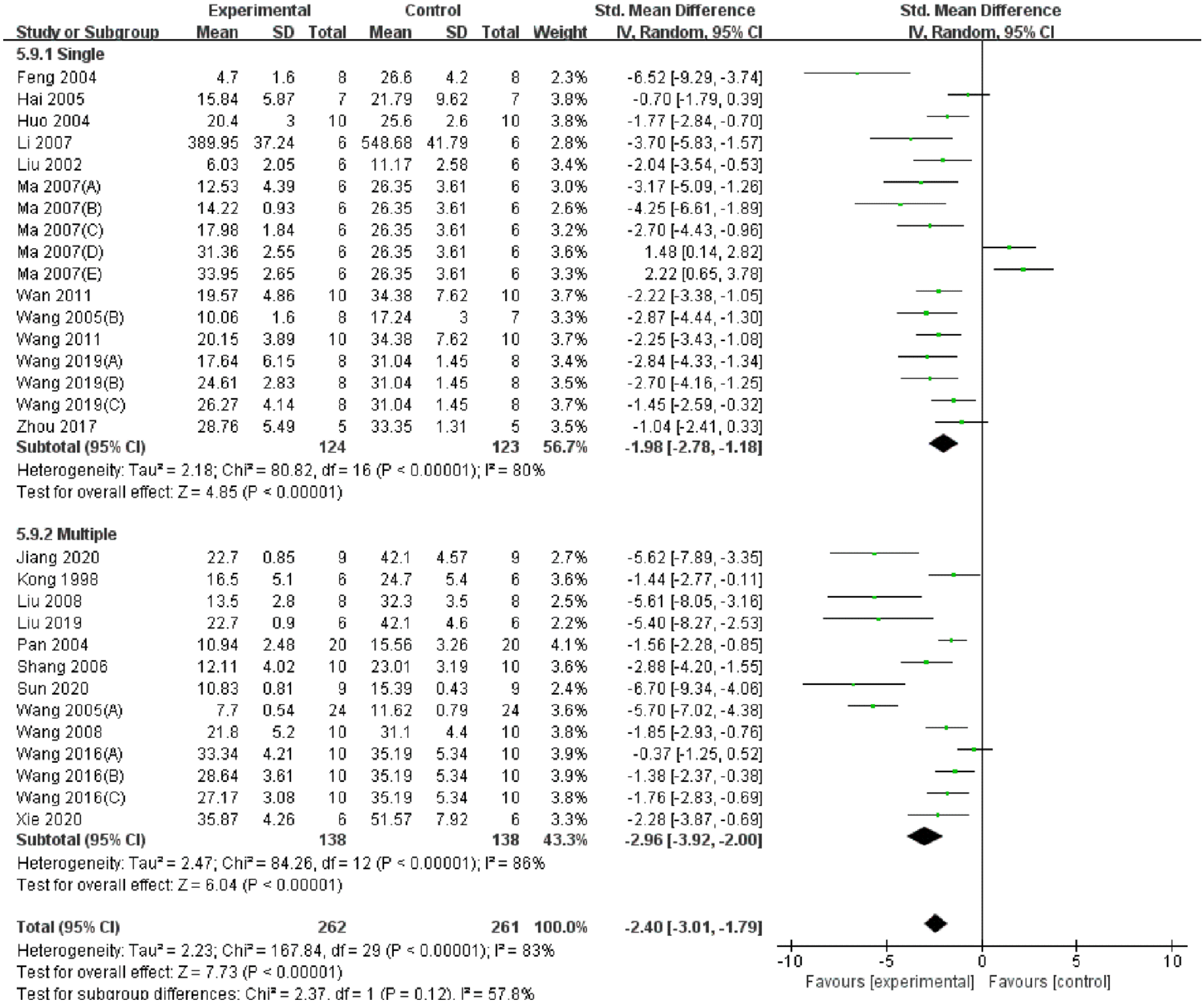

Supplement: Supplementary file 2 [file Image1.pdf]
